# Phylogenomic Investigation of Increasing Fluoroquinolone Resistance among Belgian Cases of Shigellosis between 2013 and 2018 Indicates Both Travel-Related Imports and Domestic Circulation

**DOI:** 10.3390/microorganisms9040767

**Published:** 2021-04-06

**Authors:** Bert Bogaerts, Raf Winand, Julien Van Braekel, Wesley Mattheus, Sigrid C. J. De Keersmaecker, Nancy H. C. Roosens, Kathleen Marchal, Kevin Vanneste, Pieter-Jan Ceyssens

**Affiliations:** 1 Transversal activities in Applied Genomics, Sciensano, 1050 Brussels, Belgium; Bert.Bogaerts@Sciensano.be (B.B.); raf.winand@sciensano.be (R.W.); julien.vanbraekel@sciensano.be (J.V.B.); sigrid.dekeersmaecker@sciensano.be (S.C.J.D.K.); Nancy.Roosens@sciensano.be (N.H.C.R.); kevin.vanneste@sciensano.be (K.V.); 2 Department of Plant Biotechnology and Bioinformatics, Ghent University, 9000 Ghent, Belgium; kathleen.marchal@ugent.be; 3 Bacterial Diseases, Sciensano, 1050 Brussels, Belgium; Wesley.Mattheus@sciensano.be; 4 Department of Information Technology, IDLab, imec, Ghent University, 9000 Ghent, Belgium; 5 Department of Genetics, University of Pretoria, Pretoria 0001, South Africa

**Keywords:** *Shigella*, fluoroquinolones, ciprofloxacin, public health, national reference center, antimicrobial resistance

## Abstract

Shigellosis is an acute enteric infection caused mainly by the species *Shigella flexneri* and *Shigella sonnei*. Since surveillance of these pathogens indicated an increase in ciprofloxacin-resistant samples collected in Belgium between 2013 and 2018, a subset of 148 samples was analyzed with whole genome sequencing (WGS) to investigate their dispersion and underlying genomic features associated with ciprofloxacin resistance. A comparison between observed phenotypes and WGS-based resistance prediction to ciprofloxacin revealed perfect correspondence for all samples. Core genome multi-locus sequence typing and single nucleotide polymorphism-typing were used for phylogenomic investigation to characterize the spread of these infections within Belgium, supplemented with data from international reference collections to place the Belgian isolates within their global context. For *S. flexneri*, substantial diversity was observed with ciprofloxacin-resistant isolates assigned to several phylogenetic groups. Besides travel-related imports, several clusters of highly similar Belgian isolates could not be linked directly to international travel suggesting the presence of domestically circulating strains. For *S. sonnei*, Belgian isolates were all limited to lineage III, and could often be traced back to travel to countries in Asia and Africa, sometimes followed by domestic circulation. For both species, several clusters of isolates obtained exclusively from male patients were observed. Additionally, we illustrated the limitations of conventional serotyping of *S. flexneri*, which was impacted by serotype switching. This study contributes to a better understanding of the spread of shigellosis within Belgium and internationally, and highlights the added value of WGS for the surveillance of this pathogen.

## 1. Introduction

Shigellosis is an acute enteric infection caused by the species *Shigella dysenteriae, S. flexneri, S. boydii*, and *S. sonnei*, and transmitted via the oral-fecal route. While infection with all *Shigella* species provokes similar syndromes such as fever, bloody diarrhea, and nausea, their disease severity and geographical distribution differ. In Belgium and other developed countries [1], the most commonly observed species in clinical cases is *S. sonnei,* followed by *S. flexneri*. In developing countries, shigellosis is an important cause of diarrheal disease in children under five years resulting globally in approximately 160,000 deaths per year [2]. In developed countries, *Shigella* infections were traditionally considered as mostly travel-related, although recent surveillance data indicate a shift towards domestically circulating sublineages in patient-risk groups [3,4,5].

The evolution of *Shigella* spp. from the extremely diverse *Escherichia coli* species to a highly specialized and human-restricted group of pathogens occurred via convergent horizontal gene transfer events and gene losses [6,7]. In the last decade, phylogenetic studies have shown that serologically defined *Shigella* spp. comprise three clusters (C1, C2, and C3) embedded within, but distinct from *E. coli* lineages A, B1, and E [8]. These genomic elements were not obtained at a single point in time from a single ancestral species but were rather acquired independently on multiple occasions from multiple ancestors, and encode genes for invasive infection, siderophores, and bactericidal and immune evasion [9]. Massive sequencing efforts have unraveled the global population structures of *S. sonnei* and *S. flexneri*, revealing both global lineages but also lineages specific to the United States of America, Latin America, Australia, the United Kingdom, and France [4,5,7,10,11,12]. *S. sonnei* emerged 400 years ago in Europe by acquiring its O-antigen cluster from *Plesiomonas shigelloides* [11,13]. It is a single-serological type and rapidly evolving species that became globally dominant through competitive advantages by taking up antimicrobial resistance (AMR) genes and colicin-producing plasmids. Currently, lineage III is the dominating *S. sonnei* species in developed countries, stirred by strong selective pressure from localized antimicrobial use [11,14,15]. In contrast, *S. flexneri* is more prevalent in developing countries and encodes a wide variety of O-antigen modifying enzymes that are often encoded by prophages or plasmids [16,17]. *S. flexneri* consists of seven phylogenetic groups (PGs) that are not completely concordant with the conventional serotype classification due to consecutive serotype switching events. In contrast to *S. sonnei*, the accumulation of AMR genes did not replace antibiotic-sensitive lineages of *S. flexneri*, which continue to circulate locally in endemic regions [10].

While shigellosis is typically a self-limiting disease, appropriate treatment can shorten the duration of illness and bacterial shedding, and therefore prevent transmission [18]. For considerable time, the World Health Organization (WHO) recommended the use of fluoroquinolones for treatment. These synthetic molecules are broad-spectrum antibiotics targeting the DNA gyrase (GyrA/B) and topoisomerase IV (ParC/E) enzymes, which are essential for DNA supercoiling and chromosome segregation, respectively [19]. Stepwise accumulations of spontaneous mutations have however been observed in the quinolone resistance determining regions of *gyrA* and *parC* [20]. Fluoroquinolone-resistant *S. sonnei* emerged from a single common ancestor in South Asia through consecutive *gyrA* S83L, followed by *parC* S80I and *gyrA* D87G mutations [20], before spreading across Asia and being disseminated intercontinentally. Additionally, *Shigella* strains harboring plasmid-encoded *qnr* genes can replicate efficiently in high concentrations of ciprofloxacin, giving them a competitive advantage against fluoroquinolone-susceptible strains due to enhanced shedding and transmission [15].

**Figure 1:**
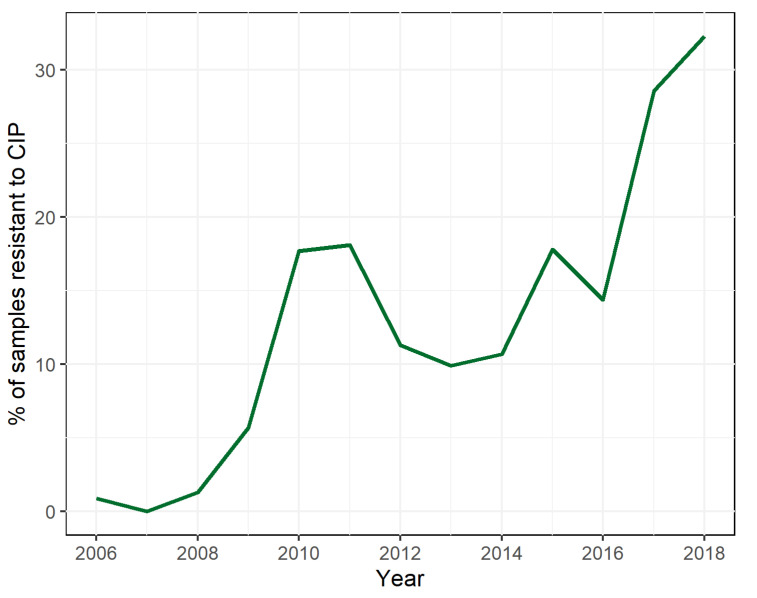
Evolution of high-level ciprofloxacin-resistant samples received by the National Reference Centre (NRC) for shigellosis in Belgium between 2006 and 2018. The *X*-axis denotes the year, the *Y*-axis denotes the percentage of *Shigella* samples received by the NRC that exhibited high-level phenotypic resistance to ciprofloxacin. All samples received by the NRC were included, covering the four *Shigella* subspecies. Abbreviations: ciprofloxacin (CIP).

National surveillance in Belgium is performed by the National Reference Centre (NRC) for shigellosis, which receives annually approximately 400 *Shigella* cultures on a voluntary basis from peripheral laboratories [21]. Traditionally, *Shigella* spp. are subtyped using biochemical, mobility, and serological assays, and their phenotypic resistance profiles are recorded against a panel of 14 antibiotics [22] but it has become clear that serotype-based clustering is not a good basis for public health surveillance [23]. Whole genome sequencing (WGS) has been a powerful tool to study the spread and evolution of these pathogens, as WGS-based methods have enabled characterizing the global spread of both *S. flexneri* [10] and *S. sonnei* [11,24], and the added value for routine surveillance has also been highlighted [23,25]. In contrast to accessed on conventional methods, WGS offers the advantage of providing all required information in a single assay and at a nucleotide-level resolution. With respect to ciprofloxacin-resistance, WGS allows detecting the presence of both chromosomal point mutations [26] and *qnr* genes [27,28] that are commonly associated with resistance to fluoroquinolones. In light of increasing national levels of resistance to fluoroquinolones among *Shigella* spp. observed in Belgium (Figure 1), we report here a genomics-based survey to investigate its origin and spread.

## 2. Material and Methods

### 2.1. Bacterial Isolates

In Belgium, peripheral clinical laboratories collect *Shigella* isolates from patients and send them on a voluntary basis to the NRC at Sciensano for biochemical- and serotyping, and assessment of antimicrobial resistance. Data on residence, gender, age, and travel history of cases are voluntarily provided by the submitting lab. Until 2017, antibiograms were effectuated with disk diffusion with low-level resistance to ciprofloxacin being determined using the 5 µg pefloxacin disk [29]. Since 2017, the NRC switched to broth microdilution (Sensititre™, Thermo Scientific, Cleveland, OH, USA), with ciprofloxacin resistance measured in a twofold dilution series ranging from 0.015 to 8 µg/mL. We used the European Committee on Antimicrobial Susceptibility Testing (EUCAST) guidelines and breakpoints for classifying *Shigella* isolates as either susceptible or resistant to ciprofloxacin [30]. Resistant samples were then further subdivided as either low- or high-level resistant based on our experience, with low-level resistant strains still being treatable by administering high doses of ciprofloxacin. More specifically, low-level ciprofloxacin resistance was defined as pefloxacin disk diameters between 10 and 24 mm for samples collected before 2017 and minimum inhibitory concentration (MIC) values between 0.5 and 4 µg/mL for samples collected since 2017. Strains that displayed either ciprofloxacin (5 µg) disk diameters < 12 mm and/or MIC values > 4 µg/mL were considered as high-level resistant. *S. flexneri* (*n* = 62) and *S. sonnei* (*n* = 91) isolates were selected from Belgian patients sampled between 2013-2018, of which 81 were high-level and 44 low-level resistant to ciprofloxacin (Supplementary Table S1). The remaining 28 isolates were fully susceptible. Isolates with travel information (including confirmed absence of travel) were preferably selected.

### 2.2. Whole-Genome Sequencing and Dataset Collection

Bacterial gDNA was extracted using the MgC Bacterial DNA Kit™ with 60 μL elution volume (Atrida, Amersfoort, The Netherlands), following the manufacturer’s instructions. Isolate sequencing libraries were created using Nextera XT DNA library preparation (Illumina, San Diego, CA, USA) according to the manufacturer’s instructions, and subsequently underwent Illumina sequencing using the MiSeq V3 chemistry (Illumina, San Diego, CA, USA) for the production of 2 × 250 bp paired-end reads. All sequencing data have been submitted to the Sequence Read Archive (SRA) [31] under BioProject PRJNA698782. Individual accession numbers are provided in Supplementary Table S1. This dataset was extended with data from previous studies, for *S. flexneri* with WGS data from Connor et al. (*n* = 155) [10], and for *S. sonnei* with WGS data from Baker et al. (*n* = 3) [32], Holt et al. (*n* = 118) [11], and The et al. (*n* = 73) [24]. Isolates sequenced in the context of this study are referred to as the ‘Belgian’ isolates, while isolates from previous studies are referred to as the ‘background collection’ (which did not contain any samples from Belgium). The available metadata for the background collections only indicated if isolates were resistant or not to ciprofloxacin, and the isolates were therefore considered high-level resistant since the distinction between low- and high-level resistance could not be made.

### 2.3. Isolate WGS Characterization

Reads were trimmed and *de novo* assembled as described previously [33]. A local installation of PointFinder [26] (checked out from BitBucket on 27th of February 2019) was used to detect mutations associated with resistance against ciprofloxacin. AMR genes were detected using the sequences from the ResFinder [28] database (retrieved on 3rd of January 2021), as described previously [33]. Subsequent analysis was limited specifically to genes and mutations associated with resistance to ciprofloxacin, genes and mutations associated with other antibiotics were not considered for this study but are available in the supplementary information (Supplementary Tables S6 and S7). Detected genes were predicted to be either plasmid- or chromosome-located by aligning their corresponding contigs against the NCBI *nt* database (downloaded on 7 October 2019) using blastn 2.7.0 with the ‘-task’ parameter set to ‘megablast’ [34]. Genes were predicted as plasmid-located when at least 50% of the best hits according to their bit-score contained the term ‘plasmid’ in the header. The predicted genotypic susceptibility was compared to the observed phenotypic resistance to ciprofloxacin. Serotypes for *S. flexneri* samples were determined *in silico* using a WGS-based workflow described previously [21]. Serotyping is not performed for *S. sonnei* and was therefore not evaluated. Core genome multi-locus sequence typing (cgMLST) was performed using the EnteroBase *E. coli* scheme [35] (retrieved on 13th of December 2020) as described previously [33]. Only samples with ≥90% of cgMLST alleles identified were retained in the study [36].

### 2.4. Phylogenomic Analysis

Relatedness between isolates was determined by constructing phylogenies based on cgMLST results. Allele matrices for *S. flexneri* and *S. sonnei* were constructed separately. Minimum spanning trees (MSTs) were constructed from the filtered allele matrices using GrapeTree 2.2 [37] with the ‘method’ option set to ‘MSTreeV2′. Phylogenies were visualized and annotated in IToL [38]. Clusters of related isolates were constructed by collapsing branches with 50 or fewer allele differences. Lineages and PGs for the Belgian isolates were derived based on the clustering with annotated isolates from the background collection, for which the PG or lineage was known. Single-nucleotide polymorphism (SNP) addresses were determined for isolates assigned to the most common PG for *S. flexneri* and *S. sonnei* lineages using PHEnix v1.4.1 (https://github.com/phe-bioinformatics/PHEnix, accessed on 3 September 2019) and SnapperDB 1.0.6 [39] setting the average depth cutoff to 30x, as described previously [40]. The SNP-address is a strain level 7-digit nomenclature based on the number of pair-wise SNP differences. Each digit represents the cluster membership for the given number of SNP differences, starting (right to left) with 0 (e.g., no SNP differences) to 5, 10, 25, 50, 100, and 250. Isolates sharing the same cluster digit differ by fewer than the corresponding number of SNPs [41]. The selected reference genomes extracted from NCBI used for read mapping were the representative genomes for both species: 2a str. 301 (accession NC_004337.2) for *S. flexneri* and 53G (accession HE616528.1) for *S. sonnei*.

## 3. Results

### 3.1. Antimicrobial Resistance to Ciprofloxacin

Of the 2315 *Shigella* isolates received by the Belgian NRC between January 2013 and December 2018, 1690 (73.0%) were identified as *S. sonnei*, 485 (21.0%) as *S. flexneri*, 96 (4.1%) as *S. boydii*, 33 (1.4%) as *S. dysenteriae* and 11 isolates were untypeable. Similarly to observations in other countries [42], the occurrence of high-level ciprofloxacin-resistant isolates increased, from 0.9% in 2006 to 32.3% in 2018 (Figure 1). Additionally, 8.4% of received strains were low-level resistant to ciprofloxacin.

### 3.2. Isolate WGS Characterization

**Figure 2:**
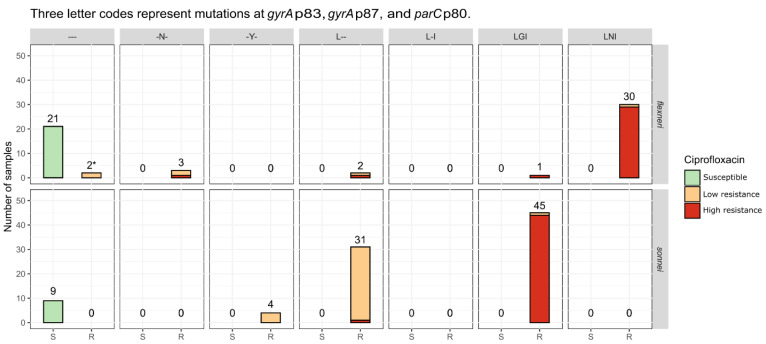
Occurrence of *gyrA* p83, *gyrA* p87 and *parC* p80 mutations associated with ciprofloxacin resistance. The *X*-axis on each subplot represents the sensitive and resistant samples, the *Y*-axis represents the number of isolates. Bars are colored according to observed phenotypic resistance against ciprofloxacin. The top and bottom rows show results for *S. flexneri* and *S. sonnei*, respectively. Columns represent the patterns of amino-acid mutations in the *gyrA* p83, *gyrA* p87 and *parC* p80 positions (in that order). Dashes indicate that the wild type amino acid was observed at the corresponding position. Mutations detected in isolates without phenotypic testing data for ciprofloxacin resistance were omitted. * The two low-level ciprofloxacin-resistant samples with wild-type amino-acids at the *gyrA* p83, *gyrA* p87, and *parC* p80 positions (i.e., ‘---’ pattern) both contained the *qnrS1* gene, potentially explaining the resistance. Abbreviations: susceptible (S); resistant (R).

Read trimming and assembly statistics for the sequenced isolates are listed in Supplementary Tables S2 and S3, respectively. The median total assembly length of the Belgian isolates was 4,466,264 bp for *S. flexneri* and 4,553,436 bp for *S. sonnei*, with N50 values of 32,373 and 25,389, respectively. Sequencing depth was determined by mapping processed reads against the assembled contigs and varied between 9-73x with a median of 38x for the Belgian *S. flexneri* isolates, and between 9-86x with a median of 34x for the Belgian *S. sonnei* isolates. The percentage of cgMLST alleles identified for each isolate is listed in Supplementary Table S4. Three *S. flexneri* and two *S. sonnei* Belgian isolates were removed because less than 90% of cgMLST loci were identified, resulting in 59 and 89 retained isolates, respectively. For the background collection, 26 isolates were removed for *S. flexneri* and four for *S. sonnei* by enforcing a 90% cgMLST loci identified cutoff, resulting in 129 and 190 retained *S. flexneri* and *S. sonnei* background collection isolates, respectively. Only retained isolates were used for subsequent analyses and calculation of composite metrics (e.g., occurrence of ciprofloxacin resistance mutations). In silico determined serotypes for the *S. flexneri* isolates are listed in Supplementary Table S5. Overviews of the detected AMR genes and point mutations are provided in Supplementary Tables S6 and S7, respectively (including antibiotics other than ciprofloxacin). The most commonly observed mutations associated with ciprofloxacin resistance were located at *gyrA* p83, *gyrA* p87, and *parC* p80, as illustrated in Figure 2 for the Belgian isolates and Supplementary Figure S1 for the background collection. Additionally, the more rare *parE* S458A mutation [43] was observed in two Belgian *S. flexneri* isolates (S17BD04752 and S18BD00006), but not in any of the isolates from the background collection. Quinolone resistance genes were observed less frequently than point mutations (Supplementary Tables S7 and S8). For both species, all detected *qnr* genes were predicted to be plasmid-encoded (Supplementary Table S9). Five Belgian *S. flexneri* isolates carried the *qnrS1* gene, of which three exhibited low-level and two high-level resistance to ciprofloxacin. Of 23 Belgian *S. flexneri* isolates with the wild type allele at the aforementioned *gyrA* and *parC* positions, 21 were susceptible to ciprofloxacin. The two resistant isolates carried the *qnrS1* gene and exhibited low-level resistance. The LNI pattern (with the letters representing the amino acids at *gyrA* p83, *gyrA* p87, and *parC* p80) was the most commonly observed combination of mutations in the Belgian *S. flexneri* isolates. Of the 59 Belgian isolates, 30 carried these three mutations and all of them exhibited high-level resistance to ciprofloxacin, except for isolate S16BD03881 which exhibited low-level resistance (albeit close to the threshold for high-level resistance). Other patterns observed in the Belgian *S. flexneri* isolates were: -N- (where ‘-‘ represents the wild-type amino acid) (*n* = 3), L-- (*n* = 2), and LGI (n = 1), which were all exclusively observed in isolates with resistance to ciprofloxacin. The two isolates carrying the *parE* S458A mutation contained a LNI pattern for the aforementioned mutations and exhibited high-level resistance to ciprofloxacin. For *S. sonnei*, isolates without mutations at the aforementioned three positions were 100% susceptible to ciprofloxacin, for both the Belgian isolates (*n* = 9) and the background collection (*n* = 9). The most frequently observed mutation pattern in the Belgian *S. sonnei* isolates was LGI (*n* = 45), for which almost all isolates exhibited high-level resistance to ciprofloxacin (*n* = 44) and the one remaining isolate exhibited low-level resistance. This was consistent with data from the background collection, for which all 51 isolates carrying these mutations were phenotypically resistant. Isolates carrying only a single mutation, such as ‘-Y-‘ (*n* = 4) and ‘L--‘ (*n* = 31), generally exhibited low-level resistance. No *qnr* genes were detected in the *S. sonnei* isolates from background collections, but the Belgian isolates contained *qnrS1* (*n* = 3), *qnrB4* (*n* = 2), and *qnrB19* (*n* = 1), associated with low- (*n* = 4) and high-level (*n* = 2) resistance to ciprofloxacin, although all of these isolates also carried at least a single mutation at the aforementioned positions. To summarize, for both species, all ciprofloxacin-susceptible Belgian isolates did not carry any mutations or *qnr* genes associated with ciprofloxacin resistance, while all resistant isolates carried at least one mutation or gene.

### 3.3. Phylogenomic Analysis

**Figure 3:**
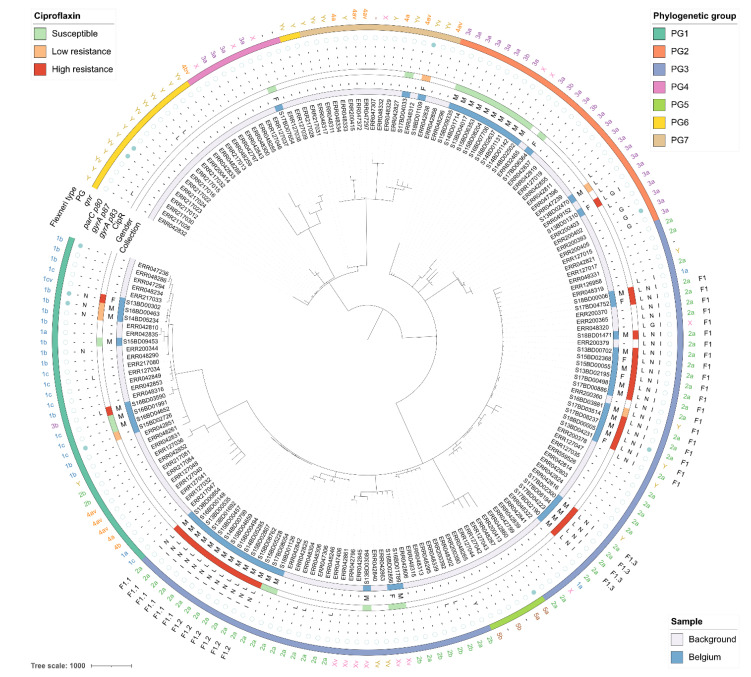
Minimum spanning tree for the *S. flexneri* samples. The scale bar is expressed as number of cgMLST allele differences between isolates. The annotations are (from inner to outer ring): isolate name, isolate origin (blue indicates the isolate was obtained from this study, white from public reference collections), patient gender (‘M’: male, ‘F’: female), phenotypically determined resistance to ciprofloxacin (green, light red, and dark red indicate susceptibility, low-level resistance, and high-level resistance, respectively; white indicates that ciprofloxacin resistance information was not available), mutations in *gyrA* p83, *gyrA* p87, and *parC* p80, presence of *qnr* genes (a filled or empty blue circle indicates presence or absence, respectively), phylogenetic group, serotype, and cluster membership. A dash (‘-’) for a *gyrA* or *parC* mutation indicates that the wild-type amino acid was observed at the corresponding position.

**Figure 4:**
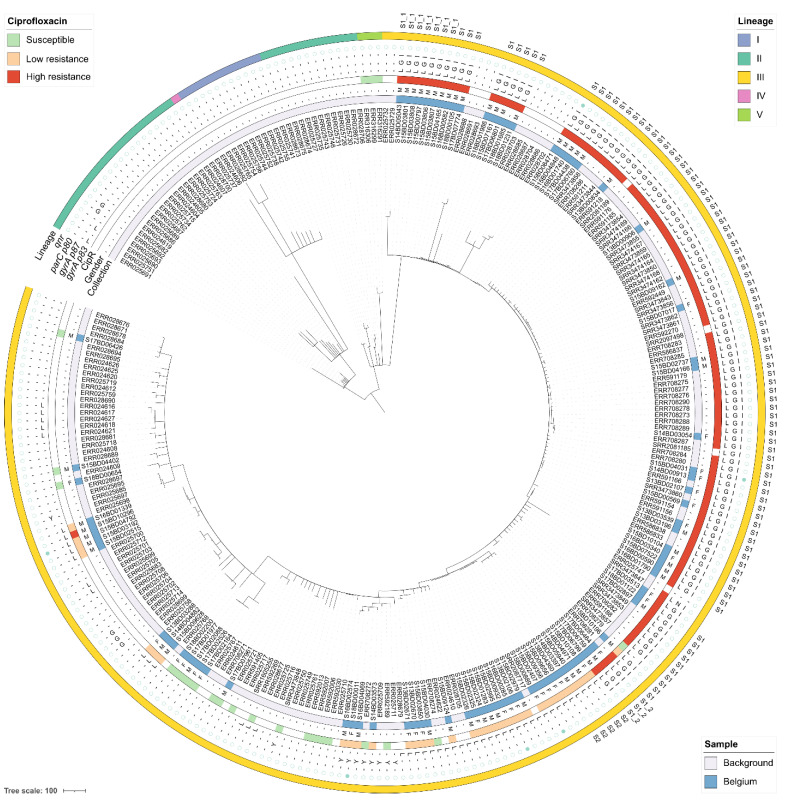
Minimum spanning tree for the *S. sonnei* isolates. The scale bar is expressed as number of cgMLST allele differences between isolates. The annotations are (from inner to outer ring): isolate name, isolate origin (blue indicates the isolate was obtained from this study, white from public reference collections), patient gender (‘M’: male, ‘F’: female), phenotypically determined resistance to ciprofloxacin (green, light red, and dark red indicate susceptibility, low-level resistance, and high-level resistance, respectively; white indicates that ciprofloxacin resistance information was not available), mutations in *gyrA* p83, *gyrA* p87, and *parC* p80, presence of *qnr* genes (a filled or empty blue circle indicates presence or absence, respectively), lineage, and cluster membership. A dash (‘-’) for a *gyrA* or *parC* mutation indicates that the wild-type amino acid was observed at the corresponding position.

The cgMLST-based phylogenies with all *S. flexneri* and *S. sonnei* isolates are shown in Figure 3 and Figure 4, respectively, and are represented as networks in Supplementary Figure S2. The 59 Belgian *S. flexneri* isolates from this study were distributed across five of the seven PGs, with none of the isolates assigned to PG5 or PG6. The most commonly observed PGs were PG3 (*n* = 35), followed by PG2 (*n* = 13), PG1 (*n* = 8), PG7 (*n* = 2), and PG4 (*n* = 1) (Supplementary Table S10). Although certain serotypes made up the majority of certain PGs (i.e., the majority of PG3 isolates were *S. flexneri* 2a), all of the PGs contained multiple serotypes, as observed elsewhere [10,44]. Less diversity was observed in the 89 Belgian *S. sonnei* isolates, which were all classified as lineage III, with 53% of isolates exhibiting resistance to ciprofloxacin. For both species, several subclades of closely related isolates were identified using cgMLST and SNP analysis, with mostly congruent results.

**Figure 5:**
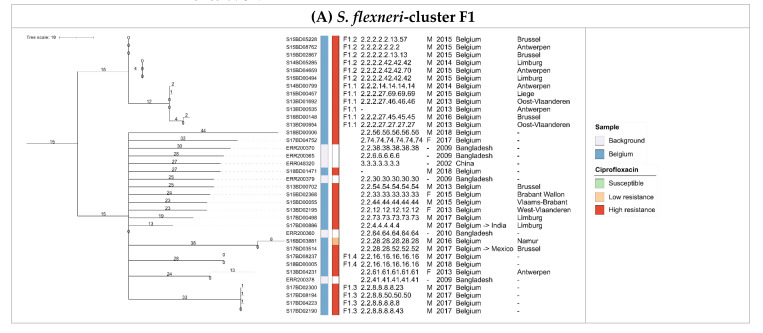

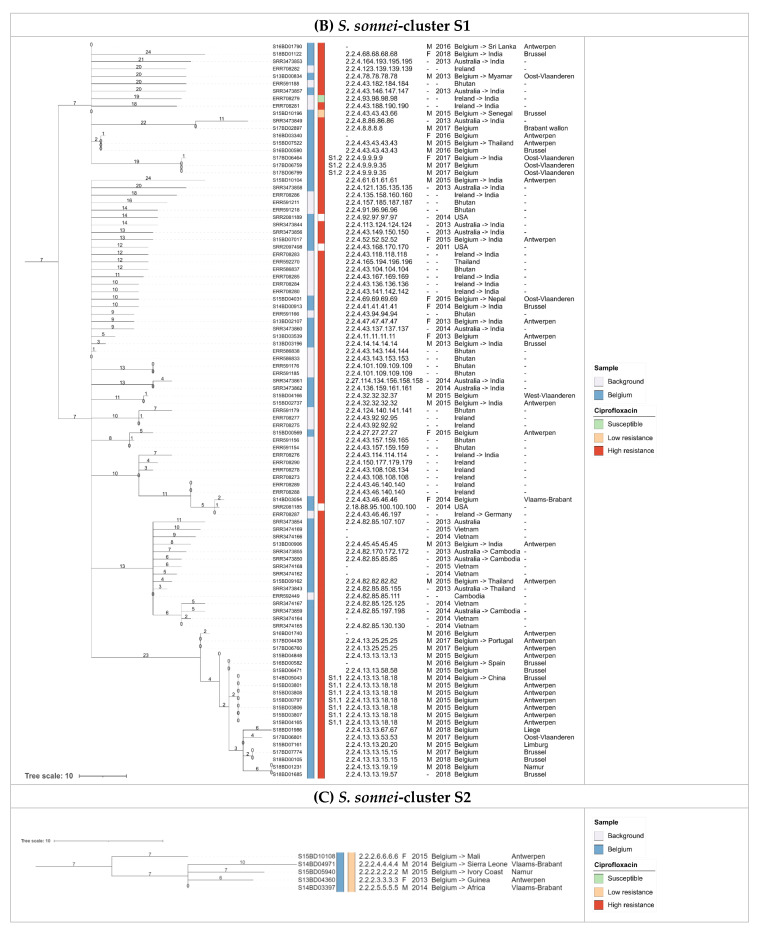
Minimum spanning trees for *S. flexneri* cluster F1 (Subplot A) and *S. sonnei* clusters S1 (Subplot B) and S2 (Subplot C). The scale bar is expressed as number of cgMLST allele differences between isolates. Annotations are (from left to right): isolate name, collection (blue: Belgium, white: background collection), ciprofloxacin resistance (green, light red, and dark red indicate susceptibility, low-level resistance, and high-level resistance, respectively; white indicates that ciprofloxacin resistance information was not available), sub-cluster, SNP address, patient gender (‘M’: male, ‘F’: female), collection year, travel and/or geographic information, and province (for the Belgian samples only). Travel information is provided as home country -> travel destination. When only a single location is listed, no travel history was recorded (and the home country is indicated). Samples without a SNP address did not pass average depth filtering.

The rate of ciprofloxacin resistance for *S. flexneri* isolates varied between groups, but four out of five PGs contained at least one ciprofloxacin-resistant isolate. Diversity and phylogenomic distances in the MST of the *S. flexneri* isolates were overall large, with ciprofloxacin resistance widespread and not limited to certain (sub)clades. The majority of Belgian isolates were assigned to a clade of 36 ciprofloxacin-resistant isolates in PG3 that was constructed by collapsing branches on 50 or fewer alleles differences. The clade is denoted as F1 in Figure 3 and detailed in Figure 5A, and also included isolates from the background collection from China and Bangladesh. Nearly all isolates in this clade contained the *gyrA* S83L and *parC* S80I mutations, and all isolates except for ERR048320 also carried the *gyrA* D87N mutation. Five Belgian isolates assigned to PG3 did not cluster together with this clade, of which three were susceptible and two exhibited high-level resistance to ciprofloxacin. Of the eight ciprofloxacin-resistant *S. flexneri* isolates not assigned to PG3, only S13BD02470 showed similarity to the background collection, differing eight alleles from an isolate collected in Vietnam in 2010. The other seven isolates did not cluster closely together with any isolates from neither the Belgian nor background collection. Within the F1 clade, four clusters of highly similar Belgian *S. flexneri* isolates were observed that differed by fewer than five cgMLST alleles, indicating a very close phylogenetic relationship (F1.1, F1.2, F1.3, and F1.4). Sub-cluster F1.1 contained six high-level ciprofloxacin-resistant isolates collected over several years exclusively from male patients in different Belgian provinces. Despite their relatively high degree of similarity, no indications of (in)direct transmission were present, since the isolates were collected quite distantly both temporally and/or geographically, and their SNP addresses indicated differences between 10 and 50 SNPs. In contrast, for sub-cluster F1.2 containing six isolates, isolate S14BD05285 was collected at the end of 2014 in the Limburg province. In 2015, an identical isolate based on both cgMLST and SNP typing was collected in the same province (S15BD00494), and later that year another isolate with an identical cgMLST profile that differed by fewer than five SNPs was collected in a neighboring province (S15BD04659), suggesting (in)direct transmission. Similar putative transmission patterns were observed for the three other isolates in sub-cluster F1.2 (5–10 SNPs difference), the four isolates from sub-cluster F1.3 (5–25 SNPs difference), and the two isolates from sub-cluster F1.4 (0 SNPs difference). None of the patients in these four sub-clusters had reported international travel, indicating the infection was likely acquired through domestic circulation.

For *S. sonnei*, the observed diversity and phylogenomic distances of Belgian isolates was much lower and nearly all high-level ciprofloxacin-resistant *S. sonnei* isolates clustered together in clade S1 (*n* = 99) with less than 25 cgMLST allele differences between isolates (Figure 4). Some Belgian isolates in this clade showed high similarity to isolates from the background collection from Asian countries. Out of 44 Belgian isolates in this clade, 18 had an indicated travel history to India (*n* = 9), Thailand (*n* = 2) or other countries (*n* = 7) (Figure 5B). The Belgian isolates from this clade were collected across five years from patients originating from all over Belgium. Within clade S1, two potential cases of travel-related imports followed by domestic circulation were observed in sub-clusters of isolates with maximum one allele difference. A first potential example is provided by sub-cluster S1.1 (*n* = 7) in Figure 4 and detailed in Figure 5B. Isolate S14BD05043 was collected from a male who travelled to China in 2014. In the following year, six isolates with identical cgMLST profiles and SNP addresses were collected from males in a neighboring province, for which none had an indicated travel history. This raises the possibility that this strain was imported from China in 2014, and spread clonally within Belgium afterwards. A second potential example is provided by sub-cluster S1.2 (*n* = 3) in Figure 4 and detailed in Figure 5B. A female travelled to India in 2017, and in the same year two highly similar isolates differing a single cgMLST allele and between 1–5 SNPs were collected in the same Belgian province from males that did not have an indicated travel history. Isolates with an indicated travel history that did not show similarity to other isolates were also observed. Five low-level ciprofloxacin-resistant strains grouped in clade S2 in Figure 4 and detailed in Figure 5C showed similarity based on cgMLST and differed 25–50 SNPs according to their SNP address. The isolates were collected between 2013 and 2015 from patients in three different provinces of Belgium, who all had travelled to West-African countries, suggesting independent travel-related import events.

## 4. Discussion

In this study, we analyzed the genomic variability among 148 *Shigella* isolates collected during the national surveillance of shigellosis in Belgium between 2013 and 2018. Phylogenomic investigation demonstrated that classification based on serological properties for *S. flexneri* did not accurately reflect genetic relationships, as indicated previously [10,44]. All seven PGs contained multiple serotypes, and serotype switching was frequently observed, even within clades of closely related isolates (Figure 3). Pathogen typing using genomics information, such as based on cgMLST and the SNP address, therefore constitutes a more suitable method for classification not impacted by sporadic serotype switching events moderated by horizontally transmissible genetic elements [45].

In recent years, an increase in resistance to ciprofloxacin was observed in the most prevalent *Shigella* species (i.e., *S. sonnei* and *S. flexneri*) (Figure 1), which was investigated here using a phylogenomic approach. Although the majority of ciprofloxacin-resistant Belgian *S. flexneri* isolates were assigned to PG3, ciprofloxacin resistance was not limited to a single PG (Figure 3). Resistance was mainly associated with the co-occurrence of the *gyrA* S83L, *gyrA* D87N, and *parC* S80I mutations (Figure 2). The *parE* S458A mutation [43,46] was also observed in two Belgian *S. flexneri* isolates, but its association with ciprofloxacin could not be determined since the corresponding isolate contained various other mutations associated with ciprofloxacin resistance. A similar pattern with the *gyrA* D87G instead of the *gyrA* D87N mutation was observed in one Belgian *S. flexneri* isolate but was more common for high-level ciprofloxacin-resistant *S. sonnei*, for which all resistant Belgian isolates were classified as lineage III (Figure 4), previously reported as being the dominant lineage [11].

In developed countries, shigellosis is known to spread through international travel and endemic transfer between patients in risk groups such as men who have sex with men (MSM) [10,47]. Ingle et al. previously reported that ciprofloxacin resistance was largely associated with international travel for both *S. flexneri* and *S. sonnei* [12]. For the Belgian *S. flexneri* isolates from this study, sporadic travel-related imports notwithstanding, the majority of infections could not be traced back to travel and were likely associated with domestically circulating strains (e.g., sub-clusters F1.1, F1.2, F1.3, and F1.4), although this does not preclude that travel-acquired imports occur more frequently than observed here since not all patients seek medical treatment and not all patient isolates are sent to the NRC. For *S. sonnei*, a pattern of travel-related high-level ciprofloxacin-resistant infections was observed for the Belgian isolates, mainly from people who travelled to Asia (cluster S1), as observed previously [24]. However, a second set of isolates of low-level ciprofloxacin-resistant *S. sonnei* infections that likely originated from West-African countries through several independent travel imports (cluster S2), was also observed [7]. These observations indicate that a substantial risk of travel-related ciprofloxacin-resistant infection exists. Moreover, travel-related events were sometimes followed by domestic circulation, as illustrated by two cases where expansion of identical or highly similar strains was observed in patients without an indicated travel history after an earlier travel-related import (sub-clusters S1.1 and S1.2). For both species, since ciprofloxacin resistance was found to be mainly associated with chromosomally-located point mutations, it appears unlikely that closely related strains in the phylogenomic topology acquired ciprofloxacin resistance independently from each other through horizontal gene transfer from mobile genetic elements. The presence of mobile *qnr* genes was only found for a limited set of samples. The high occurrence of clusters limited to male patients and the skewed gender distribution in the samples received by the NRC (73.7% male for *S. flexneri* and 60.4% male for *S. sonnei*) could indicate transmission linked to MSM, although this cannot be confirmed without epidemiological follow-up investigation.

We acknowledge the following limitations of this study. Firstly, not all infected patients are sampled and not all isolates are sent to the NRC. Additionally, WGS was only performed on a subset of all *Shigella* strains received by the NRC due to budgetary constraints. The current phylogenomic analysis does therefore only approximate the full extent of genomic variation of shigellosis within Belgium and may present a biased view on diversity within our country. Secondly, the available sample metadata (e.g., travel information) might be subject to omissions and mistakes, which is a limitation shared with most genomic studies [48,49,50]. Thirdly, as illustrated by our study, the combination of WGS-based phylogenomics and sample metadata can be used to perform hypothesis generation for understanding the dissemination and transmission patterns of shigellosis, but would however still require detailed epidemiological follow-up investigation, outside the scope of this study, to reject or confirm. Fourthly, resistance to azithromycin, which is another commonly used antibiotic to treat Shigellosis, was not analyzed in the NRC until 2018, and data on this antibiotic could therefore not be incorporated into this study.

In conclusion we have shown through in-depth phylogenomic investigation that ciprofloxacin-resistant infections among Belgian patients suffering from shigellosis likely spread through a combination of travel-related import and domestic circulation. This study illustrates the added value of genomics-based surveillance for *Shigella,* enabling accurate phenotypic AMR characterization using genomic AMR markers and more accurate clustering compared to using conventional serotyping affected by serotype-switching events. WGS also allowed investigating clusters of closely related isolates with very high resolution by using cgMLST, and even up to the single nucleotide level by using SNP-based typing.

## Data Availability

All sequencing data has been deposited in SRA under BioProject PRJNA698782. Results of molecular characterization and available metadata are provided in the Supplementary.

## Supplementary Materials

The following are available online at https://www.mdpi.com/article/10.3390/microorganisms9040767/s1, Figure S1: Occurrence of *gyrA* p83, *gyrA* p87 and *parC* p80 mutations associated with ciprofloxacin resistance in the background collection. Figure S2: Network representation of minimum spanning trees for *S. sonnei* (A) and *S. flexneri* (B), Table S1: Accession number and metadata for the Belgian samples, Table S2: Read trimming statistics, Table S3: Assembly and coverage statistics, Table S4: Percentage of cgMLST alleles identified, Table S5: In silico determined serotypes for all *S. flexneri* samples, Table S6: Detected AMR genes for all samples, Table S7: Detected AMR point mutations for all samples, Table S8: Detected *qnr* genes, Table S9: Classification of contigs carrying *qnr* genes, Table S10: Lineage and PG classification, and ciprofloxacin resistance for the Belgian samples and background collection.
