# Supplementary material for: Phylogenomic Investigation of Increasing Fluoroquinolone Resistance among Belgian Cases of Shigellosis between 2013 and 2018 Indicates Both Travel-Related Imports and Domestic Circulation
Source: Microorganisms. 2021 Apr 6;9(4):767. doi: 10.3390/microorganisms9040767 (PMC8067512; doi:10.3390/microorganisms9040767)
Supplement: Supplementary file 1 [file microorganisms-09-00767-s001.pdf]

SUPPLEMENTARY

# Ciprofloxacin resistant *Shigella* in Belgium

## Figures

**Supplementary Figure S1: Occurrence of *gyrA* p83, *gyrA* p87 and *parC* p80 mutations associated with ciprofloxacin resistance in the background collection.**

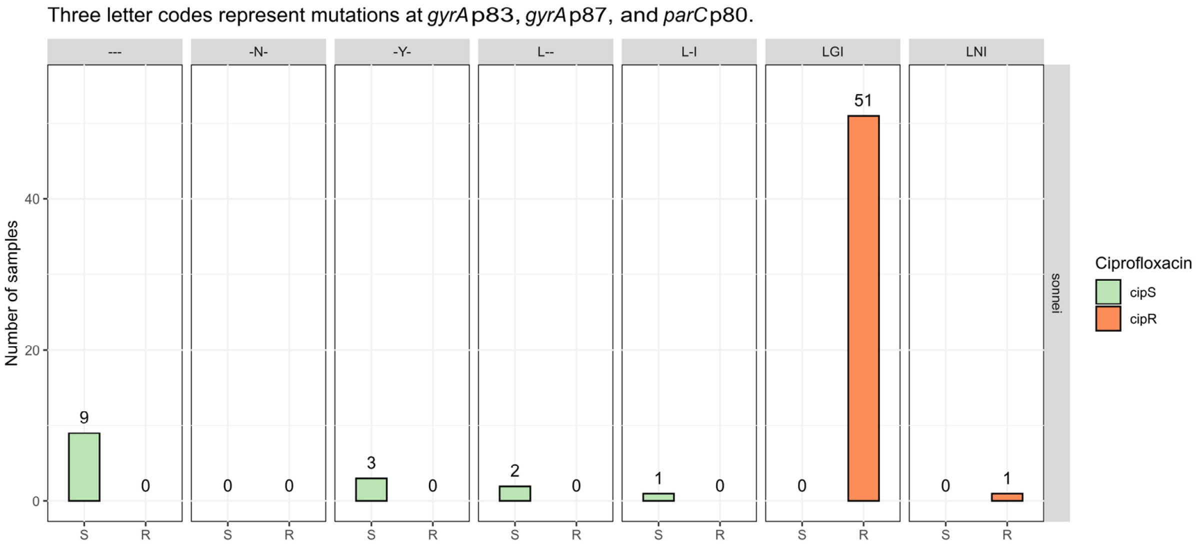


The X-axis on each subplot represents the collection of the corresponding samples, the Y-axis represents the number of isolates. Bars are colored according to observed phenotypic resistance against ciprofloxacin. No phenotypic resistance data was available for the *S. flexneri* samples from the background collection. Columns represent the patterns of amino-acid mutations in the *gyrA* p83, *gyrA* p87 and *parC* p80 positions (in that order). Dashes indicate that the *wt* amino acid was observed at the corresponding position. Mutations detected in samples without phenotypic testing data for ciprofloxacin resistance were omitted. Abbreviations: resistant (R); sensitive (S).

**Supplementary Figure S2: Network representation of minimum spanning trees for *S. sonnei* (A) and *S. flexneri* (B).**

***A) S. flexneri***

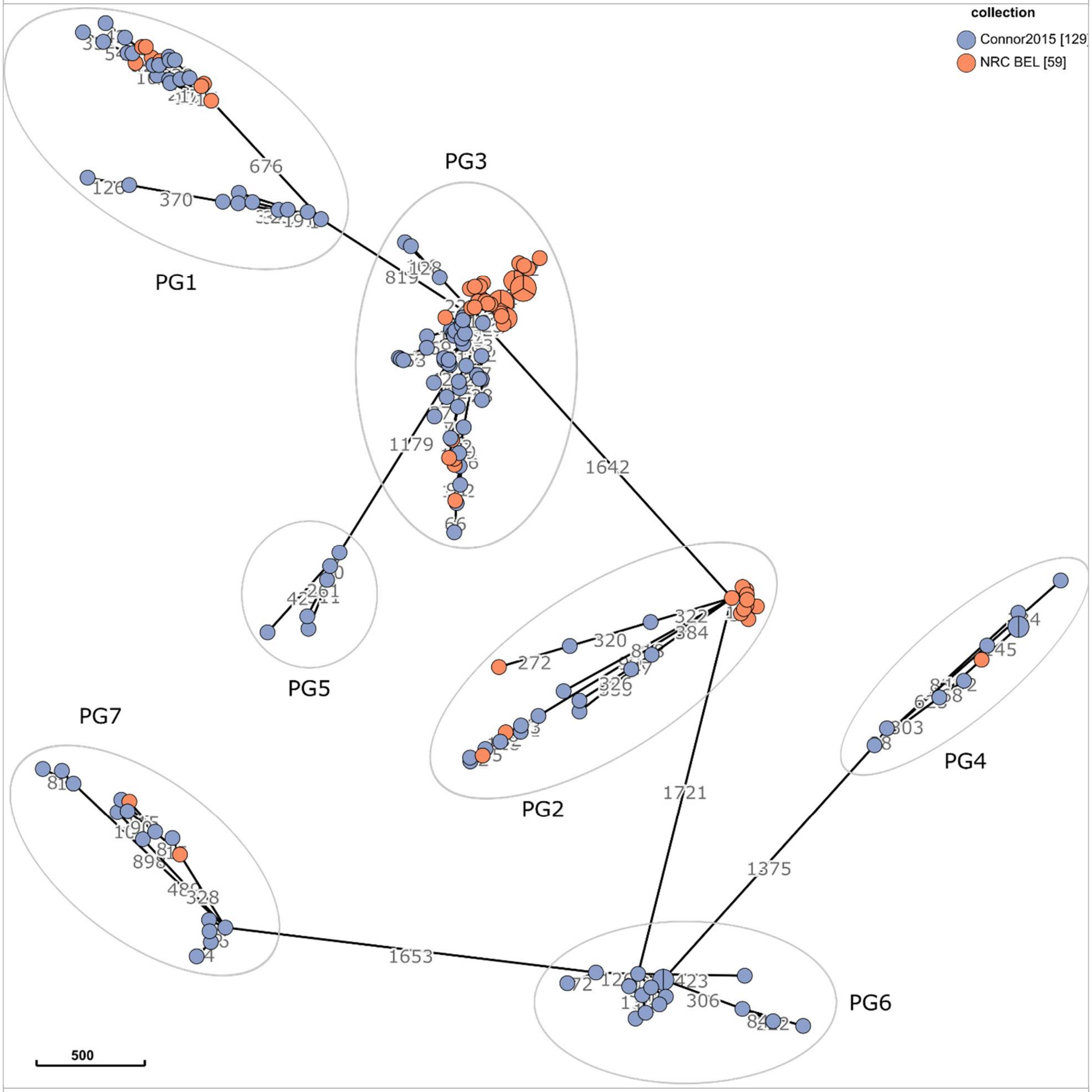


***B) S. sonnei***

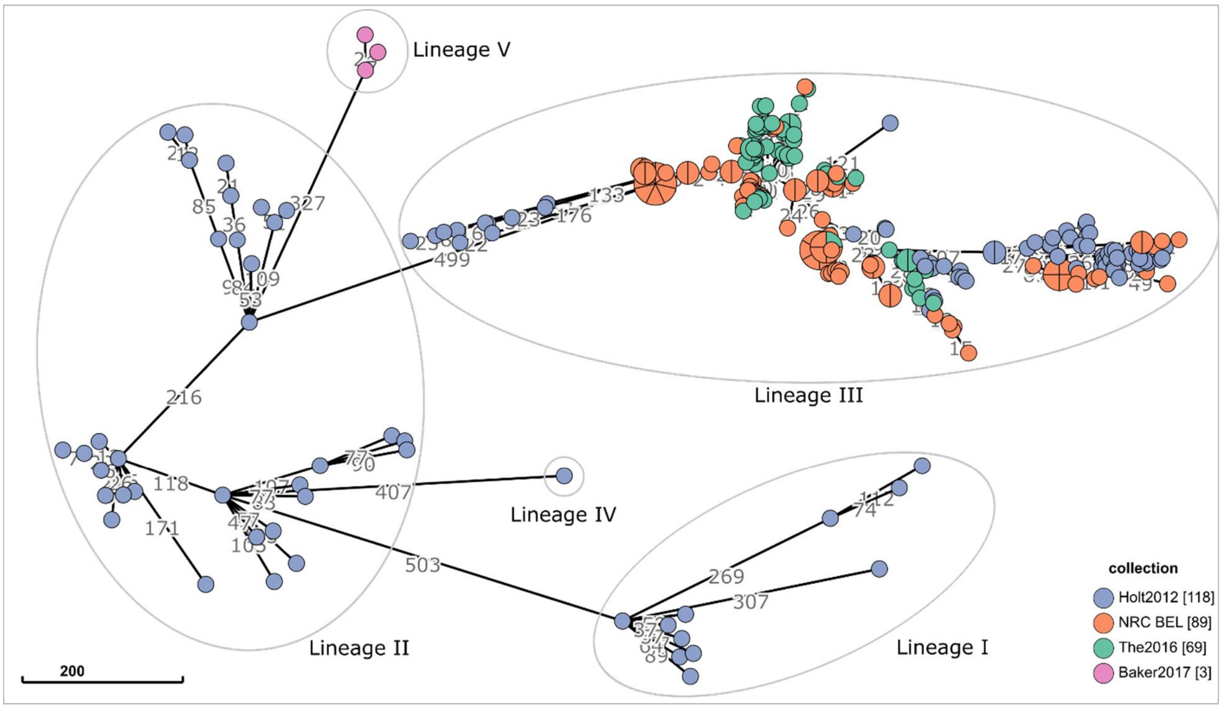


The scale bar is expressed as number of cgMLST allele differences between isolates. The size of the nodes scales with the number of isolates with identical core-genome profiles. Nodes are colored according to the name of the collection (see Material and methods), Belgian samples are indicated in red. Samples were grouped according to PG (*S. flexneri*, subplot A) or lineage (*S. sonnei*, subplot B).

## Tables

### Supplementary Table S1: Accession number and metadata for the Belgian samples.

| Sample | Species | SRA accession | Ciprofloxacin resistance | Year | Travel | Gender | Province |
|---|---|---|---|---|---|---|---|
| S13BD00302 | *S. flexneri* | SRR13608899 | High-level | 2013 | Belgium -> France | Female | Namur |
| S13BD00535 | *S. flexneri* | SRR13608898 | High-level | 2013 | Belgium | Male | Antwerpen |
| S13BD00702 | *S. flexneri* | SRR13608834 | High-level | 2013 | Belgium | Male | Brussel |
| S13BD00834 | *S. flexneri* | SRR13608812 | High-level | 2013 | Belgium -> Myamar | Male | Oost-Vlaanderen |
| S13BD00854 | *S. flexneri* | SRR13608790 | High-level | 2013 | Belgium | Male | Oost-Vlaanderen |
| S13BD00906 | *S. flexneri* | SRR13608779 | High-level | 2013 | Belgium -> India | Male | Antwerpen |
| S13BD01310 | *S. flexneri* | SRR13608897 | High-level | 2013 | Belgium | Female | West-Vlaanderen |
| S13BD01692 | *S. flexneri* | SRR13608886 | High-level | 2013 | Belgium | Male | Oost-Vlaanderen |
| S13BD02106 | *S. flexneri* | SRR13608853 | Low-level | 2013 | Belgium -> Turkey | Female | Antwerpen |
| S13BD02107 | *S. flexneri* | SRR13608836 | High-level | 2013 | Belgium -> India | Female | Antwerpen |
| S13BD02195 | *S. flexneri* | SRR13608835 | High-level | 2013 | Belgium | Female | West-Vlaanderen |
| S13BD02470 | *S. flexneri* | SRR13608832 | Low-level | 2013 | Belgium | Male | Brussel |
| S13BD02670 | *S. flexneri* | SRR13608830 | Low-level | 2013 | Belgium -> Cameroon | Female | Antwerpen |
| S13BD02671 | *S. flexneri* | SRR13608829 | Low-level | 2013 | Belgium -> Congo | Female | Brussel |
| S13BD03084 | *S. flexneri* | SRR13608828 | Susceptible | 2013 | Belgium | Male | Vlaams-Brabant |
| S13BD03196 | *S. flexneri* | SRR13608827 | High-level | 2013 | Belgium -> India | Male | Brussel |
| S13BD03288 | *S. flexneri* | SRR13608826 | Low-level | 2013 | Belgium -> Cuba | Female | Antwerpen |
| S13BD03539 | *S. flexneri* | SRR13608818 | High-level | 2013 | Belgium | Female | Antwerpen |
| S13BD04017 | *S. flexneri* | SRR13608817 | Susceptible | 2013 | Belgium | Male | Limburg |
| S13BD04231 | *S. flexneri* | SRR13608816 | High-level | 2013 | Belgium | Female | Antwerpen |
| S13BD04360 | *S. flexneri* | SRR13608815 | Low-level | 2013 | Belgium -> Guinea | Female | Antwerpen |
| S14BD00799 | *S. flexneri* | SRR13608814 | High-level | 2014 | Belgium | Male | Antwerpen |
| S14BD00913 | *S. flexneri* | SRR13608804 | High-level | 2014 | Belgium -> India | Female | Brussel |
| S14BD01131 | *S. flexneri* | SRR13608800 | Susceptible | 2014 | Belgium | Male | Limburg |
| S14BD01142 | *S. flexneri* | SRR13608798 | Susceptible | 2014 | Belgium | Male | Hainaut |
| S14BD01714 | *S. flexneri* | SRR13608783 | Susceptible | 2014 | Belgium | Male | Brussel |
| S14BD02502 | *S. flexneri* | SRR13608780 | Susceptible | 2014 | Belgium | Male | Antwerpen |
| S14BD02795 | *S. flexneri* | SRR13608777 | Low-level | 2014 | Belgium | Female | - |
| S14BD03054 | *S. flexneri* | SRR13608775 | High-level | 2014 | Belgium | Female | Vlaams-Brabant |
| S14BD03397 | *S. flexneri* | SRR13608771 | Low-level | 2014 | Belgium -> Africa | Male | Vlaams-Brabant |
| S14BD03573 | *S. flexneri* | SRR13608770 | Low-level | 2014 | Belgium | - | Antwerpen |

| | | | | | | | |
|---|---|---|---|---|---|---|---|
| S14BD04882 | *S. flexneri* | SRR13608766 | Low-level | 2014 | Belgium | Male | Antwerpen |
| S14BD04971 | *S. flexneri* | SRR13608764 | Low-level | 2014 | Belgium -> Sierra Leone | Male | Vlaams-Brabant |
| S14BD05043 | *S. flexneri* | SRR13608763 | High-level | 2014 | Belgium -> China | Male | Brussel |
| S14BD05234 | *S. flexneri* | SRR13608755 | Low-level | 2014 | Belgium | Male | Namur |
| S14BD05285 | *S. flexneri* | SRR13608753 | High-level | 2014 | Belgium | Male | Limburg |
| S15BD00055 | *S. flexneri* | SRR13608747 | High-level | 2015 | Belgium | Male | Vlaams-Brabant |
| S15BD00457 | *S. flexneri* | SRR13608895 | High-level | 2015 | Belgium | Male | Liege |
| S15BD00494 | *S. flexneri* | SRR13608892 | High-level | 2015 | Belgium | Male | Limburg |
| S15BD00569 | *S. flexneri* | SRR13608891 | High-level | 2015 | Belgium | Female | Antwerpen |
| S15BD00797 | *S. flexneri* | SRR13608889 | High-level | 2015 | Belgium | Male | Antwerpen |
| S15BD00889 | *S. flexneri* | SRR13608888 | Low-level | 2015 | Belgium | Female | Vlaams-Brabant |
| S15BD00932 | *S. flexneri* | SRR13608885 | Low-level | 2015 | Belgium | Female | Hainaut |
| S15BD01283 | *S. flexneri* | SRR13608884 | Low-level | 2015 | Belgium | Male | Hainaut |
| S15BD02324 | *S. flexneri* | SRR13608883 | Low-level | 2015 | Belgium | Male | Brabant wallon |
| S15BD02325 | *S. flexneri* | SRR13608882 | Low-level | 2015 | Belgium | Female | Brabant wallon |
| S15BD02326 | *S. flexneri* | SRR13608878 | Low-level | 2015 | Belgium | Male | Hainaut |
| S15BD02368 | *S. flexneri* | SRR13608877 | High-level | 2015 | Belgium | Female | Brabant wallon |
| S15BD02407 | *S. flexneri* | SRR13608872 | Low-level | 2015 | Belgium | Female | Hainaut |
| S15BD02515 | *S. flexneri* | SRR13608871 | Low-level | 2015 | Belgium | Male | Antwerpen |
| S15BD02726 | *S. flexneri* | SRR13608870 | Low-level | 2015 | Belgium | - | - |
| S15BD02737 | *S. flexneri* | SRR13608868 | High-level | 2015 | Belgium -> India | Male | Antwerpen |
| S15BD02867 | *S. flexneri* | SRR13608867 | High-level | 2015 | Belgium | Male | Brussel |
| S15BD02979 | *S. flexneri* | SRR13608859 | Low-level | 2015 | Belgium -> Dom. Rep. | Male | Antwerpen |
| S15BD02995 | *S. flexneri* | SRR13608856 | Low-level | 2015 | Belgium | Male | - |
| S15BD03192 | *S. flexneri* | SRR13608855 | Low-level | 2015 | Belgium | Male | Limburg |
| S15BD03801 | *S. flexneri* | SRR13608854 | High-level | 2015 | Belgium | Male | Antwerpen |
| S15BD03806 | *S. flexneri* | SRR13608852 | High-level | 2015 | Belgium | Male | Antwerpen |
| S15BD03807 | *S. flexneri* | SRR13608851 | High-level | 2015 | Belgium | Male | Antwerpen |
| S15BD03808 | *S. flexneri* | SRR13608847 | High-level | 2015 | Belgium | Male | Antwerpen |
| S15BD03850 | *S. flexneri* | SRR13608845 | Low-level | 2015 | Belgium | Female | Vlaams-Brabant |
| S15BD04030 | *S. flexneri* | SRR13608843 | Low-level | 2015 | Belgium | Male | Oost-Vlaanderen |
| S15BD04031 | *S. sonnei* | SRR13608823 | High-level | 2015 | Belgium -> Nepal | Female | Oost-Vlaanderen |
| S15BD04165 | *S. sonnei* | SRR13608801 | High-level | 2015 | Belgium | Male | Antwerpen |
| S15BD04166 | *S. sonnei* | SRR13608768 | High-level | 2015 | Belgium | Male | West-Vlaanderen |
| S15BD04402 | *S. sonnei* | SRR13608757 | Susceptible | 2015 | Belgium -> Ghana | Male | - |
| S15BD04659 | *S. sonnei* | SRR13608875 | High-level | 2015 | Belgium | Male | Antwerpen |
| S15BD04792 | *S. sonnei* | SRR13608864 | Low-level | 2015 | Belgium | Male | Brabant wallon |
| S15BD04848 | *S. sonnei* | SRR13608842 | High-level | 2015 | Belgium | Male | Antwerpen |
| S15BD05228 | *S. sonnei* | SRR13608838 | High-level | 2015 | Belgium | Male | Brussel |

| | | | | | | | |
|---|---|---|---|---|---|---|---|
| S15BD05940 | *S. sonnei* | SRR13608837 | Low-level | 2015 | Belgium -> Ivory Coast | Male | Namur |
| S15BD06353 | *S. sonnei* | SRR13608833 | Susceptible | 2015 | Belgium | Male | Antwerpen |
| S15BD06471 | *S. sonnei* | SRR13608831 | High-level | 2015 | Belgium | Male | Brussel |
| S15BD06515 | *S. sonnei* | SRR13608825 | Low-level | 2015 | Belgium | Male | - |
| S15BD07017 | *S. sonnei* | SRR13608824 | High-level | 2015 | Belgium -> India | Female | Antwerpen |
| S15BD07161 | *S. sonnei* | SRR13608822 | High-level | 2015 | Belgium | Male | Limburg |
| S15BD07522 | *S. sonnei* | SRR13608821 | High-level | 2015 | Belgium -> Thailand | Male | Antwerpen |
| S15BD08204 | *S. sonnei* | SRR13608820 | Susceptible | 2015 | Belgium | Male | Brussel |
| S15BD08762 | *S. sonnei* | SRR13608819 | High-level | 2015 | Belgium | Male | Antwerpen |
| S15BD09124 | *S. sonnei* | SRR13608813 | Low-level | 2015 | Belgium -> Uzbekistan | Male | Oost-Vlaanderen |
| S15BD09162 | *S. sonnei* | SRR13608811 | High-level | 2015 | Belgium -> Thailand | Male | Antwerpen |
| S15BD09335 | *S. sonnei* | SRR13608810 | Susceptible | 2015 | Belgium | Male | West-Vlaanderen |
| S15BD09356 | *S. sonnei* | SRR13608809 | Low-level | 2015 | Belgium | Female | Antwerpen |
| S15BD09453 | *S. sonnei* | SRR13608808 | Susceptible | 2015 | Belgium | Male | West-Vlaanderen |
| S15BD09529 | *S. sonnei* | SRR13608807 | High-level | 2015 | Belgium | Male | Hainaut |
| S15BD09628 | *S. sonnei* | SRR13608806 | Low-level | 2015 | Belgium -> France | Male | - |
| S15BD09656 | *S. sonnei* | SRR13608805 | Low-level | 2015 | Belgium | Female | Limburg |
| S15BD10104 | *S. sonnei* | SRR13608803 | High-level | 2015 | Belgium -> India | Male | Antwerpen |
| S15BD10108 | *S. sonnei* | SRR13608802 | Low-level | 2015 | Belgium -> Mali | Female | Antwerpen |
| S15BD10196 | *S. sonnei* | SRR13608799 | Low-level | 2015 | Belgium -> Senegal | Male | Brussel |
| S15BD10296 | *S. sonnei* | SRR13608797 | High-level | 2015 | Belgium | Male | West-Vlaanderen |
| S16BD00148 | *S. sonnei* | SRR13608796 | High-level | 2016 | Belgium | Male | Brussel |
| S16BD00285 | *S. sonnei* | SRR13608795 | Low-level | 2016 | Belgium | Female | Brabant wallon |
| S16BD00463 | *S. sonnei* | SRR13608794 | Low-level | 2016 | Belgium | Male | Brussel |
| S16BD00582 | *S. sonnei* | SRR13608793 | High-level | 2016 | Belgium -> Spain | Male | Brussel |
| S16BD00590 | *S. sonnei* | SRR13608792 | High-level | 2016 | Belgium | Male | Brussel |
| S16BD00864 | *S. sonnei* | SRR13608791 | Low-level | 2016 | Belgium | Male | - |
| S16BD01339 | *S. sonnei* | SRR13608789 | Low-level | 2016 | Belgium | Male | Namur |
| S16BD01740 | *S. sonnei* | SRR13608788 | High-level | 2016 | Belgium | Male | Antwerpen |
| S16BD01789 | *S. sonnei* | SRR13608787 | Susceptible | 2016 | Belgium -> Burkina Faso | Male | Brussel |
| S16BD01790 | *S. sonnei* | SRR13608786 | High-level | 2016 | Belgium -> Sri Lanka | Male | Antwerpen |
| S16BD01991 | *S. sonnei* | SRR13608785 | Susceptible | 2016 | Belgium | Male | Vlaams-Brabant |
| S16BD02550 | *S. sonnei* | SRR13608784 | High-level | 2016 | Belgium | Female | - |
| S16BD02574 | *S. sonnei* | SRR13608782 | Low-level | 2016 | Belgium -> Pakistan | Male | Antwerpen |
| S16BD02637 | *S. sonnei* | SRR13608781 | Susceptible | 2016 | Belgium | Male | Antwerpen |
| S16BD02856 | *S. sonnei* | SRR13608778 | Susceptible | 2016 | Belgium | Female | Liege |
| S16BD03340 | *S. sonnei* | SRR13608776 | High-level | 2016 | Belgium | Female | Antwerpen |

| | | | | | | | |
|---|---|---|---|---|---|---|---|
| S16BD03590 | *S. sonnei* | SRR13608774 | High-level | 2016 | Belgium | Male | Oost-Vlaanderen |
| S16BD03881 | *S. sonnei* | SRR13608773 | Low-level | 2016 | Belgium | Male | Namur |
| S16BD04069 | *S. sonnei* | SRR13608772 | Low-level | 2016 | Belgium | Male | - |
| S16BD04652 | *S. sonnei* | SRR13608769 | Susceptible | 2016 | Belgium | Male | Antwerpen |
| S16BD07706 | *S. sonnei* | SRR13608767 | Susceptible | 2016 | Belgium | Male | Antwerpen |
| S17BD00498 | *S. sonnei* | SRR13608765 | High-level | 2017 | Belgium | Male | Limburg |
| S17BD00886 | *S. sonnei* | SRR13608762 | High-level | 2017 | Belgium -> India | Male | Limburg |
| S17BD01977 | *S. sonnei* | SRR13608761 | Susceptible | 2017 | Belgium | Female | Vlaams-Brabant |
| S17BD02088 | *S. sonnei* | SRR13608760 | Susceptible | 2017 | Belgium -> Congo | Male | Vlaams-Brabant |
| S17BD02164 | *S. sonnei* | SRR13608759 | Susceptible | 2017 | Belgium | Female | Vlaams-Brabant |
| S17BD02190 | *S. sonnei* | SRR13608758 | High-level | 2017 | Belgium | Male | - |
| S17BD02300 | *S. sonnei* | SRR13608756 | High-level | 2017 | Belgium | Male | - |
| S17BD02526 | *S. sonnei* | SRR13608754 | Susceptible | 2017 | Belgium | Female | Vlaams-Brabant |
| S17BD02897 | *S. sonnei* | SRR13608752 | High-level | 2017 | Belgium | Male | Brabant wallon |
| S17BD03513 | *S. sonnei* | SRR13608751 | High-level | 2017 | Belgium -> India | Female | Antwerpen |
| S17BD03514 | *S. sonnei* | SRR13608750 | High-level | 2017 | Belgium -> Mexico | Male | Brussel |
| S17BD04033 | *S. sonnei* | SRR13608749 | Susceptible | 2017 | Belgium | - | - |
| S17BD04223 | *S. sonnei* | SRR13608748 | High-level | 2017 | Belgium | Male | - |
| S17BD04438 | *S. sonnei* | SRR13608896 | High-level | 2017 | Belgium -> Portugal | Male | Antwerpen |
| S17BD04752 | *S. sonnei* | SRR13608894 | High-level | 2017 | Belgium | Female | - |
| S17BD06364 | *S. sonnei* | SRR13608893 | Susceptible | 2017 | Belgium | Female | Antwerpen |
| S17BD06426 | *S. sonnei* | SRR13608890 | Susceptible | 2017 | Belgium -> Egypt | Male | Liege |
| S17BD06464 | *S. sonnei* | SRR13608887 | High-level | 2017 | Belgium -> India | Female | Oost-Vlaanderen |
| S17BD06759 | *S. sonnei* | SRR13608881 | High-level | 2017 | Belgium | Male | Oost-Vlaanderen |
| S17BD06760 | *S. sonnei* | SRR13608880 | High-level | 2017 | Belgium | Male | Antwerpen |
| S17BD06799 | *S. sonnei* | SRR13608879 | High-level | 2017 | Belgium | Male | Oost-Vlaanderen |
| S17BD06801 | *S. sonnei* | SRR13608876 | High-level | 2017 | Belgium | Male | Oost-Vlaanderen |
| S17BD07654 | *S. sonnei* | SRR13608874 | Susceptible | 2017 | Belgium | Female | Liege |
| S17BD07774 | *S. sonnei* | SRR13608873 | High-level | 2017 | Belgium | Male | Brussel |
| S17BD07961 | *S. sonnei* | SRR13608869 | Susceptible | 2017 | Belgium | Male | Vlaams-Brabant |
| S17BD08054 | *S. sonnei* | SRR13608866 | Susceptible | 2017 | Belgium | Male | - |
| S17BD08194 | *S. sonnei* | SRR13608865 | High-level | 2017 | Belgium | Male | - |
| S17BD08237 | *S. sonnei* | SRR13608863 | High-level | 2017 | Belgium | Male | - |
| S18BD00005 | *S. sonnei* | SRR13608862 | High-level | 2018 | Belgium | Male | - |
| S18BD00006 | *S. sonnei* | SRR13608861 | High-level | 2018 | Belgium | Male | - |
| S18BD00011 | *S. sonnei* | SRR13608860 | Low-level | 2018 | Belgium | Female | Hainaut |
| S18BD00105 | *S. sonnei* | SRR13608858 | High-level | 2018 | Belgium | Male | Brussel |
| S18BD00654 | *S. sonnei* | SRR13608857 | Susceptible | 2018 | Belgium | Female | Oost-Vlaanderen |
| S18BD01109 | *S. sonnei* | SRR13608850 | Low-level | 2018 | Belgium | Female | Liege |

| | | | | | | | |
|---|---|---|---|---|---|---|---|
| S18BD01122 | *S. sonnei* | SRR13608849 | High-level | 2018 | Belgium -> India | Female | Brussel |
| S18BD01126 | *S. sonnei* | SRR13608848 | Susceptible | 2018 | Belgium | Male | - |
| S18BD01231 | *S. sonnei* | SRR13608846 | High-level | 2018 | Belgium | Male | Namur |
| S18BD01471 | *S. sonnei* | SRR13608844 | High-level | 2018 | Belgium | Male | - |
| S18BD01685 | *S. sonnei* | SRR13608841 | High-level | 2018 | Belgium | - | Brussel |
| S18BD01986 | *S. sonnei* | SRR13608840 | High-level | 2018 | Belgium | Male | Liege |
| S18BD02200 | *S. sonnei* | SRR13608839 | Susceptible | 2018 | Belgium | Female | Antwerpen |

The first and second columns list the sample name and species, respectively. The third column lists the SRA accession number for the raw data. The fourth, fifth, sixth, seventh and eight columns lists the phenotypic resistance to ciprofloxacin, collection year, travel information, patient gender, and province of residence of the patient, respectively. Dashes ('-') indicate the corresponding information was not available or not collected.

**Supplementary Table S2: Read trimming statistics.**

| Sample | Species | Collection | Read pairs | Both surviving | Forward only surviving | Reverse only surviving | Both dropped |
|---|---|---|---|---|---|---|---|
| ERR042796 | *S. flexneri* | Connor2015 | 3,232,293 | 2,899,309 | 190,634 | 90,627 | 51,723 |
| ERR042797 | *S. flexneri* | Connor2015 | 3,303,958 | 2,947,324 | 212,686 | 90,166 | 53,782 |
| ERR042799 | *S. flexneri* | Connor2015 | 4,411,984 | 3,933,266 | 277,328 | 123,529 | 77,861 |
| ERR042803 | *S. flexneri* | Connor2015 | 4,495,532 | 4,012,834 | 274,202 | 128,841 | 79,655 |
| ERR042806 | *S. flexneri* | Connor2015 | 3,460,951 | 3,095,831 | 207,553 | 97,488 | 60,079 |
| ERR042810 | *S. flexneri* | Connor2015 | 13,111,321 | 11,694,912 | 814,312 | 368,893 | 233,204 |
| ERR042811 | *S. flexneri* | Connor2015 | 3,648,419 | 3,250,090 | 232,652 | 102,169 | 63,508 |
| ERR042814 | *S. flexneri* | Connor2015 | 3,771,675 | 3,375,918 | 225,777 | 105,556 | 64,424 |
| ERR042816 | *S. flexneri* | Connor2015 | 3,514,663 | 3,130,783 | 222,652 | 98,142 | 63,086 |
| ERR042819 | *S. flexneri* | Connor2015 | 3,541,201 | 3,169,179 | 213,687 | 98,030 | 60,305 |
| ERR042821 | *S. flexneri* | Connor2015 | 3,455,674 | 3,079,675 | 213,270 | 100,893 | 61,836 |
| ERR042824 | *S. flexneri* | Connor2015 | 3,321,637 | 2,961,649 | 205,874 | 94,855 | 59,259 |
| ERR042825 | *S. flexneri* | Connor2015 | 3,539,310 | 3,151,162 | 220,761 | 102,754 | 64,633 |
| ERR042827 | *S. flexneri* | Connor2015 | 4,063,283 | 3,618,156 | 255,228 | 116,394 | 73,505 |
| ERR042831 | *S. flexneri* | Connor2015 | 4,046,918 | 3,603,446 | 256,970 | 112,937 | 73,565 |
| ERR042832 | *S. flexneri* | Connor2015 | 5,478,823 | 4,891,518 | 335,883 | 155,493 | 95,929 |
| ERR042833 | *S. flexneri* | Connor2015 | 5,347,871 | 4,771,231 | 326,069 | 154,811 | 95,760 |
| ERR042835 | *S. flexneri* | Connor2015 | 3,880,472 | 3,453,915 | 240,205 | 115,774 | 70,578 |
| ERR042837 | *S. flexneri* | Connor2015 | 4,059,612 | 3,627,434 | 245,942 | 114,187 | 72,049 |
| ERR042838 | *S. flexneri* | Connor2015 | 4,354,154 | 3,882,723 | 267,224 | 126,227 | 77,980 |
| ERR042839 | *S. flexneri* | Connor2015 | 4,175,699 | 3,732,612 | 254,371 | 116,730 | 71,986 |
| ERR042840 | *S. flexneri* | Connor2015 | 4,205,399 | 3,744,968 | 263,274 | 121,672 | 75,485 |
| ERR042841 | *S. flexneri* | Connor2015 | 4,695,113 | 4,178,963 | 299,772 | 132,207 | 84,171 |
| ERR042842 | *S. flexneri* | Connor2015 | 6,297,711 | 5,618,718 | 384,708 | 180,992 | 113,293 |
| ERR042843 | *S. flexneri* | Connor2015 | 4,265,514 | 3,791,264 | 271,517 | 121,797 | 80,936 |
| ERR042845 | *S. flexneri* | Connor2015 | 4,548,630 | 4,055,490 | 279,909 | 130,241 | 82,990 |
| ERR042849 | *S. flexneri* | Connor2015 | 4,055,582 | 3,599,718 | 265,553 | 114,812 | 75,499 |
| ERR042851 | *S. flexneri* | Connor2015 | 4,003,348 | 3,557,848 | 258,245 | 112,249 | 75,006 |
| ERR042852 | *S. flexneri* | Connor2015 | 3,758,574 | 3,320,545 | 266,390 | 102,713 | 68,926 |
| ERR042853 | *S. flexneri* | Connor2015 | 4,795,265 | 4,250,157 | 320,003 | 133,613 | 91,492 |
| ERR042855 | *S. flexneri* | Connor2015 | 3,955,558 | 3,516,010 | 256,386 | 110,264 | 72,898 |
| ERR042858 | *S. flexneri* | Connor2015 | 4,171,369 | 3,695,303 | 278,194 | 119,104 | 78,768 |
| ERR042860 | *S. flexneri* | Connor2015 | 3,885,967 | 3,452,307 | 251,439 | 109,764 | 72,457 |
| ERR042861 | *S. flexneri* | Connor2015 | 3,588,095 | 3,195,994 | 226,091 | 100,784 | 65,226 |
| ERR042863 | *S. flexneri* | Connor2015 | 3,468,989 | 3,071,305 | 237,492 | 95,746 | 64,446 |
| ERR047236 | *S. flexneri* | Connor2015 | 5,590,046 | 4,931,824 | 369,648 | 174,933 | 113,641 |
| ERR047239 | *S. flexneri* | Connor2015 | 4,894,791 | 4,315,478 | 333,423 | 142,534 | 103,356 |
| ERR047294 | *S. flexneri* | Connor2015 | 5,343,621 | 4,491,228 | 414,491 | 270,685 | 167,217 |
| ERR047297 | *S. flexneri* | Connor2015 | 5,265,033 | 4,427,216 | 410,255 | 262,761 | 164,801 |
| ERR047306 | *S. flexneri* | Connor2015 | 2,626,196 | 2,206,426 | 211,199 | 126,068 | 82,503 |
| ERR047307 | *S. flexneri* | Connor2015 | 6,836,534 | 5,735,739 | 546,972 | 335,763 | 218,060 |
| ERR047372 | *S. flexneri* | Connor2015 | 6,151,624 | 4,551,603 | 877,506 | 348,083 | 374,432 |
| ERR047396 | *S. flexneri* | Connor2015 | 9,925,315 | 7,161,340 | 1,571,853 | 521,260 | 670,862 |
| ERR047406 | *S. flexneri* | Connor2015 | 2,172,757 | 1,727,276 | 243,125 | 107,415 | 94,941 |
| ERR048234 | *S. flexneri* | Connor2015 | 5,080,509 | 4,634,860 | 202,201 | 163,326 | 80,122 |
| ERR048246 | *S. flexneri* | Connor2015 | 6,064,789 | 5,551,328 | 221,709 | 198,686 | 93,066 |

| | | | | | | | |
|---|---|---|---|---|---|---|---|
| ERR048259 | *S. flexneri* | Connor2015 | 6,321,411 | 6,009,671 | 205,480 | 61,695 | 44,565 |
| ERR048261 | *S. flexneri* | Connor2015 | 6,144,544 | 5,794,930 | 231,009 | 73,345 | 45,260 |
| ERR048265 | *S. flexneri* | Connor2015 | 4,896,731 | 4,642,466 | 168,772 | 47,880 | 37,613 |
| ERR048285 | *S. flexneri* | Connor2015 | 5,599,862 | 5,283,998 | 194,590 | 66,862 | 54,412 |
| ERR048286 | *S. flexneri* | Connor2015 | 4,846,679 | 4,591,186 | 154,334 | 57,332 | 43,827 |
| ERR048287 | *S. flexneri* | Connor2015 | 5,293,614 | 5,007,899 | 172,322 | 65,663 | 47,730 |
| ERR048288 | *S. flexneri* | Connor2015 | 4,711,512 | 4,457,944 | 154,935 | 56,142 | 42,491 |
| ERR048290 | *S. flexneri* | Connor2015 | 6,504,135 | 6,137,470 | 220,429 | 87,677 | 58,559 |
| ERR048295 | *S. flexneri* | Connor2015 | 5,498,327 | 5,210,665 | 176,816 | 61,575 | 49,271 |
| ERR048296 | *S. flexneri* | Connor2015 | 6,079,064 | 5,822,210 | 159,229 | 57,843 | 39,782 |
| ERR048300 | *S. flexneri* | Connor2015 | 5,632,013 | 5,393,155 | 153,272 | 47,957 | 37,629 |
| ERR048302 | *S. flexneri* | Connor2015 | 2,014,889 | 1,931,091 | 52,916 | 17,636 | 13,246 |
| ERR048304 | *S. flexneri* | Connor2015 | 5,222,850 | 4,984,477 | 146,366 | 54,519 | 37,488 |
| ERR048306 | *S. flexneri* | Connor2015 | 5,181,072 | 4,957,469 | 141,322 | 46,906 | 35,375 |
| ERR048311 | *S. flexneri* | Connor2015 | 5,723,095 | 5,477,800 | 155,262 | 50,982 | 39,051 |
| ERR048312 | *S. flexneri* | Connor2015 | 5,608,447 | 5,375,858 | 145,921 | 49,807 | 36,861 |
| ERR048313 | *S. flexneri* | Connor2015 | 5,382,635 | 5,138,568 | 159,608 | 45,891 | 38,568 |
| ERR048315 | *S. flexneri* | Connor2015 | 3,541,498 | 3,376,219 | 104,181 | 37,383 | 23,715 |
| ERR048316 | *S. flexneri* | Connor2015 | 5,789,756 | 5,515,205 | 171,415 | 62,113 | 41,023 |
| ERR048317 | *S. flexneri* | Connor2015 | 3,683,651 | 3,520,490 | 103,923 | 33,097 | 26,141 |
| ERR048319 | *S. flexneri* | Connor2015 | 4,483,061 | 4,257,908 | 142,667 | 46,901 | 35,585 |
| ERR048320 | *S. flexneri* | Connor2015 | 6,026,596 | 5,728,588 | 190,876 | 59,595 | 47,537 |
| ERR048322 | *S. flexneri* | Connor2015 | 5,956,431 | 5,636,770 | 207,718 | 62,734 | 49,209 |
| ERR048329 | *S. flexneri* | Connor2015 | 4,387,202 | 4,161,734 | 144,653 | 44,109 | 36,706 |
| ERR048331 | *S. flexneri* | Connor2015 | 4,460,857 | 4,246,142 | 136,656 | 43,991 | 34,068 |
| ERR048332 | *S. flexneri* | Connor2015 | 5,875,054 | 5,573,094 | 193,625 | 60,455 | 47,880 |
| ERR048333 | *S. flexneri* | Connor2015 | 7,267,972 | 6,910,763 | 225,247 | 74,289 | 57,673 |
| ERR048334 | *S. flexneri* | Connor2015 | 6,118,520 | 5,828,481 | 183,661 | 61,300 | 45,078 |
| ERR048339 | *S. flexneri* | Connor2015 | 7,034,272 | 6,683,900 | 227,303 | 66,545 | 56,524 |
| ERR049152 | *S. flexneri* | Connor2015 | 15,872,102 | 14,110,217 | 730,728 | 672,984 | 358,173 |
| ERR126958 | *S. flexneri* | Connor2015 | 2,580,305 | 2,435,948 | 102,675 | 26,144 | 15,538 |
| ERR127015 | *S. flexneri* | Connor2015 | 3,268,542 | 3,093,570 | 112,448 | 42,793 | 19,731 |
| ERR127017 | *S. flexneri* | Connor2015 | 2,746,340 | 2,593,674 | 99,240 | 35,798 | 17,628 |
| ERR127019 | *S. flexneri* | Connor2015 | 2,857,739 | 2,707,838 | 95,749 | 36,887 | 17,265 |
| ERR127032 | *S. flexneri* | Connor2015 | 2,960,191 | 2,807,994 | 95,248 | 40,208 | 16,741 |
| ERR127034 | *S. flexneri* | Connor2015 | 3,043,011 | 2,877,331 | 107,764 | 40,586 | 17,330 |
| ERR127035 | *S. flexneri* | Connor2015 | 2,834,988 | 2,683,968 | 96,927 | 37,190 | 16,903 |
| ERR127036 | *S. flexneri* | Connor2015 | 2,640,189 | 2,500,543 | 89,253 | 35,264 | 15,129 |
| ERR127037 | *S. flexneri* | Connor2015 | 2,592,103 | 2,454,671 | 89,274 | 32,956 | 15,202 |
| ERR127038 | *S. flexneri* | Connor2015 | 2,734,181 | 2,591,832 | 90,599 | 36,296 | 15,454 |
| ERR127039 | *S. flexneri* | Connor2015 | 3,190,505 | 3,022,448 | 106,525 | 42,894 | 18,638 |
| ERR127040 | *S. flexneri* | Connor2015 | 3,120,671 | 2,956,878 | 103,278 | 43,022 | 17,493 |
| ERR127041 | *S. flexneri* | Connor2015 | 2,764,632 | 2,616,278 | 94,572 | 37,238 | 16,544 |
| ERR127042 | *S. flexneri* | Connor2015 | 2,856,174 | 2,707,681 | 93,962 | 38,415 | 16,116 |
| ERR127043 | *S. flexneri* | Connor2015 | 2,866,238 | 2,713,540 | 97,727 | 38,451 | 16,520 |
| ERR127044 | *S. flexneri* | Connor2015 | 2,762,846 | 2,624,589 | 87,451 | 35,956 | 14,850 |
| ERR127046 | *S. flexneri* | Connor2015 | 8,633,405 | 8,169,771 | 319,007 | 90,916 | 53,711 |
| ERR127047 | *S. flexneri* | Connor2015 | 9,254,269 | 8,753,246 | 345,648 | 95,593 | 59,782 |
| ERR127048 | *S. flexneri* | Connor2015 | 11,985,789 | 11,312,073 | 467,437 | 125,948 | 80,331 |
| ERR200344 | *S. flexneri* | Connor2015 | 1,678,942 | 1,618,223 | 49,301 | 6,212 | 5,206 |

| | | | | | | | |
|---|---|---|---|---|---|---|---|
| ERR200360 | *S. flexneri* | Connor2015 | 2,091,430 | 2,006,692 | 66,828 | 10,440 | 7,470 |
| ERR200365 | *S. flexneri* | Connor2015 | 2,106,014 | 2,022,048 | 65,580 | 10,775 | 7,611 |
| ERR200370 | *S. flexneri* | Connor2015 | 2,102,644 | 2,017,796 | 66,715 | 10,901 | 7,232 |
| ERR200378 | *S. flexneri* | Connor2015 | 2,120,614 | 2,042,583 | 60,212 | 10,709 | 7,110 |
| ERR200379 | *S. flexneri* | Connor2015 | 1,249,225 | 1,202,392 | 36,361 | 6,611 | 3,861 |
| ERR200390 | *S. flexneri* | Connor2015 | 1,905,186 | 1,821,835 | 66,263 | 9,442 | 7,646 |
| ERR200392 | *S. flexneri* | Connor2015 | 2,384,791 | 2,290,939 | 74,039 | 11,540 | 8,273 |
| ERR200393 | *S. flexneri* | Connor2015 | 1,274,546 | 1,227,462 | 36,011 | 6,830 | 4,243 |
| ERR200402 | *S. flexneri* | Connor2015 | 1,730,147 | 1,657,788 | 57,348 | 8,484 | 6,527 |
| ERR200403 | *S. flexneri* | Connor2015 | 1,307,013 | 1,252,753 | 42,769 | 6,623 | 4,868 |
| ERR200405 | *S. flexneri* | Connor2015 | 1,503,673 | 1,441,446 | 49,077 | 7,781 | 5,369 |
| ERR200413 | *S. flexneri* | Connor2015 | 1,578,641 | 1,517,360 | 47,435 | 8,312 | 5,534 |
| ERR200414 | *S. flexneri* | Connor2015 | 1,343,923 | 1,290,688 | 41,449 | 6,900 | 4,886 |
| ERR200415 | *S. flexneri* | Connor2015 | 2,794,820 | 2,679,747 | 90,645 | 14,260 | 10,168 |
| ERR217012 | *S. flexneri* | Connor2015 | 1,629,933 | 1,572,027 | 42,332 | 10,318 | 5,256 |
| ERR217013 | *S. flexneri* | Connor2015 | 1,273,537 | 1,240,575 | 20,030 | 9,727 | 3,205 |
| ERR217015 | *S. flexneri* | Connor2015 | 1,961,426 | 1,892,615 | 49,925 | 12,675 | 6,211 |
| ERR217016 | *S. flexneri* | Connor2015 | 2,115,892 | 2,042,422 | 53,036 | 13,821 | 6,613 |
| ERR217022 | *S. flexneri* | Connor2015 | 1,470,096 | 1,410,945 | 44,848 | 9,186 | 5,117 |
| ERR217023 | *S. flexneri* | Connor2015 | 1,603,306 | 1,533,546 | 53,715 | 10,366 | 5,679 |
| ERR217024 | *S. flexneri* | Connor2015 | 1,292,730 | 1,242,091 | 37,894 | 8,197 | 4,548 |
| ERR217026 | *S. flexneri* | Connor2015 | 1,450,001 | 1,394,461 | 41,264 | 9,248 | 5,028 |
| ERR217028 | *S. flexneri* | Connor2015 | 1,230,718 | 1,187,523 | 30,764 | 8,363 | 4,068 |
| ERR217030 | *S. flexneri* | Connor2015 | 1,154,036 | 1,106,095 | 36,248 | 7,370 | 4,323 |
| ERR217031 | *S. flexneri* | Connor2015 | 1,609,073 | 1,547,507 | 45,647 | 10,334 | 5,585 |
| ERR217032 | *S. flexneri* | Connor2015 | 1,015,786 | 966,428 | 38,908 | 6,209 | 4,241 |
| ERR217033 | *S. flexneri* | Connor2015 | 1,988,914 | 1,912,379 | 56,416 | 13,236 | 6,883 |
| ERR217047 | *S. flexneri* | Connor2015 | 1,163,347 | 1,108,730 | 42,355 | 7,713 | 4,549 |
| ERR217080 | *S. flexneri* | Connor2015 | 4,265,982 | 4,115,807 | 108,306 | 28,323 | 13,546 |
| ERR217081 | *S. flexneri* | Connor2015 | 1,545,688 | 1,489,886 | 40,647 | 10,249 | 4,906 |
| ERR217084 | *S. flexneri* | Connor2015 | 1,695,512 | 1,638,331 | 40,742 | 11,322 | 5,117 |
| ERR559526 | *S. flexneri* | Connor2015 | 1,154,823 | 1,069,423 | 67,365 | 7,468 | 10,567 |
| ERR832453 | *S. flexneri* | Connor2015 | 2,215,582 | 2,122,274 | 68,486 | 15,570 | 9,252 |
| ERR832456 | *S. flexneri* | Connor2015 | 2,387,788 | 2,285,655 | 75,071 | 16,694 | 10,368 |
| ERR832457 | *S. flexneri* | Connor2015 | 2,749,942 | 2,631,643 | 86,480 | 19,542 | 12,277 |
| ERR832459 | *S. flexneri* | Connor2015 | 2,679,853 | 2,564,216 | 85,492 | 18,403 | 11,742 |
| ERR832460 | *S. flexneri* | Connor2015 | 2,665,298 | 2,552,882 | 82,670 | 18,488 | 11,258 |
| ERR832461 | *S. flexneri* | Connor2015 | 2,509,462 | 2,397,424 | 82,690 | 17,794 | 11,554 |
| ERR832462 | *S. flexneri* | Connor2015 | 2,656,735 | 2,542,736 | 83,469 | 18,669 | 11,861 |
| ERR832464 | *S. flexneri* | Connor2015 | 2,154,943 | 2,027,716 | 96,687 | 16,166 | 14,374 |
| ERR832465 | *S. flexneri* | Connor2015 | 2,547,321 | 2,433,186 | 85,141 | 17,491 | 11,503 |
| ERR832467 | *S. flexneri* | Connor2015 | 2,463,007 | 2,356,015 | 78,354 | 18,369 | 10,269 |
| ERR832468 | *S. flexneri* | Connor2015 | 2,867,001 | 2,750,131 | 85,105 | 20,068 | 11,697 |
| ERR832470 | *S. flexneri* | Connor2015 | 2,830,562 | 2,710,804 | 88,669 | 19,820 | 11,269 |
| ERR832471 | *S. flexneri* | Connor2015 | 2,640,106 | 2,525,927 | 84,382 | 18,381 | 11,416 |
| ERR832473 | *S. flexneri* | Connor2015 | 2,543,396 | 2,440,013 | 75,076 | 18,148 | 10,159 |
| ERR832474 | *S. flexneri* | Connor2015 | 3,028,429 | 2,908,585 | 87,721 | 20,466 | 11,657 |
| ERR832477 | *S. flexneri* | Connor2015 | 2,629,715 | 2,529,967 | 72,110 | 18,030 | 9,608 |
| ERR832480 | *S. flexneri* | Connor2015 | 2,330,007 | 2,235,733 | 68,982 | 15,964 | 9,328 |
| ERR832481 | *S. flexneri* | Connor2015 | 2,986,181 | 2,874,015 | 80,818 | 20,402 | 10,946 |

| | | | | | | | |
|---|---|---|---|---|---|---|---|
| ERR832483 | *S. flexneri* | Connor2015 | 2,914,696 | 2,803,823 | 81,048 | 19,206 | 10,619 |
| ERR832485 | *S. flexneri* | Connor2015 | 2,704,496 | 2,600,641 | 74,450 | 18,982 | 10,423 |
| ERR832486 | *S. flexneri* | Connor2015 | 2,463,206 | 2,369,812 | 68,322 | 16,371 | 8,701 |
| ERR832487 | *S. flexneri* | Connor2015 | 2,599,866 | 2,494,836 | 77,271 | 17,958 | 9,801 |
| ERR832489 | *S. flexneri* | Connor2015 | 2,436,299 | 2,335,429 | 74,431 | 16,561 | 9,878 |
| ERR832490 | *S. flexneri* | Connor2015 | 2,375,466 | 2,276,298 | 74,312 | 15,209 | 9,647 |
| ERR832491 | *S. flexneri* | Connor2015 | 2,249,088 | 2,156,686 | 67,365 | 15,704 | 9,333 |
| ERR832492 | *S. flexneri* | Connor2015 | 2,612,677 | 2,503,353 | 79,307 | 19,064 | 10,953 |
| ERR832494 | *S. flexneri* | Connor2015 | 2,221,230 | 2,135,369 | 61,592 | 15,713 | 8,556 |
| S13BD00302 | *S. flexneri* | NRC | 511,893 | 446,439 | 57,019 | 2,525 | 5,910 |
| S13BD00535 | *S. flexneri* | NRC | 275,687 | 222,976 | 46,414 | 1,359 | 4,938 |
| S13BD00702 | *S. flexneri* | NRC | 736,715 | 642,687 | 82,298 | 3,199 | 8,531 |
| S13BD00854 | *S. flexneri* | NRC | 720,185 | 654,716 | 55,722 | 4,195 | 5,552 |
| S13BD01310 | *S. flexneri* | NRC | 697,952 | 630,948 | 58,038 | 3,430 | 5,536 |
| S13BD01692 | *S. flexneri* | NRC | 496,294 | 449,012 | 39,157 | 3,742 | 4,383 |
| S13BD02195 | *S. flexneri* | NRC | 454,726 | 407,518 | 39,840 | 3,178 | 4,190 |
| S13BD02470 | *S. flexneri* | NRC | 527,175 | 470,382 | 50,118 | 2,227 | 4,448 |
| S13BD03084 | *S. flexneri* | NRC | 841,760 | 752,945 | 80,870 | 2,170 | 5,775 |
| S13BD04017 | *S. flexneri* | NRC | 677,168 | 617,333 | 53,620 | 2,062 | 4,153 |
| S13BD04231 | *S. flexneri* | NRC | 336,081 | 290,618 | 39,447 | 1,619 | 4,397 |
| S14BD00799 | *S. flexneri* | NRC | 862,376 | 774,006 | 77,795 | 3,552 | 7,023 |
| S14BD01131 | *S. flexneri* | NRC | 691,381 | 613,574 | 71,466 | 1,599 | 4,742 |
| S14BD01142 | *S. flexneri* | NRC | 1,166,163 | 1,087,372 | 70,074 | 3,378 | 5,339 |
| S14BD01714 | *S. flexneri* | NRC | 589,662 | 529,394 | 54,493 | 1,584 | 4,191 |
| S14BD02502 | *S. flexneri* | NRC | 685,542 | 640,393 | 38,569 | 2,194 | 4,386 |
| S14BD02795 | *S. flexneri* | NRC | 1,056,566 | 940,550 | 103,052 | 4,956 | 8,008 |
| S14BD05234 | *S. flexneri* | NRC | 340,055 | 215,051 | 99,309 | 8,881 | 16,814 |
| S14BD05285 | *S. flexneri* | NRC | 427,038 | 322,602 | 86,095 | 6,672 | 11,669 |
| S15BD00055 | *S. flexneri* | NRC | 349,435 | 279,708 | 51,089 | 9,116 | 9,522 |
| S15BD00457 | *S. flexneri* | NRC | 632,861 | 456,899 | 149,280 | 6,677 | 20,005 |
| S15BD00494 | *S. flexneri* | NRC | 626,492 | 478,017 | 115,101 | 12,978 | 20,396 |
| S15BD02368 | *S. flexneri* | NRC | 696,036 | 510,254 | 156,511 | 9,147 | 20,124 |
| S15BD02726 | *S. flexneri* | NRC | 615,604 | 575,082 | 34,630 | 2,129 | 3,763 |
| S15BD02867 | *S. flexneri* | NRC | 762,654 | 684,255 | 65,067 | 6,063 | 7,269 |
| S15BD04659 | *S. flexneri* | NRC | 697,200 | 483,034 | 183,882 | 7,545 | 22,739 |
| S15BD05228 | *S. flexneri* | NRC | 587,731 | 511,231 | 66,679 | 2,651 | 7,170 |
| S15BD06353 | *S. flexneri* | NRC | 501,544 | 447,520 | 48,819 | 1,447 | 3,758 |
| S15BD06515 | *S. flexneri* | NRC | 211,394 | 141,990 | 62,106 | 1,602 | 5,696 |
| S15BD08204 | *S. flexneri* | NRC | 629,861 | 581,533 | 42,082 | 2,003 | 4,243 |
| S15BD08762 | *S. flexneri* | NRC | 768,420 | 636,161 | 115,686 | 3,707 | 12,866 |
| S15BD09335 | *S. flexneri* | NRC | 776,411 | 730,114 | 39,678 | 2,525 | 4,094 |
| S15BD09453 | *S. flexneri* | NRC | 636,726 | 584,470 | 46,619 | 1,940 | 3,697 |
| S15BD09529 | *S. flexneri* | NRC | 144,496 | 113,325 | 26,704 | 1,030 | 3,437 |
| S16BD00148 | *S. flexneri* | NRC | 348,079 | 287,754 | 51,848 | 2,419 | 6,058 |
| S16BD00463 | *S. flexneri* | NRC | 334,163 | 230,988 | 92,988 | 1,840 | 8,347 |
| S16BD01789 | *S. flexneri* | NRC | 471,997 | 433,576 | 33,335 | 1,727 | 3,359 |
| S16BD01991 | *S. flexneri* | NRC | 888,669 | 795,499 | 83,629 | 2,207 | 7,334 |
| S16BD02637 | *S. flexneri* | NRC | 540,538 | 479,765 | 54,840 | 1,345 | 4,588 |
| S16BD02856 | *S. flexneri* | NRC | 521,870 | 472,269 | 43,920 | 1,559 | 4,122 |
| S16BD03590 | *S. flexneri* | NRC | 404,822 | 278,973 | 113,163 | 2,547 | 10,139 |

| | | | | | | | |
|---|---|---|---|---|---|---|---|
| S16BD03881 | *S. flexneri* | NRC | 365,627 | 257,943 | 96,036 | 2,580 | 9,068 |
| S16BD04652 | *S. flexneri* | NRC | 599,841 | 555,121 | 38,660 | 2,168 | 3,892 |
| S16BD07706 | *S. flexneri* | NRC | 454,895 | 401,685 | 47,824 | 1,123 | 4,263 |
| S17BD00498 | *S. flexneri* | NRC | 548,579 | 521,681 | 22,334 | 2,133 | 2,431 |
| S17BD00886 | *S. flexneri* | NRC | 459,664 | 431,997 | 23,242 | 1,975 | 2,450 |
| S17BD02190 | *S. flexneri* | NRC | 560,479 | 535,068 | 20,389 | 2,498 | 2,524 |
| S17BD02300 | *S. flexneri* | NRC | 493,413 | 471,759 | 17,195 | 2,503 | 1,956 |
| S17BD03514 | *S. flexneri* | NRC | 375,321 | 351,014 | 21,468 | 1,058 | 1,781 |
| S17BD04033 | *S. flexneri* | NRC | 602,611 | 547,644 | 48,628 | 1,669 | 4,670 |
| S17BD04223 | *S. flexneri* | NRC | 470,188 | 439,110 | 27,206 | 1,329 | 2,543 |
| S17BD04752 | *S. flexneri* | NRC | 454,909 | 429,134 | 22,872 | 996 | 1,907 |
| S17BD06364 | *S. flexneri* | NRC | 674,116 | 616,280 | 50,879 | 2,152 | 4,805 |
| S17BD07654 | *S. flexneri* | NRC | 651,375 | 598,312 | 46,857 | 1,898 | 4,308 |
| S17BD08054 | *S. flexneri* | NRC | 542,382 | 514,565 | 23,066 | 2,166 | 2,585 |
| S17BD08194 | *S. flexneri* | NRC | 434,942 | 399,373 | 31,336 | 1,387 | 2,846 |
| S17BD08237 | *S. flexneri* | NRC | 562,049 | 534,550 | 22,510 | 2,407 | 2,582 |
| S18BD00005 | *S. flexneri* | NRC | 524,166 | 492,256 | 27,633 | 1,674 | 2,603 |
| S18BD00006 | *S. flexneri* | NRC | 497,809 | 468,054 | 25,506 | 1,724 | 2,525 |
| S18BD01109 | *S. flexneri* | NRC | 383,148 | 347,039 | 30,925 | 2,041 | 3,143 |
| S18BD01126 | *S. flexneri* | NRC | 493,475 | 467,614 | 21,342 | 2,194 | 2,325 |
| S18BD01471 | *S. flexneri* | NRC | 463,957 | 439,038 | 20,954 | 1,787 | 2,178 |
| ERR316291 | *S. sonnei* | Baker2017 | 1,699,564 | 1,456,967 | 213,073 | 11,774 | 17,750 |
| ERR316299 | *S. sonnei* | Baker2017 | 2,409,018 | 2,154,994 | 217,759 | 15,731 | 20,534 |
| ERR316306 | *S. sonnei* | Baker2017 | 2,299,102 | 2,159,975 | 107,981 | 16,075 | 15,071 |
| ERR024604 | *S. sonnei* | Holt2012 | 1,175,438 | 1,010,377 | 106,822 | 28,577 | 29,662 |
| ERR024605 | *S. sonnei* | Holt2012 | 3,337,272 | 2,950,114 | 223,803 | 84,968 | 78,387 |
| ERR024606 | *S. sonnei* | Holt2012 | 3,590,909 | 3,195,400 | 216,482 | 91,240 | 87,787 |
| ERR024607 | *S. sonnei* | Holt2012 | 2,668,107 | 2,343,460 | 190,392 | 68,548 | 65,707 |
| ERR024608 | *S. sonnei* | Holt2012 | 3,988,300 | 3,474,731 | 315,443 | 98,528 | 99,598 |
| ERR024609 | *S. sonnei* | Holt2012 | 3,609,528 | 3,182,800 | 250,336 | 88,769 | 87,623 |
| ERR024610 | *S. sonnei* | Holt2012 | 2,406,375 | 2,126,284 | 165,835 | 58,831 | 55,425 |
| ERR024611 | *S. sonnei* | Holt2012 | 2,014,908 | 1,798,189 | 115,215 | 54,908 | 46,596 |
| ERR024612 | *S. sonnei* | Holt2012 | 2,160,326 | 1,917,619 | 133,384 | 58,011 | 51,312 |
| ERR024616 | *S. sonnei* | Holt2012 | 2,676,024 | 2,350,715 | 188,905 | 74,648 | 61,756 |
| ERR024617 | *S. sonnei* | Holt2012 | 2,587,188 | 2,297,120 | 161,184 | 69,510 | 59,374 |
| ERR024618 | *S. sonnei* | Holt2012 | 2,256,242 | 1,980,405 | 159,321 | 61,395 | 55,121 |
| ERR024619 | *S. sonnei* | Holt2012 | 1,672,595 | 1,484,136 | 103,125 | 46,745 | 38,589 |
| ERR024620 | *S. sonnei* | Holt2012 | 2,318,878 | 1,992,183 | 203,526 | 63,405 | 59,764 |
| ERR024621 | *S. sonnei* | Holt2012 | 2,487,954 | 2,196,270 | 166,458 | 66,308 | 58,918 |
| ERR024622 | *S. sonnei* | Holt2012 | 2,749,119 | 2,415,379 | 195,075 | 72,680 | 65,985 |
| ERR024625 | *S. sonnei* | Holt2012 | 1,830,508 | 1,593,838 | 141,176 | 50,348 | 45,146 |
| ERR024626 | *S. sonnei* | Holt2012 | 2,216,786 | 1,957,664 | 146,294 | 60,884 | 51,944 |
| ERR024627 | *S. sonnei* | Holt2012 | 2,176,527 | 1,928,782 | 136,061 | 61,776 | 49,908 |
| ERR025682 | *S. sonnei* | Holt2012 | 2,392,044 | 1,849,852 | 455,558 | 36,046 | 50,588 |
| ERR025683 | *S. sonnei* | Holt2012 | 2,075,960 | 1,518,621 | 474,736 | 31,136 | 51,467 |
| ERR025685 | *S. sonnei* | Holt2012 | 2,725,289 | 2,377,260 | 265,742 | 45,426 | 36,861 |
| ERR025686 | *S. sonnei* | Holt2012 | 2,778,905 | 2,232,002 | 444,944 | 44,366 | 57,593 |
| ERR025687 | *S. sonnei* | Holt2012 | 2,106,468 | 1,623,179 | 407,302 | 31,524 | 44,463 |
| ERR025689 | *S. sonnei* | Holt2012 | 2,172,655 | 1,523,985 | 558,648 | 33,569 | 56,453 |
| ERR025690 | *S. sonnei* | Holt2012 | 1,708,772 | 1,396,055 | 255,817 | 26,342 | 30,558 |

| | | | | | | | |
|---|---|---|---|---|---|---|---|
| ERR025691 | *S. sonnei* | Holt2012 | 2,417,644 | 1,957,762 | 363,961 | 49,543 | 46,378 |
| ERR025692 | *S. sonnei* | Holt2012 | 2,640,217 | 2,010,888 | 528,468 | 39,884 | 60,977 |
| ERR025693 | *S. sonnei* | Holt2012 | 2,527,577 | 2,053,776 | 388,002 | 39,337 | 46,462 |
| ERR025695 | *S. sonnei* | Holt2012 | 1,489,327 | 1,219,011 | 228,687 | 18,208 | 23,421 |
| ERR025697 | *S. sonnei* | Holt2012 | 1,610,234 | 1,323,564 | 238,369 | 21,262 | 27,039 |
| ERR025698 | *S. sonnei* | Holt2012 | 2,933,409 | 2,629,564 | 234,799 | 37,834 | 31,212 |
| ERR025699 | *S. sonnei* | Holt2012 | 2,841,022 | 2,373,235 | 385,468 | 36,889 | 45,430 |
| ERR025700 | *S. sonnei* | Holt2012 | 1,633,451 | 1,262,391 | 320,569 | 19,583 | 30,908 |
| ERR025701 | *S. sonnei* | Holt2012 | 2,517,832 | 2,066,296 | 379,842 | 31,946 | 39,748 |
| ERR025702 | *S. sonnei* | Holt2012 | 1,578,030 | 1,240,602 | 288,745 | 20,110 | 28,573 |
| ERR025703 | *S. sonnei* | Holt2012 | 2,077,567 | 1,833,391 | 192,141 | 26,856 | 25,179 |
| ERR025704 | *S. sonnei* | Holt2012 | 1,905,660 | 1,615,727 | 230,492 | 32,313 | 27,128 |
| ERR025705 | *S. sonnei* | Holt2012 | 2,290,854 | 1,906,358 | 320,703 | 28,715 | 35,078 |
| ERR025706 | *S. sonnei* | Holt2012 | 2,012,650 | 1,652,702 | 302,694 | 24,606 | 32,648 |
| ERR025708 | *S. sonnei* | Holt2012 | 1,221,173 | 830,621 | 336,856 | 19,399 | 34,297 |
| ERR025709 | *S. sonnei* | Holt2012 | 2,135,245 | 1,278,673 | 754,688 | 29,654 | 72,230 |
| ERR025710 | *S. sonnei* | Holt2012 | 2,464,277 | 1,639,444 | 711,346 | 37,872 | 75,615 |
| ERR025711 | *S. sonnei* | Holt2012 | 3,714,081 | 3,028,537 | 544,597 | 69,475 | 71,472 |
| ERR025712 | *S. sonnei* | Holt2012 | 3,610,064 | 2,642,841 | 807,325 | 61,788 | 98,110 |
| ERR025713 | *S. sonnei* | Holt2012 | 3,331,168 | 2,405,865 | 784,717 | 55,320 | 85,266 |
| ERR025714 | *S. sonnei* | Holt2012 | 2,448,287 | 1,532,296 | 802,118 | 35,880 | 77,993 |
| ERR025715 | *S. sonnei* | Holt2012 | 2,248,626 | 1,274,884 | 867,574 | 29,452 | 76,716 |
| ERR025716 | *S. sonnei* | Holt2012 | 3,174,339 | 2,571,142 | 480,873 | 59,257 | 63,067 |
| ERR025717 | *S. sonnei* | Holt2012 | 2,886,627 | 2,104,183 | 650,304 | 53,099 | 79,041 |
| ERR025718 | *S. sonnei* | Holt2012 | 3,188,177 | 2,365,980 | 688,725 | 54,456 | 79,016 |
| ERR025719 | *S. sonnei* | Holt2012 | 3,166,379 | 2,173,895 | 852,744 | 50,167 | 89,573 |
| ERR025721 | *S. sonnei* | Holt2012 | 2,159,627 | 1,623,448 | 448,775 | 33,005 | 54,399 |
| ERR025724 | *S. sonnei* | Holt2012 | 2,763,615 | 2,388,766 | 288,728 | 42,999 | 43,122 |
| ERR025725 | *S. sonnei* | Holt2012 | 2,519,956 | 1,976,885 | 439,799 | 39,707 | 63,565 |
| ERR025726 | *S. sonnei* | Holt2012 | 2,197,147 | 1,729,607 | 385,135 | 34,158 | 48,247 |
| ERR025727 | *S. sonnei* | Holt2012 | 2,460,594 | 1,846,233 | 509,037 | 41,139 | 64,185 |
| ERR025729 | *S. sonnei* | Holt2012 | 1,956,135 | 1,615,831 | 273,480 | 30,386 | 36,438 |
| ERR025730 | *S. sonnei* | Holt2012 | 1,793,362 | 1,334,482 | 363,833 | 44,845 | 50,202 |
| ERR025731 | *S. sonnei* | Holt2012 | 2,114,883 | 1,639,791 | 388,738 | 33,868 | 52,486 |
| ERR025732 | *S. sonnei* | Holt2012 | 9,183,277 | 7,053,222 | 1,741,655 | 148,392 | 240,008 |
| ERR025734 | *S. sonnei* | Holt2012 | 3,557,919 | 2,857,184 | 534,642 | 72,869 | 93,224 |
| ERR025735 | *S. sonnei* | Holt2012 | 2,214,773 | 1,732,880 | 366,371 | 50,987 | 64,535 |
| ERR025737 | *S. sonnei* | Holt2012 | 2,931,394 | 2,316,042 | 464,403 | 62,199 | 88,750 |
| ERR025738 | *S. sonnei* | Holt2012 | 3,165,581 | 2,435,109 | 565,724 | 69,715 | 95,033 |
| ERR025741 | *S. sonnei* | Holt2012 | 2,655,178 | 2,215,611 | 323,380 | 56,188 | 59,999 |
| ERR025742 | *S. sonnei* | Holt2012 | 2,740,406 | 2,202,285 | 379,365 | 87,274 | 71,482 |
| ERR025743 | *S. sonnei* | Holt2012 | 3,080,426 | 2,466,474 | 462,173 | 66,187 | 85,592 |
| ERR025744 | *S. sonnei* | Holt2012 | 2,795,511 | 2,171,408 | 473,140 | 64,527 | 86,436 |
| ERR025746 | *S. sonnei* | Holt2012 | 4,101,207 | 3,069,751 | 873,338 | 59,419 | 98,699 |
| ERR025747 | *S. sonnei* | Holt2012 | 2,415,202 | 1,660,717 | 654,316 | 32,791 | 67,378 |
| ERR025749 | *S. sonnei* | Holt2012 | 4,060,301 | 2,940,665 | 946,456 | 57,328 | 115,852 |
| ERR025750 | *S. sonnei* | Holt2012 | 3,021,931 | 2,245,326 | 657,841 | 43,636 | 75,128 |
| ERR025751 | *S. sonnei* | Holt2012 | 1,996,064 | 1,340,784 | 570,564 | 26,734 | 57,982 |
| ERR025752 | *S. sonnei* | Holt2012 | 2,448,503 | 1,331,774 | 1,002,931 | 27,860 | 85,938 |
| ERR025753 | *S. sonnei* | Holt2012 | 3,589,582 | 2,917,549 | 545,137 | 56,725 | 70,171 |

| | | | | | | | |
|---|---|---|---|---|---|---|---|
| ERR025754 | *S. sonnei* | Holt2012 | 3,204,173 | 2,249,802 | 801,594 | 55,637 | 97,140 |
| ERR025755 | *S. sonnei* | Holt2012 | 4,042,513 | 2,824,464 | 1,040,036 | 55,935 | 122,078 |
| ERR025756 | *S. sonnei* | Holt2012 | 3,203,923 | 2,173,145 | 891,265 | 42,560 | 96,953 |
| ERR025758 | *S. sonnei* | Holt2012 | 3,945,730 | 2,970,933 | 817,130 | 58,868 | 98,799 |
| ERR025759 | *S. sonnei* | Holt2012 | 3,399,624 | 2,179,700 | 1,054,020 | 48,774 | 117,130 |
| ERR025761 | *S. sonnei* | Holt2012 | 3,566,648 | 2,941,442 | 495,155 | 57,512 | 72,539 |
| ERR025762 | *S. sonnei* | Holt2012 | 2,981,763 | 2,347,604 | 521,564 | 46,423 | 66,172 |
| ERR025763 | *S. sonnei* | Holt2012 | 2,297,222 | 1,488,459 | 697,143 | 34,205 | 77,415 |
| ERR025765 | *S. sonnei* | Holt2012 | 2,777,114 | 2,252,740 | 427,438 | 43,737 | 53,199 |
| ERR025767 | *S. sonnei* | Holt2012 | 4,473,018 | 3,289,330 | 985,263 | 69,013 | 129,412 |
| ERR025768 | *S. sonnei* | Holt2012 | 2,506,199 | 1,819,895 | 576,681 | 37,602 | 72,021 |
| ERR028671 | *S. sonnei* | Holt2012 | 872,883 | 788,357 | 76,550 | 4,806 | 3,170 |
| ERR028672 | *S. sonnei* | Holt2012 | 1,169,414 | 1,047,039 | 111,445 | 6,464 | 4,466 |
| ERR028673 | *S. sonnei* | Holt2012 | 8,557,096 | 7,570,093 | 900,238 | 46,264 | 40,501 |
| ERR028674 | *S. sonnei* | Holt2012 | 2,717,175 | 2,414,003 | 277,183 | 15,043 | 10,946 |
| ERR028675 | *S. sonnei* | Holt2012 | 1,164,838 | 1,045,456 | 108,366 | 6,424 | 4,592 |
| ERR028676 | *S. sonnei* | Holt2012 | 1,857,716 | 1,686,610 | 153,910 | 10,189 | 7,007 |
| ERR028677 | *S. sonnei* | Holt2012 | 1,140,287 | 1,010,967 | 118,353 | 6,117 | 4,850 |
| ERR028678 | *S. sonnei* | Holt2012 | 870,782 | 782,641 | 80,148 | 4,813 | 3,180 |
| ERR028679 | *S. sonnei* | Holt2012 | 1,492,095 | 1,381,516 | 97,213 | 8,276 | 5,090 |
| ERR028680 | *S. sonnei* | Holt2012 | 1,062,538 | 929,075 | 123,086 | 5,570 | 4,807 |
| ERR028681 | *S. sonnei* | Holt2012 | 827,528 | 725,208 | 94,362 | 4,490 | 3,468 |
| ERR028684 | *S. sonnei* | Holt2012 | 2,239,763 | 2,092,860 | 126,121 | 12,712 | 8,070 |
| ERR028685 | *S. sonnei* | Holt2012 | 3,417,308 | 3,193,232 | 191,614 | 19,791 | 12,671 |
| ERR028686 | *S. sonnei* | Holt2012 | 1,402,588 | 1,302,795 | 85,081 | 8,860 | 5,852 |
| ERR028687 | *S. sonnei* | Holt2012 | 2,303,980 | 2,157,377 | 124,885 | 13,575 | 8,143 |
| ERR028688 | *S. sonnei* | Holt2012 | 1,761,693 | 1,614,635 | 128,746 | 10,756 | 7,556 |
| ERR028689 | *S. sonnei* | Holt2012 | 3,104,904 | 2,913,529 | 162,452 | 17,613 | 11,310 |
| ERR028690 | *S. sonnei* | Holt2012 | 1,497,909 | 1,393,171 | 90,146 | 8,767 | 5,825 |
| ERR028691 | *S. sonnei* | Holt2012 | 1,048,827 | 973,610 | 65,233 | 6,082 | 3,902 |
| ERR028692 | *S. sonnei* | Holt2012 | 2,461,815 | 2,296,810 | 141,047 | 14,418 | 9,540 |
| ERR028693 | *S. sonnei* | Holt2012 | 1,818,972 | 1,690,229 | 111,133 | 10,428 | 7,182 |
| ERR028694 | *S. sonnei* | Holt2012 | 1,972,334 | 1,816,571 | 135,724 | 11,980 | 8,059 |
| ERR028695 | *S. sonnei* | Holt2012 | 2,510,481 | 2,329,108 | 156,845 | 14,681 | 9,847 |
| ERR028697 | *S. sonnei* | Holt2012 | 4,648,002 | 4,178,061 | 370,490 | 52,529 | 46,922 |
| ERR028699 | *S. sonnei* | Holt2012 | 6,048,951 | 5,443,502 | 474,147 | 69,690 | 61,612 |
| ERR028700 | *S. sonnei* | Holt2012 | 3,143,513 | 2,824,565 | 250,354 | 36,142 | 32,452 |
| ERR028702 | *S. sonnei* | Holt2012 | 6,657,688 | 5,937,652 | 569,856 | 78,003 | 72,177 |
| ERR028703 | *S. sonnei* | Holt2012 | 7,243,136 | 6,547,669 | 543,327 | 78,276 | 73,864 |
| ERR028704 | *S. sonnei* | Holt2012 | 4,199,270 | 3,770,364 | 333,257 | 50,891 | 44,758 |
| ERR028705 | *S. sonnei* | Holt2012 | 4,219,763 | 3,739,210 | 387,058 | 48,063 | 45,432 |
| ERR028706 | *S. sonnei* | Holt2012 | 2,410,007 | 2,163,591 | 194,401 | 26,558 | 25,457 |
| S13BD00834 | *S. sonnei* | NRC | 665,860 | 589,614 | 64,543 | 4,231 | 7,472 |
| S13BD00906 | *S. sonnei* | NRC | 1,225,150 | 1,093,374 | 118,057 | 5,367 | 8,352 |
| S13BD02106 | *S. sonnei* | NRC | 507,005 | 442,188 | 56,291 | 2,456 | 6,070 |
| S13BD02107 | *S. sonnei* | NRC | 489,395 | 430,560 | 51,848 | 1,885 | 5,102 |
| S13BD02670 | *S. sonnei* | NRC | 494,486 | 427,831 | 59,111 | 1,830 | 5,714 |
| S13BD02671 | *S. sonnei* | NRC | 694,810 | 600,609 | 83,764 | 2,382 | 8,055 |
| S13BD03196 | *S. sonnei* | NRC | 591,614 | 528,955 | 53,871 | 2,941 | 5,847 |
| S13BD03288 | *S. sonnei* | NRC | 695,339 | 626,053 | 58,504 | 4,172 | 6,610 |

| | | | | | | | |
|---|---|---|---|---|---|---|---|
| S13BD03539 | *S. sonnei* | NRC | 512,773 | 462,646 | 42,346 | 3,097 | 4,684 |
| S13BD04360 | *S. sonnei* | NRC | 708,996 | 644,209 | 55,275 | 3,523 | 5,989 |
| S14BD00913 | *S. sonnei* | NRC | 812,374 | 728,190 | 72,944 | 3,718 | 7,522 |
| S14BD03054 | *S. sonnei* | NRC | 607,166 | 548,271 | 49,960 | 3,677 | 5,258 |
| S14BD03397 | *S. sonnei* | NRC | 847,067 | 605,602 | 207,714 | 8,840 | 24,911 |
| S14BD03573 | *S. sonnei* | NRC | 567,051 | 411,200 | 132,078 | 6,582 | 17,191 |
| S14BD04882 | *S. sonnei* | NRC | 563,557 | 408,393 | 131,255 | 5,579 | 18,330 |
| S14BD04971 | *S. sonnei* | NRC | 459,247 | 404,286 | 47,027 | 2,616 | 5,318 |
| S14BD05043 | *S. sonnei* | NRC | 425,034 | 309,262 | 90,806 | 11,160 | 13,806 |
| S15BD00569 | *S. sonnei* | NRC | 560,278 | 390,719 | 145,210 | 5,371 | 18,978 |
| S15BD00797 | *S. sonnei* | NRC | 433,514 | 295,550 | 124,047 | 2,650 | 11,267 |
| S15BD00889 | *S. sonnei* | NRC | 610,293 | 467,493 | 109,892 | 15,631 | 17,277 |
| S15BD00932 | *S. sonnei* | NRC | 350,440 | 262,998 | 68,393 | 7,462 | 11,587 |
| S15BD01283 | *S. sonnei* | NRC | 933,186 | 679,610 | 209,246 | 14,972 | 29,358 |
| S15BD02324 | *S. sonnei* | NRC | 566,435 | 493,564 | 63,262 | 2,558 | 7,051 |
| S15BD02325 | *S. sonnei* | NRC | 669,687 | 524,975 | 112,692 | 14,308 | 17,712 |
| S15BD02326 | *S. sonnei* | NRC | 689,690 | 510,847 | 150,604 | 7,572 | 20,667 |
| S15BD02407 | *S. sonnei* | NRC | 1,025,313 | 908,212 | 106,144 | 3,809 | 7,148 |
| S15BD02515 | *S. sonnei* | NRC | 817,762 | 738,639 | 67,630 | 5,159 | 6,334 |
| S15BD02737 | *S. sonnei* | NRC | 538,256 | 395,606 | 115,901 | 7,158 | 19,591 |
| S15BD02979 | *S. sonnei* | NRC | 372,927 | 306,923 | 58,206 | 1,637 | 6,161 |
| S15BD02995 | *S. sonnei* | NRC | 1,044,555 | 792,596 | 206,503 | 17,747 | 27,709 |
| S15BD03192 | *S. sonnei* | NRC | 368,909 | 270,720 | 82,297 | 4,636 | 11,256 |
| S15BD03801 | *S. sonnei* | NRC | 370,218 | 278,619 | 72,911 | 6,325 | 12,363 |
| S15BD03806 | *S. sonnei* | NRC | 744,297 | 647,750 | 86,421 | 2,908 | 7,218 |
| S15BD03807 | *S. sonnei* | NRC | 584,805 | 448,873 | 103,677 | 15,739 | 16,516 |
| S15BD03808 | *S. sonnei* | NRC | 509,450 | 458,476 | 44,020 | 2,520 | 4,434 |
| S15BD03850 | *S. sonnei* | NRC | 483,470 | 421,779 | 52,852 | 2,630 | 6,209 |
| S15BD04030 | *S. sonnei* | NRC | 660,699 | 575,152 | 74,856 | 2,712 | 7,979 |
| S15BD04031 | *S. sonnei* | NRC | 579,817 | 434,286 | 119,833 | 8,677 | 17,021 |
| S15BD04165 | *S. sonnei* | NRC | 621,042 | 439,565 | 156,119 | 5,486 | 19,872 |
| S15BD04166 | *S. sonnei* | NRC | 381,103 | 319,494 | 54,742 | 1,532 | 5,335 |
| S15BD04402 | *S. sonnei* | NRC | 810,414 | 759,808 | 42,990 | 3,084 | 4,532 |
| S15BD04792 | *S. sonnei* | NRC | 298,606 | 228,008 | 55,101 | 5,974 | 9,523 |
| S15BD04848 | *S. sonnei* | NRC | 436,934 | 325,930 | 91,478 | 6,098 | 13,428 |
| S15BD05940 | *S. sonnei* | NRC | 420,762 | 326,184 | 77,293 | 6,560 | 10,725 |
| S15BD06471 | *S. sonnei* | NRC | 476,891 | 368,472 | 87,041 | 7,013 | 14,365 |
| S15BD07017 | *S. sonnei* | NRC | 624,738 | 538,367 | 76,099 | 2,508 | 7,764 |
| S15BD07161 | *S. sonnei* | NRC | 751,196 | 633,238 | 103,377 | 4,137 | 10,444 |
| S15BD07522 | *S. sonnei* | NRC | 777,583 | 653,327 | 109,117 | 4,247 | 10,892 |
| S15BD09124 | *S. sonnei* | NRC | 327,116 | 265,856 | 52,160 | 2,741 | 6,359 |
| S15BD09162 | *S. sonnei* | NRC | 771,279 | 649,507 | 105,469 | 5,323 | 10,980 |
| S15BD09356 | *S. sonnei* | NRC | 331,434 | 223,553 | 97,345 | 2,016 | 8,520 |
| S15BD09628 | *S. sonnei* | NRC | 692,935 | 586,919 | 93,320 | 4,388 | 8,308 |
| S15BD09656 | *S. sonnei* | NRC | 785,225 | 659,045 | 110,444 | 4,908 | 10,828 |
| S15BD10104 | *S. sonnei* | NRC | 496,927 | 405,143 | 79,687 | 2,792 | 9,305 |
| S15BD10108 | *S. sonnei* | NRC | 609,791 | 494,587 | 99,950 | 3,224 | 12,030 |
| S15BD10196 | *S. sonnei* | NRC | 1,057,861 | 886,099 | 150,585 | 6,955 | 14,222 |
| S15BD10296 | *S. sonnei* | NRC | 456,599 | 390,420 | 54,657 | 4,431 | 7,091 |
| S16BD00285 | *S. sonnei* | NRC | 378,514 | 252,042 | 114,598 | 2,218 | 9,656 |

| | | | | | | | | |
|---|---|---|---|---|---|---|---|---|
| S16BD00582 | *S. sonnei* | NRC | 299,318 | 216,249 | 71,844 | 3,187 | 8,038 |
| S16BD00590 | *S. sonnei* | NRC | 548,940 | 282,132 | 252,213 | 2,299 | 12,296 |
| S16BD00864 | *S. sonnei* | NRC | 196,310 | 113,627 | 75,496 | 908 | 6,279 |
| S16BD01339 | *S. sonnei* | NRC | 468,821 | 283,024 | 173,229 | 2,286 | 10,282 |
| S16BD01740 | *S. sonnei* | NRC | 268,858 | 182,297 | 78,016 | 1,404 | 7,141 |
| S16BD01790 | *S. sonnei* | NRC | 288,894 | 195,810 | 84,560 | 1,433 | 7,091 |
| S16BD02550 | *S. sonnei* | NRC | 134,744 | 80,933 | 48,520 | 683 | 4,608 |
| S16BD02574 | *S. sonnei* | NRC | 280,975 | 190,562 | 80,827 | 1,896 | 7,690 |
| S16BD03340 | *S. sonnei* | NRC | 358,671 | 261,823 | 82,637 | 5,175 | 9,036 |
| S16BD04069 | *S. sonnei* | NRC | 384,392 | 264,517 | 108,723 | 2,187 | 8,965 |
| S17BD01977 | *S. sonnei* | NRC | 258,963 | 227,521 | 26,878 | 2,369 | 2,195 |
| S17BD02088 | *S. sonnei* | NRC | 425,595 | 380,404 | 39,995 | 1,409 | 3,787 |
| S17BD02164 | *S. sonnei* | NRC | 908,385 | 863,828 | 36,792 | 3,770 | 3,995 |
| S17BD02526 | *S. sonnei* | NRC | 910,177 | 853,360 | 48,684 | 3,164 | 4,969 |
| S17BD02897 | *S. sonnei* | NRC | 422,435 | 401,805 | 18,022 | 1,027 | 1,581 |
| S17BD03513 | *S. sonnei* | NRC | 363,205 | 339,574 | 20,375 | 904 | 2,352 |
| S17BD04438 | *S. sonnei* | NRC | 393,957 | 368,457 | 22,037 | 1,205 | 2,258 |
| S17BD06426 | *S. sonnei* | NRC | 441,554 | 407,795 | 29,709 | 1,324 | 2,726 |
| S17BD06464 | *S. sonnei* | NRC | 652,205 | 615,872 | 30,570 | 2,697 | 3,066 |
| S17BD06759 | *S. sonnei* | NRC | 448,112 | 420,690 | 23,920 | 917 | 2,585 |
| S17BD06760 | *S. sonnei* | NRC | 544,251 | 523,754 | 15,916 | 2,683 | 1,898 |
| S17BD06799 | *S. sonnei* | NRC | 526,115 | 497,904 | 24,081 | 1,725 | 2,405 |
| S17BD06801 | *S. sonnei* | NRC | 513,996 | 490,095 | 20,035 | 1,844 | 2,022 |
| S17BD07774 | *S. sonnei* | NRC | 517,048 | 485,712 | 27,342 | 1,629 | 2,365 |
| S17BD07961 | *S. sonnei* | NRC | 454,107 | 425,143 | 24,168 | 2,099 | 2,697 |
| S18BD00011 | *S. sonnei* | NRC | 448,713 | 394,290 | 49,400 | 1,012 | 4,011 |
| S18BD00105 | *S. sonnei* | NRC | 658,681 | 621,584 | 31,137 | 2,437 | 3,523 |
| S18BD00654 | *S. sonnei* | NRC | 423,860 | 392,619 | 26,233 | 2,357 | 2,651 |
| S18BD01122 | *S. sonnei* | NRC | 337,453 | 311,257 | 23,197 | 889 | 2,110 |
| S18BD01231 | *S. sonnei* | NRC | 626,882 | 592,529 | 29,142 | 2,200 | 3,011 |
| S18BD01685 | *S. sonnei* | NRC | 452,961 | 420,698 | 28,382 | 1,273 | 2,608 |
| S18BD01986 | *S. sonnei* | NRC | 602,962 | 566,665 | 30,728 | 2,299 | 3,270 |
| S18BD02200 | *S. sonnei* | NRC | 528,773 | 477,597 | 45,792 | 1,643 | 3,741 |
| ERR586833 | *S. sonnei* | The2018 | 2,515,490 | 2,244,949 | 147,382 | 62,092 | 61,067 |
| ERR586837 | *S. sonnei* | The2018 | 2,189,951 | 1,950,613 | 131,892 | 53,472 | 53,974 |
| ERR586838 | *S. sonnei* | The2018 | 2,393,407 | 2,138,252 | 136,973 | 60,657 | 57,525 |
| ERR591154 | *S. sonnei* | The2018 | 2,843,279 | 2,546,974 | 146,475 | 79,747 | 70,083 |
| ERR591156 | *S. sonnei* | The2018 | 2,400,006 | 2,126,544 | 144,384 | 62,559 | 66,519 |
| ERR591166 | *S. sonnei* | The2018 | 2,546,731 | 2,252,222 | 157,545 | 65,190 | 71,774 |
| ERR591176 | *S. sonnei* | The2018 | 2,857,004 | 2,519,965 | 185,409 | 70,311 | 81,319 |
| ERR591179 | *S. sonnei* | The2018 | 2,424,506 | 2,148,270 | 148,152 | 60,327 | 67,757 |
| ERR591185 | *S. sonnei* | The2018 | 2,234,704 | 1,981,172 | 134,643 | 57,051 | 61,838 |
| ERR591188 | *S. sonnei* | The2018 | 2,260,771 | 2,005,091 | 134,579 | 59,049 | 62,052 |
| ERR591211 | *S. sonnei* | The2018 | 2,536,782 | 2,242,031 | 159,229 | 64,303 | 71,219 |
| ERR591218 | *S. sonnei* | The2018 | 2,490,122 | 2,215,937 | 144,405 | 65,592 | 64,188 |
| ERR591405 | *S. sonnei* | The2018 | 2,686,331 | 2,345,504 | 173,632 | 84,577 | 82,618 |
| ERR592006 | *S. sonnei* | The2018 | 3,177,148 | 2,879,823 | 182,904 | 53,306 | 61,115 |
| ERR592007 | *S. sonnei* | The2018 | 3,355,192 | 3,047,637 | 187,321 | 57,639 | 62,595 |
| ERR592013 | *S. sonnei* | The2018 | 3,097,514 | 2,822,927 | 166,804 | 51,536 | 56,247 |
| ERR592030 | *S. sonnei* | The2018 | 2,366,421 | 2,143,509 | 137,920 | 39,562 | 45,430 |

| | | | | | | | |
|---|---|---|---|---|---|---|---|
| ERR592189 | *S. sonnei* | The2018 | 2,786,632 | 2,550,212 | 134,914 | 50,464 | 51,042 |
| ERR592269 | *S. sonnei* | The2018 | 2,623,122 | 2,395,826 | 125,386 | 52,118 | 49,792 |
| ERR592270 | *S. sonnei* | The2018 | 2,698,438 | 2,448,376 | 141,481 | 53,198 | 55,383 |
| ERR592449 | *S. sonnei* | The2018 | 2,554,629 | 2,333,919 | 127,656 | 44,981 | 48,073 |
| ERR708271 | *S. sonnei* | The2018 | 2,845,475 | 2,668,364 | 116,186 | 30,424 | 30,501 |
| ERR708272 | *S. sonnei* | The2018 | 3,141,792 | 2,946,677 | 128,302 | 33,228 | 33,585 |
| ERR708273 | *S. sonnei* | The2018 | 3,359,737 | 3,144,295 | 142,601 | 35,705 | 37,136 |
| ERR708274 | *S. sonnei* | The2018 | 3,698,759 | 3,479,347 | 141,393 | 40,134 | 37,885 |
| ERR708275 | *S. sonnei* | The2018 | 3,129,616 | 2,937,950 | 125,755 | 33,354 | 32,557 |
| ERR708276 | *S. sonnei* | The2018 | 2,859,000 | 2,686,001 | 113,309 | 30,370 | 29,320 |
| ERR708277 | *S. sonnei* | The2018 | 2,975,200 | 2,804,026 | 108,574 | 33,017 | 29,583 |
| ERR708278 | *S. sonnei* | The2018 | 2,532,889 | 2,378,862 | 100,260 | 27,423 | 26,344 |
| ERR708279 | *S. sonnei* | The2018 | 2,864,072 | 2,678,082 | 124,061 | 29,971 | 31,958 |
| ERR708280 | *S. sonnei* | The2018 | 3,144,444 | 2,952,135 | 126,011 | 33,236 | 33,062 |
| ERR708281 | *S. sonnei* | The2018 | 4,689,142 | 4,423,918 | 167,223 | 51,964 | 46,037 |
| ERR708282 | *S. sonnei* | The2018 | 3,473,655 | 3,248,896 | 148,508 | 37,737 | 38,514 |
| ERR708283 | *S. sonnei* | The2018 | 2,793,343 | 2,627,201 | 106,326 | 30,892 | 28,924 |
| ERR708284 | *S. sonnei* | The2018 | 3,304,315 | 3,074,093 | 160,880 | 36,110 | 33,232 |
| ERR708285 | *S. sonnei* | The2018 | 2,773,112 | 2,605,759 | 108,502 | 30,085 | 28,766 |
| ERR708286 | *S. sonnei* | The2018 | 2,888,694 | 2,714,498 | 112,074 | 31,998 | 30,124 |
| ERR708287 | *S. sonnei* | The2018 | 2,959,999 | 2,778,407 | 118,706 | 31,365 | 31,521 |
| ERR708288 | *S. sonnei* | The2018 | 3,050,857 | 2,858,146 | 127,107 | 32,520 | 33,084 |
| ERR708289 | *S. sonnei* | The2018 | 3,493,993 | 3,280,961 | 138,328 | 37,922 | 36,782 |
| ERR708290 | *S. sonnei* | The2018 | 3,310,247 | 3,101,333 | 136,054 | 36,316 | 36,544 |
| SRR1605255 | *S. sonnei* | The2018 | 988,882 | 883,247 | 74,811 | 12,201 | 18,623 |
| SRR2081185 | *S. sonnei* | The2018 | 1,308,209 | 1,086,759 | 203,633 | 3,249 | 14,568 |
| SRR2081189 | *S. sonnei* | The2018 | 1,761,866 | 1,414,594 | 216,527 | 58,917 | 71,828 |
| SRR2087717 | *S. sonnei* | The2018 | 1,118,891 | 972,638 | 124,408 | 5,826 | 16,019 |
| SRR2097498 | *S. sonnei* | The2018 | 717,185 | 654,973 | 52,390 | 2,978 | 6,844 |
| SRR3473843 | *S. sonnei* | The2018 | 1,960,558 | 1,802,951 | 90,254 | 25,559 | 41,794 |
| SRR3473844 | *S. sonnei* | The2018 | 1,811,141 | 1,634,112 | 114,340 | 21,547 | 41,142 |
| SRR3473847 | *S. sonnei* | The2018 | 1,997,793 | 1,823,128 | 106,521 | 26,184 | 41,960 |
| SRR3473848 | *S. sonnei* | The2018 | 1,987,053 | 1,790,524 | 124,672 | 25,217 | 46,640 |
| SRR3473849 | *S. sonnei* | The2018 | 1,937,324 | 1,745,528 | 119,725 | 25,821 | 46,250 |
| SRR3473850 | *S. sonnei* | The2018 | 1,534,747 | 1,390,394 | 90,785 | 19,578 | 33,990 |
| SRR3473853 | *S. sonnei* | The2018 | 779,223 | 698,619 | 52,583 | 10,104 | 17,917 |
| SRR3473854 | *S. sonnei* | The2018 | 1,147,645 | 1,028,262 | 78,155 | 14,096 | 27,132 |
| SRR3473855 | *S. sonnei* | The2018 | 2,068,619 | 1,877,243 | 122,222 | 24,302 | 44,852 |
| SRR3473856 | *S. sonnei* | The2018 | 1,909,063 | 1,711,237 | 128,603 | 23,017 | 46,206 |
| SRR3473857 | *S. sonnei* | The2018 | 1,981,266 | 1,791,495 | 121,598 | 24,704 | 43,469 |
| SRR3473858 | *S. sonnei* | The2018 | 2,012,154 | 1,830,571 | 111,844 | 25,568 | 44,171 |
| SRR3473859 | *S. sonnei* | The2018 | 1,973,415 | 1,788,360 | 115,059 | 25,163 | 44,833 |
| SRR3473860 | *S. sonnei* | The2018 | 1,932,917 | 1,764,462 | 103,911 | 24,048 | 40,496 |
| SRR3473861 | *S. sonnei* | The2018 | 1,835,917 | 1,690,450 | 81,583 | 25,293 | 38,591 |
| SRR3473862 | *S. sonnei* | The2018 | 1,987,226 | 1,813,516 | 105,196 | 25,246 | 43,268 |
| SRR3474162 | *S. sonnei* | The2018 | 479,422 | 415,637 | 52,799 | 7,034 | 3,952 |
| SRR3474163 | *S. sonnei* | The2018 | 414,025 | 329,387 | 73,358 | 6,453 | 4,827 |
| SRR3474164 | *S. sonnei* | The2018 | 492,007 | 426,721 | 53,862 | 5,945 | 5,479 |
| SRR3474165 | *S. sonnei* | The2018 | 1,071,143 | 836,073 | 214,723 | 5,115 | 15,232 |
| SRR3474166 | *S. sonnei* | The2018 | 494,173 | 380,231 | 104,468 | 2,433 | 7,041 |

| | | | | | | | |
|---|---|---|---|---|---|---|---|
| SRR3474167 | *S. sonnei* | The2018 | 599,630 | 466,939 | 120,505 | 3,082 | 9,104 |
| SRR3474168 | *S. sonnei* | The2018 | 417,263 | 322,748 | 86,371 | 2,012 | 6,132 |
| SRR3474169 | *S. sonnei* | The2018 | 330,877 | 293,918 | 28,556 | 2,237 | 6,166 |
| SRR3474170 | *S. sonnei* | The2018 | 574,489 | 460,903 | 100,189 | 2,798 | 10,599 |
| SRR3474171 | *S. sonnei* | The2018 | 511,317 | 402,515 | 95,310 | 7,109 | 6,383 |
| SRR3474172 | *S. sonnei* | The2018 | 659,862 | 537,077 | 103,690 | 4,013 | 15,082 |

**Supplementary Table S3: Assembly and coverage statistics.**

| Sample | Species | Collection | No. of contigs | N50 | Total assembly length | Coverage | Read mapping back to assembly (%) |
|---|---|---|---|---|---|---|---|
| ERR042796 | *S. flexneri* | Connor2015 | 280 | 30,078 | 4,363,070 | 93 | 96.18 |
| ERR042797 | *S. flexneri* | Connor2015 | 270 | 32,663 | 4,464,046 | 87 | 94.6 |
| ERR042799 | *S. flexneri* | Connor2015 | 270 | 29,911 | 4,246,440 | 128 | 95.53 |
| ERR042803 | *S. flexneri* | Connor2015 | 280 | 30,464 | 4,365,484 | 122 | 95.51 |
| ERR042806 | *S. flexneri* | Connor2015 | 270 | 29,914 | 4,232,304 | 97 | 94.47 |
| ERR042810 | *S. flexneri* | Connor2015 | 244 | 32,747 | 4,132,148 | 387 | 96.88 |
| ERR042811 | *S. flexneri* | Connor2015 | 309 | 27,268 | 4,334,080 | 101 | 96.2 |
| ERR042814 | *S. flexneri* | Connor2015 | 285 | 31,339 | 4,418,275 | 104 | 94.93 |
| ERR042816 | *S. flexneri* | Connor2015 | 278 | 31,305 | 4,366,718 | 97 | 95.27 |
| ERR042819 | *S. flexneri* | Connor2015 | 293 | 27,648 | 4,259,539 | 101 | 96.07 |
| ERR042821 | *S. flexneri* | Connor2015 | 275 | 30,408 | 4,294,042 | 93 | 96.24 |
| ERR042824 | *S. flexneri* | Connor2015 | 284 | 31,339 | 4,401,942 | 93 | 94.72 |
| ERR042825 | *S. flexneri* | Connor2015 | 284 | 29,936 | 4,336,371 | 95 | 94.39 |
| ERR042827 | *S. flexneri* | Connor2015 | 313 | 25,603 | 4,216,926 | 114 | 95.95 |
| ERR042831 | *S. flexneri* | Connor2015 | 270 | 31,921 | 4,290,133 | 111 | 93.88 |
| ERR042832 | *S. flexneri* | Connor2015 | 308 | 30,104 | 4,421,168 | 145 | 95.02 |
| ERR042833 | *S. flexneri* | Connor2015 | 309 | 28,275 | 4,207,913 | 152 | 95.74 |
| ERR042835 | *S. flexneri* | Connor2015 | 233 | 32,257 | 4,042,292 | 116 | 96.03 |
| ERR042837 | *S. flexneri* | Connor2015 | 294 | 29,294 | 4,253,083 | 116 | 95.94 |
| ERR042838 | *S. flexneri* | Connor2015 | 322 | 24,983 | 4,259,241 | 120 | 95.54 |
| ERR042839 | *S. flexneri* | Connor2015 | 284 | 31,318 | 4,374,422 | 111 | 95.55 |
| ERR042840 | *S. flexneri* | Connor2015 | 278 | 30,187 | 4,327,989 | 117 | 95.11 |
| ERR042841 | *S. flexneri* | Connor2015 | 294 | 30,961 | 4,418,406 | 126 | 94.48 |
| ERR042842 | *S. flexneri* | Connor2015 | 283 | 31,305 | 4,383,811 | 177 | 94.6 |
| ERR042843 | *S. flexneri* | Connor2015 | 299 | 33,474 | 4,646,460 | 111 | 95.41 |
| ERR042845 | *S. flexneri* | Connor2015 | 289 | 29,915 | 4,347,196 | 118 | 95.24 |
| ERR042849 | *S. flexneri* | Connor2015 | 258 | 31,307 | 4,217,188 | 118 | 95.06 |
| ERR042851 | *S. flexneri* | Connor2015 | 293 | 31,441 | 4,395,552 | 111 | 94.69 |
| ERR042852 | *S. flexneri* | Connor2015 | 269 | 33,654 | 4,327,921 | 106 | 95.51 |
| ERR042853 | *S. flexneri* | Connor2015 | 263 | 30,022 | 4,191,789 | 140 | 95.02 |
| ERR042855 | *S. flexneri* | Connor2015 | 302 | 27,853 | 4,360,653 | 107 | 93.98 |
| ERR042858 | *S. flexneri* | Connor2015 | 342 | 25,257 | 4,495,043 | 108 | 95.59 |
| ERR042860 | *S. flexneri* | Connor2015 | 292 | 31,340 | 4,450,387 | 105 | 94.08 |
| ERR042861 | *S. flexneri* | Connor2015 | 294 | 31,305 | 4,352,174 | 98 | 95.64 |
| ERR042863 | *S. flexneri* | Connor2015 | 285 | 29,915 | 4,326,210 | 92 | 93.97 |
| ERR047236 | *S. flexneri* | Connor2015 | 269 | 31,307 | 4,258,158 | 103 | 95.56 |
| ERR047239 | *S. flexneri* | Connor2015 | 315 | 26,003 | 4,392,421 | 114 | 93.75 |
| ERR047294 | *S. flexneri* | Connor2015 | 291 | 31,307 | 4,380,849 | 136 | 95.37 |
| ERR047297 | *S. flexneri* | Connor2015 | 326 | 25,169 | 4,391,585 | 136 | 95.49 |
| ERR047306 | *S. flexneri* | Connor2015 | 299 | 30,056 | 4,468,018 | 65 | 95.07 |
| ERR047307 | *S. flexneri* | Connor2015 | 323 | 27,030 | 4,494,017 | 172 | 95.27 |
| ERR047372 | *S. flexneri* | Connor2015 | 325 | 25,117 | 4,389,712 | 111 | 94.56 |
| ERR047396 | *S. flexneri* | Connor2015 | 310 | 25,160 | 4,345,007 | 224 | 93.26 |
| ERR047406 | *S. flexneri* | Connor2015 | 303 | 29,338 | 4,445,536 | 54 | 95.24 |

| | | | | | | | |
|---|---|---|---|---|---|---|---|
| ERR048234 | *S. flexneri* | Connor2015 | 259 | 31,332 | 4,245,936 | 136 | 95.95 |
| ERR048246 | *S. flexneri* | Connor2015 | 261 | 31,305 | 4,257,964 | 178 | 95.26 |
| ERR048259 | *S. flexneri* | Connor2015 | 270 | 35,351 | 4,633,378 | 152 | 94.92 |
| ERR048261 | *S. flexneri* | Connor2015 | 281 | 31,882 | 4,266,145 | 46 | 95.91 |
| ERR048265 | *S. flexneri* | Connor2015 | 264 | 31,935 | 4,334,532 | 153 | 95.79 |
| ERR048285 | *S. flexneri* | Connor2015 | 280 | 34,340 | 4,602,348 | 138 | 90.19 |
| ERR048286 | *S. flexneri* | Connor2015 | 241 | 32,241 | 4,123,978 | 137 | 96.25 |
| ERR048287 | *S. flexneri* | Connor2015 | 271 | 30,943 | 4,334,835 | 117 | 89.22 |
| ERR048288 | *S. flexneri* | Connor2015 | 297 | 29,177 | 4,197,391 | 131 | 93.68 |
| ERR048290 | *S. flexneri* | Connor2015 | 262 | 32,242 | 4,207,666 | 113 | 93.91 |
| ERR048295 | *S. flexneri* | Connor2015 | 288 | 31,305 | 4,412,890 | 158 | 93.85 |
| ERR048296 | *S. flexneri* | Connor2015 | 313 | 28,574 | 4,401,221 | 136 | 92.53 |
| ERR048300 | *S. flexneri* | Connor2015 | 274 | 30,965 | 4,523,402 | 162 | 95.35 |
| ERR048302 | *S. flexneri* | Connor2015 | 268 | 33,631 | 4,352,621 | 48 | 92.19 |
| ERR048304 | *S. flexneri* | Connor2015 | 279 | 29,931 | 4,280,064 | 130 | 89.14 |
| ERR048306 | *S. flexneri* | Connor2015 | 269 | 29,911 | 4,272,087 | 154 | 95.07 |
| ERR048311 | *S. flexneri* | Connor2015 | 315 | 25,628 | 4,271,855 | 165 | 95.98 |
| ERR048312 | *S. flexneri* | Connor2015 | 337 | 24,924 | 4,538,806 | 151 | 95.47 |
| ERR048313 | *S. flexneri* | Connor2015 | 297 | 33,647 | 4,426,996 | 158 | 94.78 |
| ERR048315 | *S. flexneri* | Connor2015 | 323 | 29,579 | 4,396,268 | 31 | 91.63 |
| ERR048316 | *S. flexneri* | Connor2015 | 251 | 32,747 | 4,154,080 | 98 | 93.56 |
| ERR048317 | *S. flexneri* | Connor2015 | 291 | 25,988 | 4,217,306 | 94 | 95.35 |
| ERR048319 | *S. flexneri* | Connor2015 | 285 | 31,305 | 4,411,213 | 109 | 92.02 |
| ERR048320 | *S. flexneri* | Connor2015 | 295 | 30,778 | 4,425,013 | 165 | 94.25 |
| ERR048322 | *S. flexneri* | Connor2015 | 295 | 31,305 | 4,460,495 | 87 | 94.1 |
| ERR048329 | *S. flexneri* | Connor2015 | 328 | 26,743 | 4,445,963 | 115 | 95.23 |
| ERR048331 | *S. flexneri* | Connor2015 | 285 | 33,406 | 4,397,532 | 127 | 94.66 |
| ERR048332 | *S. flexneri* | Connor2015 | 320 | 28,641 | 4,449,817 | 150 | 94.79 |
| ERR048333 | *S. flexneri* | Connor2015 | 304 | 26,175 | 4,284,766 | 212 | 96.05 |
| ERR048334 | *S. flexneri* | Connor2015 | 316 | 25,487 | 4,334,646 | 159 | 94.18 |
| ERR048339 | *S. flexneri* | Connor2015 | 291 | 30,639 | 4,413,124 | 199 | 94.1 |
| ERR049152 | *S. flexneri* | Connor2015 | 321 | 26,002 | 4,411,985 | 411 | 93.19 |
| ERR126958 | *S. flexneri* | Connor2015 | 887 | 6,609 | 3,808,848 | 98 | 82.84 |
| ERR127015 | *S. flexneri* | Connor2015 | 330 | 27,649 | 4,344,130 | 123 | 94.22 |
| ERR127017 | *S. flexneri* | Connor2015 | 779 | 8,390 | 4,173,256 | 102 | 89.77 |
| ERR127019 | *S. flexneri* | Connor2015 | 450 | 15,037 | 4,207,014 | 109 | 94 |
| ERR127032 | *S. flexneri* | Connor2015 | 458 | 15,590 | 4,208,626 | 113 | 94.44 |
| ERR127034 | *S. flexneri* | Connor2015 | 352 | 20,815 | 4,139,649 | 116 | 94.65 |
| ERR127035 | *S. flexneri* | Connor2015 | 553 | 15,086 | 3,976,579 | 112 | 92.49 |
| ERR127036 | *S. flexneri* | Connor2015 | 713 | 10,531 | 4,025,031 | 101 | 92.29 |
| ERR127037 | *S. flexneri* | Connor2015 | 254 | 34,494 | 4,500,191 | 93 | 94.22 |
| ERR127038 | *S. flexneri* | Connor2015 | 740 | 11,720 | 3,811,652 | 104 | 85.61 |
| ERR127039 | *S. flexneri* | Connor2015 | 544 | 15,122 | 4,253,383 | 115 | 92.84 |
| ERR127040 | *S. flexneri* | Connor2015 | 549 | 14,966 | 3,992,922 | 110 | 93.92 |
| ERR127041 | *S. flexneri* | Connor2015 | 226 | 35,033 | 4,123,706 | 109 | 96.48 |
| ERR127042 | *S. flexneri* | Connor2015 | 531 | 15,533 | 4,162,037 | 111 | 93.61 |
| ERR127043 | *S. flexneri* | Connor2015 | 570 | 13,623 | 4,153,572 | 111 | 93.72 |
| ERR127044 | *S. flexneri* | Connor2015 | 704 | 10,387 | 3,849,672 | 103 | 88.84 |
| ERR127046 | *S. flexneri* | Connor2015 | 232 | 37,342 | 4,237,944 | 317 | 94.99 |
| ERR127047 | *S. flexneri* | Connor2015 | 247 | 33,512 | 4,216,248 | 356 | 95.22 |

| | | | | | | | |
|---|---|---|---|---|---|---|---|
| ERR127048 | *S. flexneri* | Connor2015 | 252 | 31,929 | 4,270,226 | 430 | 95.87 |
| ERR200344 | *S. flexneri* | Connor2015 | 265 | 30,531 | 4,194,471 | 65 | 96.06 |
| ERR200360 | *S. flexneri* | Connor2015 | 285 | 31,340 | 4,392,980 | 76 | 92.97 |
| ERR200365 | *S. flexneri* | Connor2015 | 246 | 33,352 | 4,184,341 | 86 | 94.9 |
| ERR200370 | *S. flexneri* | Connor2015 | 292 | 31,305 | 4,385,568 | 80 | 94.85 |
| ERR200378 | *S. flexneri* | Connor2015 | 280 | 31,340 | 4,340,667 | 79 | 94.83 |
| ERR200379 | *S. flexneri* | Connor2015 | 288 | 31,340 | 4,373,123 | 45 | 93.57 |
| ERR200390 | *S. flexneri* | Connor2015 | 285 | 33,259 | 4,402,829 | 70 | 93.98 |
| ERR200392 | *S. flexneri* | Connor2015 | 285 | 30,639 | 4,402,529 | 88 | 93.62 |
| ERR200393 | *S. flexneri* | Connor2015 | 310 | 24,693 | 4,339,529 | 48 | 94.19 |
| ERR200402 | *S. flexneri* | Connor2015 | 285 | 25,560 | 4,169,444 | 66 | 92.44 |
| ERR200403 | *S. flexneri* | Connor2015 | 312 | 25,197 | 4,316,354 | 48 | 94.65 |
| ERR200405 | *S. flexneri* | Connor2015 | 326 | 24,576 | 4,373,392 | 56 | 93.3 |
| ERR200413 | *S. flexneri* | Connor2015 | 257 | 28,983 | 4,287,947 | 61 | 94.99 |
| ERR200414 | *S. flexneri* | Connor2015 | 309 | 27,683 | 4,219,354 | 43 | 91.64 |
| ERR200415 | *S. flexneri* | Connor2015 | 318 | 25,899 | 4,452,082 | 85 | 94.23 |
| ERR217012 | *S. flexneri* | Connor2015 | 307 | 28,274 | 4,218,607 | 63 | 95.48 |
| ERR217013 | *S. flexneri* | Connor2015 | 417 | 17,590 | 4,198,531 | 48 | 92.91 |
| ERR217015 | *S. flexneri* | Connor2015 | 313 | 28,057 | 4,226,549 | 74 | 93.74 |
| ERR217016 | *S. flexneri* | Connor2015 | 305 | 28,274 | 4,251,488 | 82 | 94.76 |
| ERR217022 | *S. flexneri* | Connor2015 | 298 | 28,274 | 4,154,675 | 57 | 92.98 |
| ERR217023 | *S. flexneri* | Connor2015 | 312 | 28,182 | 4,237,179 | 57 | 92.83 |
| ERR217024 | *S. flexneri* | Connor2015 | 303 | 28,274 | 4,225,858 | 49 | 93.39 |
| ERR217026 | *S. flexneri* | Connor2015 | 328 | 28,273 | 4,312,990 | 55 | 93.07 |
| ERR217028 | *S. flexneri* | Connor2015 | 315 | 28,276 | 4,309,645 | 43 | 92.22 |
| ERR217030 | *S. flexneri* | Connor2015 | 309 | 28,057 | 4,226,134 | 41 | 93.98 |
| ERR217031 | *S. flexneri* | Connor2015 | 306 | 28,752 | 4,216,250 | 62 | 93.06 |
| ERR217032 | *S. flexneri* | Connor2015 | 307 | 28,185 | 4,213,326 | 36 | 94.29 |
| ERR217033 | *S. flexneri* | Connor2015 | 314 | 31,332 | 4,355,693 | 75 | 93.47 |
| ERR217047 | *S. flexneri* | Connor2015 | 283 | 30,007 | 4,330,260 | 43 | 94.14 |
| ERR217080 | *S. flexneri* | Connor2015 | 265 | 31,307 | 4,200,552 | 168 | 95.49 |
| ERR217081 | *S. flexneri* | Connor2015 | 295 | 29,897 | 4,447,124 | 56 | 95.9 |
| ERR217084 | *S. flexneri* | Connor2015 | 290 | 32,254 | 4,445,107 | 62 | 93.8 |
| ERR559526 | *S. flexneri* | Connor2015 | 243 | 33,297 | 4,175,298 | 71 | 95.53 |
| ERR832453 | *S. flexneri* | Connor2015 | 25 | 2,511 | 54,995 | 224 | 5.42 |
| ERR832456 | *S. flexneri* | Connor2015 | 10 | 1,826 | 17,418 | 424.5 | 2.85 |
| ERR832457 | *S. flexneri* | Connor2015 | 19 | 1,703 | 33,057 | 267 | 5.27 |
| ERR832459 | *S. flexneri* | Connor2015 | 32 | 1,949 | 55,174 | 207 | 9.16 |
| ERR832460 | *S. flexneri* | Connor2015 | 25 | 2,122 | 56,507 | 247 | 5.39 |
| ERR832461 | *S. flexneri* | Connor2015 | 25 | 10,847 | 129,465 | 224 | 9.06 |
| ERR832462 | *S. flexneri* | Connor2015 | 32 | 5,560 | 139,886 | 272 | 13.63 |
| ERR832464 | *S. flexneri* | Connor2015 | 17 | 1,658 | 28,868 | 333 | 5.63 |
| ERR832465 | *S. flexneri* | Connor2015 | 279 | 28,578 | 4,197,232 | 88 | 94.04 |
| ERR832467 | *S. flexneri* | Connor2015 | 15 | 1,890 | 28,196 | 482 | 9.06 |
| ERR832468 | *S. flexneri* | Connor2015 | 34 | 1,950 | 60,880 | 193 | 7.92 |
| ERR832470 | *S. flexneri* | Connor2015 | 21 | 2,481 | 53,870 | 271 | 5.39 |
| ERR832471 | *S. flexneri* | Connor2015 | 20 | 2,085 | 40,802 | 634 | 10.64 |
| ERR832473 | *S. flexneri* | Connor2015 | 24 | 5,772 | 85,569 | 356 | 10.53 |
| ERR832474 | *S. flexneri* | Connor2015 | 43 | 12,962 | 193,934 | 325 | 13.89 |
| ERR832477 | *S. flexneri* | Connor2015 | 21 | 5,273 | 63,169 | 506 | 14.76 |

| | | | | | | | | |
|---|---|---|---|---|---|---|---|---|
| ERR832480 | *S. flexneri* | Connor2015 | 18 | 1,874 | 34,289 | 300 | 4.26 |
| ERR832481 | *S. flexneri* | Connor2015 | 36 | 1,902 | 66,213 | 195 | 6.28 |
| ERR832483 | *S. flexneri* | Connor2015 | 23 | 1,818 | 42,261 | 260 | 5.82 |
| ERR832485 | *S. flexneri* | Connor2015 | 36 | 1,939 | 68,025 | 212 | 7.17 |
| ERR832486 | *S. flexneri* | Connor2015 | 16 | 1,604 | 28,281 | 292 | 4.02 |
| ERR832487 | *S. flexneri* | Connor2015 | 36 | 31,819 | 330,617 | 256 | 24.75 |
| ERR832489 | *S. flexneri* | Connor2015 | 11 | 2,224 | 21,374 | 462 | 3.44 |
| ERR832490 | *S. flexneri* | Connor2015 | 30 | 3,776 | 79,050 | 226 | 6.56 |
| ERR832491 | *S. flexneri* | Connor2015 | 16 | 1,597 | 26,826 | 346 | 3.71 |
| ERR832492 | *S. flexneri* | Connor2015 | 15 | 4,121 | 36,423 | 247 | 3.58 |
| ERR832494 | *S. flexneri* | Connor2015 | 23 | 1,604 | 40,268 | 293 | 5.17 |
| S13BD00302 | *S. flexneri* | NRC | 246 | 33,479 | 4,280,656 | 39 | 96.16 |
| S13BD00535 | *S. flexneri* | NRC | 295 | 30,204 | 4,535,548 | 18 | 96.86 |
| S13BD00702 | *S. flexneri* | NRC | 281 | 33,791 | 4,470,836 | 55 | 96.71 |
| S13BD00854 | *S. flexneri* | NRC | 289 | 34,724 | 4,556,789 | 54 | 95.79 |
| S13BD01310 | *S. flexneri* | NRC | 304 | 26,204 | 4,433,403 | 53 | 97.11 |
| S13BD01692 | *S. flexneri* | NRC | 277 | 35,226 | 4,449,950 | 43 | 97.02 |
| S13BD02195 | *S. flexneri* | NRC | 279 | 33,791 | 4,472,575 | 37 | 96.47 |
| S13BD02470 | *S. flexneri* | NRC | 303 | 27,983 | 4,491,985 | 43 | 96.31 |
| S13BD03084 | *S. flexneri* | NRC | 284 | 32,733 | 4,464,907 | 63 | 94.06 |
| S13BD04017 | *S. flexneri* | NRC | 309 | 29,344 | 4,478,335 | 45 | 91.21 |
| S13BD04231 | *S. flexneri* | NRC | 304 | 29,712 | 4,459,125 | 27 | 95.11 |
| S14BD00799 | *S. flexneri* | NRC | 288 | 33,916 | 4,465,922 | 73 | 97.46 |
| S14BD01131 | *S. flexneri* | NRC | 314 | 29,993 | 4,488,076 | 40 | 85.86 |
| S14BD01142 | *S. flexneri* | NRC | 316 | 32,053 | 4,583,539 | 73 | 86.31 |
| S14BD01714 | *S. flexneri* | NRC | 284 | 29,993 | 4,382,968 | 46 | 95.09 |
| S14BD02502 | *S. flexneri* | NRC | 298 | 29,993 | 4,437,739 | 55 | 95.91 |
| S14BD02795 | *S. flexneri* | NRC | 1220 | 11,684 | 8,242,670 | 39 | 93.99 |
| S14BD05234 | *S. flexneri* | NRC | 307 | 28,695 | 4,361,308 | 16 | 97.22 |
| S14BD05285 | *S. flexneri* | NRC | 282 | 33,446 | 4,451,825 | 27 | 95.71 |
| S15BD00055 | *S. flexneri* | NRC | 283 | 33,937 | 4,466,605 | 22 | 95.57 |
| S15BD00457 | *S. flexneri* | NRC | 283 | 34,798 | 4,514,435 | 40 | 96.24 |
| S15BD00494 | *S. flexneri* | NRC | 281 | 34,930 | 4,454,586 | 39 | 95.52 |
| S15BD02368 | *S. flexneri* | NRC | 287 | 33,436 | 4,477,291 | 41 | 95.85 |
| S15BD02726 | *S. flexneri* | NRC | 304 | 31,449 | 4,521,065 | 47 | 95.54 |
| S15BD02867 | *S. flexneri* | NRC | 275 | 35,045 | 4,462,944 | 58 | 96.77 |
| S15BD04659 | *S. flexneri* | NRC | 277 | 34,930 | 4,445,927 | 41 | 96.44 |
| S15BD05228 | *S. flexneri* | NRC | 280 | 33,941 | 4,464,084 | 43 | 95.87 |
| S15BD06353 | *S. flexneri* | NRC | 289 | 32,050 | 4,447,991 | 38 | 95.39 |
| S15BD06515 | *S. flexneri* | NRC | 704 | 2,992 | 1,885,243 | 10 | 51.23 |
| S15BD08204 | *S. flexneri* | NRC | 290 | 32,305 | 4,433,013 | 51 | 94.37 |
| S15BD08762 | *S. flexneri* | NRC | 284 | 31,447 | 4,464,855 | 55 | 96.88 |
| S15BD09335 | *S. flexneri* | NRC | 289 | 29,993 | 4,320,055 | 65 | 94.71 |
| S15BD09453 | *S. flexneri* | NRC | 277 | 30,158 | 4,421,887 | 43 | 95.68 |
| S15BD09529 | *S. flexneri* | NRC | 993 | 3,112 | 2,689,126 | 9 | 67.51 |
| S16BD00148 | *S. flexneri* | NRC | 294 | 30,794 | 4,555,114 | 23 | 95.84 |
| S16BD00463 | *S. flexneri* | NRC | 366 | 22,130 | 4,358,138 | 15 | 96.43 |
| S16BD01789 | *S. flexneri* | NRC | 282 | 35,188 | 4,437,260 | 33 | 96.81 |
| S16BD01991 | *S. flexneri* | NRC | 286 | 32,891 | 4,520,892 | 62 | 96.31 |
| S16BD02637 | *S. flexneri* | NRC | 290 | 29,993 | 4,417,941 | 40 | 95.67 |

| | | | | | | | |
|---|---|---|---|---|---|---|---|
| S16BD02856 | *S. flexneri* | NRC | 287 | 32,949 | 4,531,862 | 37 | 95.79 |
| S16BD03590 | *S. flexneri* | NRC | 366 | 21,307 | 4,272,284 | 19 | 96.26 |
| S16BD03881 | *S. flexneri* | NRC | 354 | 25,930 | 4,424,040 | 17 | 95.41 |
| S16BD04652 | *S. flexneri* | NRC | 275 | 33,528 | 4,393,181 | 47 | 97.27 |
| S16BD07706 | *S. flexneri* | NRC | 293 | 29,674 | 4,402,397 | 33 | 96.46 |
| S17BD00498 | *S. flexneri* | NRC | 296 | 33,937 | 4,464,897 | 29 | 87.41 |
| S17BD00886 | *S. flexneri* | NRC | 287 | 34,721 | 4,461,897 | 33 | 90.05 |
| S17BD02190 | *S. flexneri* | NRC | 293 | 34,721 | 4,464,827 | 24 | 77.89 |
| S17BD02300 | *S. flexneri* | NRC | 297 | 30,584 | 4,473,289 | 21 | 83.88 |
| S17BD03514 | *S. flexneri* | NRC | 287 | 33,941 | 4,562,089 | 31 | 96.69 |
| S17BD04033 | *S. flexneri* | NRC | 345 | 25,192 | 4,476,084 | 46 | 94.79 |
| S17BD04223 | *S. flexneri* | NRC | 296 | 30,922 | 4,470,022 | 26 | 89.99 |
| S17BD04752 | *S. flexneri* | NRC | 279 | 34,839 | 4,475,256 | 38 | 96.34 |
| S17BD06364 | *S. flexneri* | NRC | 287 | 29,813 | 4,328,397 | 53 | 95.73 |
| S17BD07654 | *S. flexneri* | NRC | 270 | 34,722 | 4,616,533 | 50 | 95.23 |
| S17BD08054 | *S. flexneri* | NRC | 326 | 29,301 | 4,548,834 | 18 | 73.81 |
| S17BD08194 | *S. flexneri* | NRC | 290 | 31,916 | 4,467,206 | 21 | 86.38 |
| S17BD08237 | *S. flexneri* | NRC | 297 | 30,555 | 4,453,255 | 24 | 83.33 |
| S18BD00005 | *S. flexneri* | NRC | 300 | 34,696 | 4,540,246 | 25 | 85.75 |
| S18BD00006 | *S. flexneri* | NRC | 302 | 32,440 | 4,494,784 | 19 | 84.67 |
| S18BD01109 | *S. flexneri* | NRC | 329 | 28,585 | 4,458,199 | 25 | 94.85 |
| S18BD01126 | *S. flexneri* | NRC | 333 | 28,592 | 4,538,768 | 14 | 75.41 |
| S18BD01471 | *S. flexneri* | NRC | 290 | 33,791 | 4,461,539 | 18 | 81.53 |
| ERR316291 | *S. sonnei* | Baker2017 | 339 | 24,902 | 4,604,374 | 46 | 85.49 |
| ERR316299 | *S. sonnei* | Baker2017 | 321 | 25,218 | 4,442,752 | 84 | 93.67 |
| ERR316306 | *S. sonnei* | Baker2017 | 304 | 26,481 | 4,547,993 | 83 | 94.5 |
| ERR024604 | *S. sonnei* | Holt2012 | 336 | 23,584 | 4,467,120 | 22 | 91.25 |
| ERR024605 | *S. sonnei* | Holt2012 | 326 | 23,828 | 4,434,926 | 64 | 93.04 |
| ERR024606 | *S. sonnei* | Holt2012 | 340 | 25,029 | 4,601,354 | 68 | 91.86 |
| ERR024607 | *S. sonnei* | Holt2012 | 333 | 23,799 | 4,466,878 | 49 | 92.81 |
| ERR024608 | *S. sonnei* | Holt2012 | 363 | 22,202 | 4,369,978 | 74 | 91.46 |
| ERR024609 | *S. sonnei* | Holt2012 | 340 | 22,301 | 4,297,692 | 72 | 92.33 |
| ERR024610 | *S. sonnei* | Holt2012 | 348 | 21,714 | 4,305,511 | 48 | 93.18 |
| ERR024611 | *S. sonnei* | Holt2012 | 372 | 21,260 | 4,294,978 | 41 | 92.88 |
| ERR024612 | *S. sonnei* | Holt2012 | 345 | 22,202 | 4,279,738 | 44 | 92.62 |
| ERR024616 | *S. sonnei* | Holt2012 | 369 | 21,714 | 4,487,084 | 45 | 91.68 |
| ERR024617 | *S. sonnei* | Holt2012 | 361 | 21,623 | 4,421,280 | 51 | 92.11 |
| ERR024618 | *S. sonnei* | Holt2012 | 391 | 21,260 | 4,634,542 | 41 | 91 |
| ERR024619 | *S. sonnei* | Holt2012 | 343 | 23,587 | 4,553,854 | 32 | 93.15 |
| ERR024620 | *S. sonnei* | Holt2012 | 354 | 21,261 | 4,423,597 | 43 | 90.94 |
| ERR024621 | *S. sonnei* | Holt2012 | 351 | 24,708 | 4,606,375 | 47 | 93.39 |
| ERR024622 | *S. sonnei* | Holt2012 | 347 | 21,260 | 4,312,900 | 53 | 92.08 |
| ERR024625 | *S. sonnei* | Holt2012 | 356 | 21,988 | 4,426,095 | 36 | 90.94 |
| ERR024626 | *S. sonnei* | Holt2012 | 362 | 22,306 | 4,422,017 | 42 | 91.59 |
| ERR024627 | *S. sonnei* | Holt2012 | 348 | 21,261 | 4,290,647 | 44 | 92.76 |
| ERR025682 | *S. sonnei* | Holt2012 | 375 | 21,360 | 4,505,252 | 26 | 58.09 |
| ERR025683 | *S. sonnei* | Holt2012 | 403 | 20,301 | 4,432,276 | 19 | 50.12 |
| ERR025685 | *S. sonnei* | Holt2012 | 377 | 21,623 | 4,390,792 | 46 | 79.43 |
| ERR025686 | *S. sonnei* | Holt2012 | 346 | 21,715 | 4,514,405 | 38 | 68.53 |
| ERR025687 | *S. sonnei* | Holt2012 | 388 | 21,916 | 4,658,506 | 22 | 67.48 |

| | | | | | | | |
|---|---|---|---|---|---|---|---|
| ERR025689 | *S. sonnei* | Holt2012 | 380 | 20,644 | 4,439,803 | 15 | 59.46 |
| ERR025690 | *S. sonnei* | Holt2012 | 447 | 19,443 | 4,588,698 | 21 | 66.94 |
| ERR025691 | *S. sonnei* | Holt2012 | 364 | 23,828 | 4,596,471 | 31 | 67.32 |
| ERR025692 | *S. sonnei* | Holt2012 | 336 | 22,263 | 4,429,736 | 29 | 57.16 |
| ERR025693 | *S. sonnei* | Holt2012 | 374 | 22,307 | 4,617,496 | 33 | 67.33 |
| ERR025695 | *S. sonnei* | Holt2012 | 398 | 18,585 | 4,242,790 | 19 | 66.42 |
| ERR025697 | *S. sonnei* | Holt2012 | 407 | 19,585 | 4,304,350 | 16 | 52.25 |
| ERR025698 | *S. sonnei* | Holt2012 | 374 | 22,204 | 4,476,972 | 49 | 82.92 |
| ERR025699 | *S. sonnei* | Holt2012 | 390 | 21,230 | 4,443,344 | 40 | 69.93 |
| ERR025700 | *S. sonnei* | Holt2012 | 439 | 19,571 | 4,445,553 | 16 | 53.58 |
| ERR025701 | *S. sonnei* | Holt2012 | 365 | 23,225 | 4,377,480 | 32 | 63.11 |
| ERR025702 | *S. sonnei* | Holt2012 | 411 | 17,467 | 4,228,856 | 13 | 40.28 |
| ERR025703 | *S. sonnei* | Holt2012 | 381 | 19,476 | 4,319,339 | 35 | 77.84 |
| ERR025704 | *S. sonnei* | Holt2012 | 381 | 21,714 | 4,436,896 | 27 | 72.97 |
| ERR025705 | *S. sonnei* | Holt2012 | 384 | 22,065 | 4,411,598 | 30 | 65.82 |
| ERR025706 | *S. sonnei* | Holt2012 | 350 | 23,303 | 4,318,812 | 24 | 58.23 |
| ERR025708 | *S. sonnei* | Holt2012 | 410 | 19,137 | 4,317,099 | 16 | 72.42 |
| ERR025709 | *S. sonnei* | Holt2012 | 390 | 21,260 | 4,419,699 | 24 | 65.3 |
| ERR025710 | *S. sonnei* | Holt2012 | 377 | 21,260 | 4,447,916 | 32 | 71.13 |
| ERR025711 | *S. sonnei* | Holt2012 | 385 | 21,145 | 4,435,149 | 64 | 85.47 |
| ERR025712 | *S. sonnei* | Holt2012 | 368 | 23,225 | 4,439,913 | 54 | 79.37 |
| ERR025713 | *S. sonnei* | Holt2012 | 382 | 22,305 | 4,506,047 | 48 | 77.48 |
| ERR025714 | *S. sonnei* | Holt2012 | 342 | 22,202 | 4,289,994 | 31 | 68.22 |
| ERR025715 | *S. sonnei* | Holt2012 | 337 | 23,769 | 4,514,442 | 22 | 60.76 |
| ERR025716 | *S. sonnei* | Holt2012 | 386 | 22,065 | 4,432,393 | 55 | 86.03 |
| ERR025717 | *S. sonnei* | Holt2012 | 381 | 22,092 | 4,444,194 | 42 | 80.25 |
| ERR025718 | *S. sonnei* | Holt2012 | 373 | 21,714 | 4,437,441 | 48 | 77.74 |
| ERR025719 | *S. sonnei* | Holt2012 | 346 | 22,659 | 4,332,827 | 45 | 74.07 |
| ERR025721 | *S. sonnei* | Holt2012 | 420 | 17,750 | 4,355,691 | 20 | 49.35 |
| ERR025724 | *S. sonnei* | Holt2012 | 329 | 24,023 | 4,389,419 | 46 | 78.47 |
| ERR025725 | *S. sonnei* | Holt2012 | 399 | 20,493 | 4,418,655 | 25 | 56.61 |
| ERR025726 | *S. sonnei* | Holt2012 | 363 | 23,309 | 4,545,055 | 25 | 60.99 |
| ERR025727 | *S. sonnei* | Holt2012 | 328 | 24,924 | 4,490,184 | 22 | 45.8 |
| ERR025729 | *S. sonnei* | Holt2012 | 382 | 19,438 | 4,320,587 | 25 | 62.95 |
| ERR025730 | *S. sonnei* | Holt2012 | 416 | 19,443 | 4,427,859 | 15 | 47.02 |
| ERR025731 | *S. sonnei* | Holt2012 | 369 | 22,065 | 4,548,598 | 23 | 64.62 |
| ERR025732 | *S. sonnei* | Holt2012 | 355 | 23,625 | 4,438,553 | 96 | 60.73 |
| ERR025734 | *S. sonnei* | Holt2012 | 347 | 22,661 | 4,459,800 | 33 | 48.3 |
| ERR025735 | *S. sonnei* | Holt2012 | 338 | 22,660 | 4,369,822 | 17 | 39.85 |
| ERR025737 | *S. sonnei* | Holt2012 | 331 | 24,020 | 4,581,007 | 30 | 54.62 |
| ERR025738 | *S. sonnei* | Holt2012 | 360 | 23,562 | 4,493,819 | 23 | 41.21 |
| ERR025741 | *S. sonnei* | Holt2012 | 379 | 22,308 | 4,432,034 | 25 | 45.77 |
| ERR025742 | *S. sonnei* | Holt2012 | 328 | 24,299 | 4,326,330 | 29 | 52.56 |
| ERR025743 | *S. sonnei* | Holt2012 | 306 | 24,458 | 4,342,922 | 31 | 49.55 |
| ERR025744 | *S. sonnei* | Holt2012 | 380 | 21,444 | 4,452,165 | 17 | 31.58 |
| ERR025746 | *S. sonnei* | Holt2012 | 312 | 25,071 | 4,365,185 | 57 | 72.62 |
| ERR025747 | *S. sonnei* | Holt2012 | 330 | 22,304 | 4,165,479 | 30 | 63.74 |
| ERR025749 | *S. sonnei* | Holt2012 | 325 | 22,110 | 4,117,720 | 56 | 69.31 |
| ERR025750 | *S. sonnei* | Holt2012 | 343 | 21,261 | 4,225,787 | 42 | 71.64 |
| ERR025751 | *S. sonnei* | Holt2012 | 338 | 25,174 | 4,450,336 | 22 | 60.11 |

| | | | | | | | |
|---|---|---|---|---|---|---|---|
| ERR025752 | *S. sonnei* | Holt2012 | 386 | 20,038 | 4,412,159 | 14 | 36.24 |
| ERR025753 | *S. sonnei* | Holt2012 | 313 | 23,919 | 4,334,279 | 57 | 81.68 |
| ERR025754 | *S. sonnei* | Holt2012 | 352 | 23,727 | 4,585,389 | 39 | 66.68 |
| ERR025755 | *S. sonnei* | Holt2012 | 342 | 23,562 | 4,525,468 | 48 | 72.23 |
| ERR025756 | *S. sonnei* | Holt2012 | 355 | 23,665 | 4,441,801 | 37 | 63.47 |
| ERR025758 | *S. sonnei* | Holt2012 | 337 | 23,188 | 4,379,849 | 49 | 64.11 |
| ERR025759 | *S. sonnei* | Holt2012 | 348 | 21,075 | 4,304,924 | 24 | 38.82 |
| ERR025761 | *S. sonnei* | Holt2012 | 359 | 22,065 | 4,320,523 | 57 | 81.14 |
| ERR025762 | *S. sonnei* | Holt2012 | 347 | 25,000 | 4,555,358 | 40 | 68.97 |
| ERR025763 | *S. sonnei* | Holt2012 | 432 | 17,882 | 4,428,758 | 14 | 57.24 |
| ERR025765 | *S. sonnei* | Holt2012 | 349 | 24,502 | 4,606,588 | 38 | 70.38 |
| ERR025767 | *S. sonnei* | Holt2012 | 367 | 22,203 | 4,526,100 | 50 | 61.35 |
| ERR025768 | *S. sonnei* | Holt2012 | 348 | 22,202 | 4,279,147 | 28 | 56.92 |
| ERR028671 | *S. sonnei* | Holt2012 | 328 | 24,067 | 4,341,066 | 21 | 88.2 |
| ERR028672 | *S. sonnei* | Holt2012 | 322 | 25,218 | 4,596,569 | 27 | 90.07 |
| ERR028673 | *S. sonnei* | Holt2012 | 320 | 27,582 | 4,612,765 | 204 | 86.84 |
| ERR028674 | *S. sonnei* | Holt2012 | 330 | 26,823 | 4,620,277 | 62 | 83.56 |
| ERR028675 | *S. sonnei* | Holt2012 | 313 | 27,371 | 4,535,219 | 27 | 84.25 |
| ERR028676 | *S. sonnei* | Holt2012 | 326 | 25,218 | 4,423,146 | 43 | 90.3 |
| ERR028677 | *S. sonnei* | Holt2012 | 328 | 23,269 | 4,290,403 | 26 | 85.52 |
| ERR028678 | *S. sonnei* | Holt2012 | 354 | 22,101 | 4,438,913 | 21 | 88.61 |
| ERR028679 | *S. sonnei* | Holt2012 | 346 | 23,356 | 4,394,443 | 39 | 91.25 |
| ERR028680 | *S. sonnei* | Holt2012 | 324 | 25,626 | 4,603,459 | 25 | 87.51 |
| ERR028681 | *S. sonnei* | Holt2012 | 336 | 22,466 | 4,315,558 | 19 | 85.5 |
| ERR028684 | *S. sonnei* | Holt2012 | 321 | 24,917 | 4,372,314 | 49 | 81.98 |
| ERR028685 | *S. sonnei* | Holt2012 | 360 | 23,468 | 4,590,075 | 78 | 86.79 |
| ERR028686 | *S. sonnei* | Holt2012 | 364 | 23,352 | 4,536,160 | 33 | 84.75 |
| ERR028687 | *S. sonnei* | Holt2012 | 361 | 23,966 | 4,598,307 | 53 | 87.53 |
| ERR028688 | *S. sonnei* | Holt2012 | 333 | 25,218 | 4,362,756 | 41 | 88.71 |
| ERR028689 | *S. sonnei* | Holt2012 | 350 | 23,353 | 4,469,701 | 70 | 84.86 |
| ERR028690 | *S. sonnei* | Holt2012 | 354 | 24,347 | 4,543,010 | 35 | 87.01 |
| ERR028691 | *S. sonnei* | Holt2012 | 359 | 23,385 | 4,437,348 | 28 | 91.77 |
| ERR028692 | *S. sonnei* | Holt2012 | 293 | 30,274 | 4,525,389 | 67 | 90.79 |
| ERR028693 | *S. sonnei* | Holt2012 | 349 | 23,967 | 4,495,243 | 43 | 85.98 |
| ERR028694 | *S. sonnei* | Holt2012 | 356 | 23,490 | 4,559,943 | 44 | 89.85 |
| ERR028695 | *S. sonnei* | Holt2012 | 356 | 24,347 | 4,557,641 | 60 | 88.29 |
| ERR028697 | *S. sonnei* | Holt2012 | 354 | 23,963 | 4,489,200 | 101 | 87 |
| ERR028699 | *S. sonnei* | Holt2012 | 365 | 23,115 | 4,530,909 | 140 | 87.59 |
| ERR028700 | *S. sonnei* | Holt2012 | 360 | 25,218 | 4,755,906 | 66 | 85.22 |
| ERR028702 | *S. sonnei* | Holt2012 | 361 | 23,963 | 4,616,919 | 168 | 94.2 |
| ERR028703 | *S. sonnei* | Holt2012 | 360 | 23,967 | 4,520,727 | 184 | 93.8 |
| ERR028704 | *S. sonnei* | Holt2012 | 372 | 23,592 | 4,642,557 | 99 | 91.1 |
| ERR028705 | *S. sonnei* | Holt2012 | 328 | 23,687 | 4,391,637 | 103 | 93.56 |
| ERR028706 | *S. sonnei* | Holt2012 | 343 | 26,824 | 4,558,607 | 55 | 96.37 |
| S13BD00834 | *S. sonnei* | NRC | 318 | 25,688 | 4,431,906 | 49 | 96.35 |
| S13BD00906 | *S. sonnei* | NRC | 322 | 26,034 | 4,563,563 | 86 | 95.6 |
| S13BD02106 | *S. sonnei* | NRC | 340 | 25,389 | 4,509,103 | 38 | 97.28 |
| S13BD02107 | *S. sonnei* | NRC | 350 | 25,389 | 4,632,951 | 35 | 96.52 |
| S13BD02670 | *S. sonnei* | NRC | 349 | 24,765 | 4,557,148 | 37 | 96.69 |
| S13BD02671 | *S. sonnei* | NRC | 346 | 25,690 | 4,570,104 | 52 | 97.68 |

| | | | | | | | | |
|---|---|---|---|---|---|---|---|---|
| S13BD03196 | *S. sonnei* | NRC | 315 | 25,362 | 4,420,831 | 48 | 96.05 |
| S13BD03288 | *S. sonnei* | NRC | 349 | 25,752 | 4,667,690 | 51 | 96.82 |
| S13BD03539 | *S. sonnei* | NRC | 328 | 24,765 | 4,430,827 | 35 | 94.77 |
| S13BD04360 | *S. sonnei* | NRC | 327 | 25,688 | 4,494,704 | 58 | 96.35 |
| S14BD00913 | *S. sonnei* | NRC | 337 | 25,093 | 4,477,811 | 67 | 96.88 |
| S14BD03054 | *S. sonnei* | NRC | 326 | 25,905 | 4,634,628 | 46 | 95.12 |
| S14BD03397 | *S. sonnei* | NRC | 351 | 24,881 | 4,593,894 | 52 | 96.63 |
| S14BD03573 | *S. sonnei* | NRC | 327 | 25,362 | 4,429,614 | 35 | 95.91 |
| S14BD04882 | *S. sonnei* | NRC | 342 | 25,689 | 4,607,882 | 34 | 96.69 |
| S14BD04971 | *S. sonnei* | NRC | 313 | 25,688 | 4,383,773 | 35 | 96.26 |
| S14BD05043 | *S. sonnei* | NRC | 336 | 25,721 | 4,524,343 | 24 | 97.49 |
| S15BD00569 | *S. sonnei* | NRC | 345 | 25,362 | 4,515,235 | 34 | 96.3 |
| S15BD00797 | *S. sonnei* | NRC | 377 | 20,658 | 4,490,735 | 19 | 96.09 |
| S15BD00889 | *S. sonnei* | NRC | 362 | 25,093 | 4,553,436 | 35 | 97.49 |
| S15BD00932 | *S. sonnei* | NRC | 364 | 25,362 | 4,647,386 | 21 | 96.66 |
| S15BD01283 | *S. sonnei* | NRC | 349 | 25,752 | 4,659,191 | 55 | 96.84 |
| S15BD02324 | *S. sonnei* | NRC | 319 | 25,688 | 4,524,748 | 42 | 97.14 |
| S15BD02325 | *S. sonnei* | NRC | 349 | 25,905 | 4,658,115 | 43 | 96.99 |
| S15BD02326 | *S. sonnei* | NRC | 320 | 25,752 | 4,510,656 | 44 | 96.01 |
| S15BD02407 | *S. sonnei* | NRC | 350 | 25,752 | 4,670,416 | 78 | 95.9 |
| S15BD02515 | *S. sonnei* | NRC | 359 | 25,689 | 4,709,465 | 62 | 97.57 |
| S15BD02737 | *S. sonnei* | NRC | 322 | 25,686 | 4,429,330 | 33 | 96.75 |
| S15BD02979 | *S. sonnei* | NRC | 331 | 25,905 | 4,571,675 | 25 | 96.88 |
| S15BD02995 | *S. sonnei* | NRC | 355 | 26,035 | 4,654,654 | 64 | 96.85 |
| S15BD03192 | *S. sonnei* | NRC | 332 | 26,034 | 4,553,643 | 23 | 97.58 |
| S15BD03801 | *S. sonnei* | NRC | 325 | 25,905 | 4,527,284 | 23 | 97.06 |
| S15BD03806 | *S. sonnei* | NRC | 320 | 25,389 | 4,530,957 | 56 | 96.22 |
| S15BD03807 | *S. sonnei* | NRC | 346 | 25,752 | 4,738,278 | 34 | 97.62 |
| S15BD03808 | *S. sonnei* | NRC | 348 | 25,389 | 4,659,376 | 39 | 97.05 |
| S15BD03850 | *S. sonnei* | NRC | 346 | 24,288 | 4,538,855 | 35 | 97.2 |
| S15BD04030 | *S. sonnei* | NRC | 320 | 25,389 | 4,424,922 | 50 | 96.07 |
| S15BD04031 | *S. sonnei* | NRC | 351 | 25,093 | 4,540,401 | 35 | 95.87 |
| S15BD04165 | *S. sonnei* | NRC | 351 | 25,718 | 4,677,053 | 37 | 96.24 |
| S15BD04166 | *S. sonnei* | NRC | 326 | 25,685 | 4,457,063 | 27 | 96.87 |
| S15BD04402 | *S. sonnei* | NRC | 312 | 25,688 | 4,400,769 | 64 | 95.57 |
| S15BD04792 | *S. sonnei* | NRC | 365 | 25,362 | 4,588,485 | 18 | 96.03 |
| S15BD04848 | *S. sonnei* | NRC | 333 | 25,905 | 4,690,953 | 25 | 97.55 |
| S15BD05940 | *S. sonnei* | NRC | 328 | 25,752 | 4,446,717 | 24 | 96.35 |
| S15BD06471 | *S. sonnei* | NRC | 348 | 25,689 | 4,673,660 | 28 | 95.96 |
| S15BD07017 | *S. sonnei* | NRC | 342 | 25,362 | 4,571,296 | 46 | 96.82 |
| S15BD07161 | *S. sonnei* | NRC | 320 | 26,034 | 4,600,815 | 53 | 95.63 |
| S15BD07522 | *S. sonnei* | NRC | 317 | 25,093 | 4,353,346 | 58 | 96.11 |
| S15BD09124 | *S. sonnei* | NRC | 337 | 25,090 | 4,428,339 | 20 | 95.92 |
| S15BD09162 | *S. sonnei* | NRC | 322 | 25,752 | 4,507,217 | 53 | 96.36 |
| S15BD09356 | *S. sonnei* | NRC | 394 | 19,980 | 4,400,601 | 14 | 95.37 |
| S15BD09628 | *S. sonnei* | NRC | 358 | 25,093 | 4,585,055 | 48 | 96.4 |
| S15BD09656 | *S. sonnei* | NRC | 346 | 25,362 | 4,499,448 | 57 | 96.18 |
| S15BD10104 | *S. sonnei* | NRC | 349 | 23,736 | 4,530,451 | 34 | 96.5 |
| S15BD10108 | *S. sonnei* | NRC | 358 | 24,288 | 4,561,895 | 40 | 96.71 |
| S15BD10196 | *S. sonnei* | NRC | 316 | 25,093 | 4,401,307 | 77 | 95.91 |

| | | | | | | | | |
|---|---|---|---|---|---|---|---|---|
| S15BD10296 | *S. sonnei* | NRC | 357 | 25,362 | 4,571,975 | 31 | 97.02 |
| S16BD00285 | *S. sonnei* | NRC | 376 | 20,967 | 4,485,133 | 17 | 97.33 |
| S16BD00582 | *S. sonnei* | NRC | 439 | 18,780 | 4,420,290 | 13 | 95.56 |
| S16BD00590 | *S. sonnei* | NRC | 342 | 23,743 | 4,353,982 | 24 | 97.21 |
| S16BD00864 | *S. sonnei* | NRC | 612 | 5,041 | 2,180,785 | 10 | 62.76 |
| S16BD01339 | *S. sonnei* | NRC | 392 | 22,253 | 4,506,415 | 21 | 95.64 |
| S16BD01740 | *S. sonnei* | NRC | 499 | 16,925 | 4,566,783 | 12 | 96.14 |
| S16BD01790 | *S. sonnei* | NRC | 432 | 20,404 | 4,426,329 | 13 | 97.23 |
| S16BD02550 | *S. sonnei* | NRC | 233 | 1,968 | 457,748 | 9 | 25.24 |
| S16BD02574 | *S. sonnei* | NRC | 456 | 18,016 | 4,412,759 | 12 | 95.36 |
| S16BD03340 | *S. sonnei* | NRC | 384 | 20,381 | 4,468,742 | 16 | 96.58 |
| S16BD04069 | *S. sonnei* | NRC | 402 | 20,699 | 4,442,828 | 18 | 96.55 |
| S17BD01977 | *S. sonnei* | NRC | 604 | 13,291 | 3,926,773 | 11 | 79.86 |
| S17BD02088 | *S. sonnei* | NRC | 324 | 27,760 | 4,672,550 | 26 | 95.54 |
| S17BD02164 | *S. sonnei* | NRC | 347 | 27,365 | 4,757,295 | 52 | 94.01 |
| S17BD02526 | *S. sonnei* | NRC | 334 | 27,760 | 4,744,044 | 70 | 96.27 |
| S17BD02897 | *S. sonnei* | NRC | 348 | 25,362 | 4,691,437 | 34 | 96.71 |
| S17BD03513 | *S. sonnei* | NRC | 305 | 25,905 | 4,420,806 | 29 | 97.19 |
| S17BD04438 | *S. sonnei* | NRC | 307 | 29,174 | 4,713,302 | 30 | 97.51 |
| S17BD06426 | *S. sonnei* | NRC | 351 | 25,389 | 4,588,596 | 27 | 93.6 |
| S17BD06464 | *S. sonnei* | NRC | 350 | 25,093 | 4,558,442 | 39 | 89.22 |
| S17BD06759 | *S. sonnei* | NRC | 314 | 25,905 | 4,507,542 | 37 | 96.96 |
| S17BD06760 | *S. sonnei* | NRC | 341 | 25,752 | 4,702,512 | 28 | 85.29 |
| S17BD06799 | *S. sonnei* | NRC | 337 | 25,389 | 4,579,048 | 29 | 83.69 |
| S17BD06801 | *S. sonnei* | NRC | 333 | 25,905 | 4,677,080 | 27 | 93.6 |
| S17BD07774 | *S. sonnei* | NRC | 340 | 25,362 | 4,625,615 | 22 | 82.2 |
| S17BD07961 | *S. sonnei* | NRC | 322 | 25,362 | 4,416,549 | 31 | 88.39 |
| S18BD00011 | *S. sonnei* | NRC | 323 | 25,663 | 4,592,423 | 35 | 96.57 |
| S18BD00105 | *S. sonnei* | NRC | 354 | 25,752 | 4,720,189 | 31 | 81.96 |
| S18BD00654 | *S. sonnei* | NRC | 327 | 26,034 | 4,588,322 | 24 | 93.18 |
| S18BD01122 | *S. sonnei* | NRC | 298 | 27,654 | 4,416,112 | 28 | 97.11 |
| S18BD01231 | *S. sonnei* | NRC | 353 | 25,752 | 4,823,199 | 34 | 92.35 |
| S18BD01685 | *S. sonnei* | NRC | 328 | 25,906 | 4,664,797 | 27 | 84.08 |
| S18BD01986 | *S. sonnei* | NRC | 377 | 25,760 | 4,842,947 | 31 | 92.66 |
| S18BD02200 | *S. sonnei* | NRC | 327 | 24,536 | 4,461,350 | 35 | 80.78 |
| ERR586833 | *S. sonnei* | The2018 | 333 | 23,592 | 4,401,895 | 81 | 96.02 |
| ERR586837 | *S. sonnei* | The2018 | 330 | 25,218 | 4,400,435 | 71 | 95.76 |
| ERR586838 | *S. sonnei* | The2018 | 335 | 24,068 | 4,406,668 | 70 | 95.88 |
| ERR591154 | *S. sonnei* | The2018 | 366 | 23,592 | 4,597,220 | 89 | 95.96 |
| ERR591156 | *S. sonnei* | The2018 | 354 | 23,963 | 4,568,602 | 80 | 95.92 |
| ERR591166 | *S. sonnei* | The2018 | 325 | 24,068 | 4,350,694 | 89 | 95.4 |
| ERR591176 | *S. sonnei* | The2018 | 328 | 23,353 | 4,346,920 | 98 | 95.09 |
| ERR591179 | *S. sonnei* | The2018 | 351 | 23,592 | 4,466,132 | 81 | 95.14 |
| ERR591185 | *S. sonnei* | The2018 | 322 | 24,671 | 4,344,086 | 80 | 93.29 |
| ERR591188 | *S. sonnei* | The2018 | 324 | 23,592 | 4,341,436 | 77 | 95.74 |
| ERR591211 | *S. sonnei* | The2018 | 357 | 23,966 | 4,491,047 | 87 | 94.18 |
| ERR591218 | *S. sonnei* | The2018 | 329 | 24,348 | 4,382,963 | 81 | 95.69 |
| ERR591405 | *S. sonnei* | The2018 | 359 | 23,967 | 4,486,573 | 75 | 94.71 |
| ERR592006 | *S. sonnei* | The2018 | 359 | 24,348 | 4,583,179 | 107 | 96.22 |
| ERR592007 | *S. sonnei* | The2018 | 362 | 23,889 | 4,500,081 | 104 | 94.34 |

| | | | | | | | | |
|---|---|---|---|---|---|---|---|---|
| ERR592013 | *S. sonnei* | The2018 | 353 | 24,949 | 4,479,121 | 73 | 94.06 |
| ERR592030 | *S. sonnei* | The2018 | 361 | 23,890 | 4,492,399 | 85 | 95.73 |
| ERR592189 | *S. sonnei* | The2018 | 360 | 23,353 | 4,462,860 | 98 | 94.97 |
| ERR592269 | *S. sonnei* | The2018 | 368 | 24,068 | 4,605,402 | 92 | 94.33 |
| ERR592270 | *S. sonnei* | The2018 | 363 | 23,963 | 4,485,888 | 97 | 95.11 |
| ERR592449 | *S. sonnei* | The2018 | 361 | 23,592 | 4,488,678 | 84 | 94.98 |
| ERR708271 | *S. sonnei* | The2018 | 332 | 23,353 | 4,352,229 | 106 | 95.91 |
| ERR708272 | *S. sonnei* | The2018 | 327 | 23,965 | 4,353,979 | 117 | 94.67 |
| ERR708273 | *S. sonnei* | The2018 | 319 | 25,218 | 4,375,249 | 123 | 95.9 |
| ERR708274 | *S. sonnei* | The2018 | 366 | 23,890 | 4,583,560 | 131 | 95.61 |
| ERR708275 | *S. sonnei* | The2018 | 328 | 23,353 | 4,341,189 | 117 | 95.3 |
| ERR708276 | *S. sonnei* | The2018 | 362 | 23,890 | 4,487,861 | 102 | 95.65 |
| ERR708277 | *S. sonnei* | The2018 | 338 | 23,592 | 4,390,289 | 112 | 94.78 |
| ERR708278 | *S. sonnei* | The2018 | 325 | 25,538 | 4,449,455 | 90 | 95.23 |
| ERR708279 | *S. sonnei* | The2018 | 327 | 23,353 | 4,402,096 | 103 | 95.2 |
| ERR708280 | *S. sonnei* | The2018 | 330 | 23,592 | 4,354,974 | 115 | 96.71 |
| ERR708281 | *S. sonnei* | The2018 | 359 | 23,890 | 4,480,253 | 168 | 94.41 |
| ERR708282 | *S. sonnei* | The2018 | 321 | 25,218 | 4,397,183 | 129 | 94.18 |
| ERR708283 | *S. sonnei* | The2018 | 324 | 24,724 | 4,408,284 | 103 | 94.17 |
| ERR708284 | *S. sonnei* | The2018 | 329 | 24,587 | 4,403,418 | 120 | 94.88 |
| ERR708285 | *S. sonnei* | The2018 | 333 | 24,949 | 4,353,935 | 103 | 95.28 |
| ERR708286 | *S. sonnei* | The2018 | 328 | 24,949 | 4,449,008 | 105 | 94.85 |
| ERR708287 | *S. sonnei* | The2018 | 329 | 25,245 | 4,540,465 | 105 | 95.58 |
| ERR708288 | *S. sonnei* | The2018 | 364 | 24,348 | 4,670,973 | 106 | 94.17 |
| ERR708289 | *S. sonnei* | The2018 | 365 | 23,890 | 4,655,093 | 121 | 94.15 |
| ERR708290 | *S. sonnei* | The2018 | 330 | 25,245 | 4,561,189 | 118 | 94.23 |
| SRR1605255 | *S. sonnei* | The2018 | 326 | 25,752 | 4,509,584 | 65 | 96.6 |
| SRR2081185 | *S. sonnei* | The2018 | 336 | 26,828 | 4,676,824 | 74 | 97.06 |
| SRR2081189 | *S. sonnei* | The2018 | 328 | 25,389 | 4,411,790 | 97 | 96.53 |
| SRR2087717 | *S. sonnei* | The2018 | 317 | 25,905 | 4,511,224 | 73 | 97.05 |
| SRR2097498 | *S. sonnei* | The2018 | 324 | 25,390 | 4,512,643 | 47 | 96.69 |
| SRR3473843 | *S. sonnei* | The2018 | 348 | 23,313 | 4,364,414 | 88 | 95.26 |
| SRR3473844 | *S. sonnei* | The2018 | 345 | 23,313 | 4,354,424 | 91 | 95.42 |
| SRR3473847 | *S. sonnei* | The2018 | 345 | 23,313 | 4,352,501 | 93 | 93.64 |
| SRR3473848 | *S. sonnei* | The2018 | 345 | 22,393 | 4,422,779 | 95 | 95.56 |
| SRR3473849 | *S. sonnei* | The2018 | 352 | 21,802 | 4,361,059 | 94 | 95.51 |
| SRR3473850 | *S. sonnei* | The2018 | 322 | 24,993 | 4,364,872 | 60 | 95.44 |
| SRR3473853 | *S. sonnei* | The2018 | 325 | 23,636 | 4,359,218 | 32 | 95.08 |
| SRR3473854 | *S. sonnei* | The2018 | 321 | 25,652 | 4,473,211 | 50 | 92.98 |
| SRR3473855 | *S. sonnei* | The2018 | 344 | 22,437 | 4,358,847 | 100 | 94.57 |
| SRR3473856 | *S. sonnei* | The2018 | 356 | 23,880 | 4,353,489 | 90 | 94.21 |
| SRR3473857 | *S. sonnei* | The2018 | 344 | 23,637 | 4,394,262 | 98 | 95.79 |
| SRR3473858 | *S. sonnei* | The2018 | 327 | 25,652 | 4,341,262 | 100 | 96.28 |
| SRR3473859 | *S. sonnei* | The2018 | 348 | 23,398 | 4,361,237 | 95 | 95.38 |
| SRR3473860 | *S. sonnei* | The2018 | 322 | 23,934 | 4,210,522 | 102 | 95.79 |
| SRR3473861 | *S. sonnei* | The2018 | 357 | 25,590 | 4,534,216 | 78 | 96.4 |
| SRR3473862 | *S. sonnei* | The2018 | 350 | 23,636 | 4,394,943 | 98 | 95.62 |
| SRR3474162 | *S. sonnei* | The2018 | 338 | 23,756 | 4,420,498 | 20 | 96.9 |
| SRR3474163 | *S. sonnei* | The2018 | 9 | 9,555 | 77,416 | 105 | 19.46 |
| SRR3474164 | *S. sonnei* | The2018 | 333 | 25,690 | 4,628,590 | 21 | 96.37 |

| | | | | | | | |
|---|---|---|---|---|---|---|---|
| SRR3474165 | *S. sonnei* | The2018 | 366 | 25,752 | 4,714,206 | 41 | 96.5 |
| SRR3474166 | *S. sonnei* | The2018 | 412 | 23,736 | 4,793,675 | 20 | 96.08 |
| SRR3474167 | *S. sonnei* | The2018 | 345 | 24,180 | 4,418,439 | 26 | 97.17 |
| SRR3474168 | *S. sonnei* | The2018 | 375 | 23,415 | 4,444,819 | 18 | 96.6 |
| SRR3474169 | *S. sonnei* | The2018 | 392 | 22,395 | 4,586,228 | 18 | 97.55 |
| SRR3474170 | *S. sonnei* | The2018 | 58 | 5,594 | 241,367 | 30 | 24.83 |
| SRR3474171 | *S. sonnei* | The2018 | 21 | 5,241 | 68,261 | 225 | 24.28 |
| SRR3474172 | *S. sonnei* | The2018 | 36 | 5,241 | 130,600 | 58 | 16.46 |

**Supplementary Table S4: Percentage of cgMLST alleles identified.**

| Sample | Species | Collection | % cgMLST alleles identified |
|---|---|---|---|
| ERR316291 | *S. sonnei* | Baker2017 | 99.44 |
| ERR316299 | *S. sonnei* | Baker2017 | 98.89 |
| ERR316306 | *S. sonnei* | Baker2017 | 99.52 |
| ERR586833 | *S. sonnei* | The2016 | 99.52 |
| ERR586837 | *S. sonnei* | The2016 | 99.44 |
| ERR586838 | *S. sonnei* | The2016 | 99.44 |
| ERR591154 | *S. sonnei* | The2016 | 99.48 |
| ERR591156 | *S. sonnei* | The2016 | 99.56 |
| ERR591166 | *S. sonnei* | The2016 | 99.52 |
| ERR591176 | *S. sonnei* | The2016 | 99.44 |
| ERR591179 | *S. sonnei* | The2016 | 99.40 |
| ERR591185 | *S. sonnei* | The2016 | 99.44 |
| ERR591188 | *S. sonnei* | The2016 | 99.24 |
| ERR591211 | *S. sonnei* | The2016 | 99.44 |
| ERR591218 | *S. sonnei* | The2016 | 99.48 |
| ERR591405 | *S. sonnei* | The2016 | 99.40 |
| ERR592006 | *S. sonnei* | The2016 | 99.56 |
| ERR592007 | *S. sonnei* | The2016 | 98.93 |
| ERR592013 | *S. sonnei* | The2016 | 99.40 |
| ERR592030 | *S. sonnei* | The2016 | 99.28 |
| ERR592189 | *S. sonnei* | The2016 | 99.32 |
| ERR592269 | *S. sonnei* | The2016 | 99.28 |
| ERR592270 | *S. sonnei* | The2016 | 99.32 |
| ERR592449 | *S. sonnei* | The2016 | 99.32 |
| ERR708271 | *S. sonnei* | The2016 | 99.16 |
| ERR708272 | *S. sonnei* | The2016 | 99.32 |
| ERR708273 | *S. sonnei* | The2016 | 99.68 |
| ERR708274 | *S. sonnei* | The2016 | 99.52 |
| ERR708275 | *S. sonnei* | The2016 | 99.40 |
| ERR708276 | *S. sonnei* | The2016 | 99.40 |
| ERR708277 | *S. sonnei* | The2016 | 99.48 |
| ERR708278 | *S. sonnei* | The2016 | 99.60 |
| ERR708279 | *S. sonnei* | The2016 | 99.40 |
| ERR708280 | *S. sonnei* | The2016 | 99.56 |
| ERR708281 | *S. sonnei* | The2016 | 99.44 |
| ERR708282 | *S. sonnei* | The2016 | 99.52 |
| ERR708283 | *S. sonnei* | The2016 | 99.28 |
| ERR708284 | *S. sonnei* | The2016 | 96.26 |
| ERR708285 | *S. sonnei* | The2016 | 99.32 |
| ERR708286 | *S. sonnei* | The2016 | 99.16 |
| ERR708287 | *S. sonnei* | The2016 | 98.97 |
| ERR708288 | *S. sonnei* | The2016 | 99.08 |
| ERR708289 | *S. sonnei* | The2016 | 99.44 |
| ERR708290 | *S. sonnei* | The2016 | 99.08 |
| SRR1605255 | *S. sonnei* | The2016 | 99.40 |
| SRR2081185 | *S. sonnei* | The2016 | 99.44 |
| SRR2081189 | *S. sonnei* | The2016 | 99.28 |

| | | | |
|---|---|---|---|
| SRR2087717 | *S. sonnei* | The2016 | 99.16 |
| SRR2097498 | *S. sonnei* | The2016 | 99.52 |
| SRR3473843 | *S. sonnei* | The2016 | 99.44 |
| SRR3473844 | *S. sonnei* | The2016 | 99.40 |
| SRR3473847 | *S. sonnei* | The2016 | 99.36 |
| SRR3473848 | *S. sonnei* | The2016 | 99.40 |
| SRR3473849 | *S. sonnei* | The2016 | 99.12 |
| SRR3473850 | *S. sonnei* | The2016 | 99.32 |
| SRR3473853 | *S. sonnei* | The2016 | 99.52 |
| SRR3473854 | *S. sonnei* | The2016 | 99.44 |
| SRR3473855 | *S. sonnei* | The2016 | 99.48 |
| SRR3473856 | *S. sonnei* | The2016 | 99.40 |
| SRR3473857 | *S. sonnei* | The2016 | 99.32 |
| SRR3473858 | *S. sonnei* | The2016 | 99.20 |
| SRR3473859 | *S. sonnei* | The2016 | 99.28 |
| SRR3473860 | *S. sonnei* | The2016 | 99.20 |
| SRR3473861 | *S. sonnei* | The2016 | 99.32 |
| SRR3473862 | *S. sonnei* | The2016 | 98.61 |
| SRR3474162 | *S. sonnei* | The2016 | 99.28 |
| SRR3474163 | *S. sonnei* | The2016 | 99.48 |
| SRR3474164 | *S. sonnei* | The2016 | 99.12 |
| SRR3474165 | *S. sonnei* | The2016 | 99.20 |
| SRR3474166 | *S. sonnei* | The2016 | 99.44 |
| SRR3474167 | *S. sonnei* | The2016 | 99.20 |
| SRR3474168 | *S. sonnei* | The2016 | 97.89 |
| SRR3474169 | *S. sonnei* | The2016 | 99.36 |
| SRR3474170 | *S. sonnei* | The2016 | 99.48 |
| SRR3474171 | *S. sonnei* | The2016 | 99.12 |
| SRR3474172 | *S. sonnei* | The2016 | 99.20 |
| ERR042796 | *S. flexneri* | Connor2015 | 98.81 |
| ERR042797 | *S. flexneri* | Connor2015 | 99.08 |
| ERR042799 | *S. flexneri* | Connor2015 | 97.41 |
| ERR042803 | *S. flexneri* | Connor2015 | 98.89 |
| ERR042806 | *S. flexneri* | Connor2015 | 34.94 |
| ERR042810 | *S. flexneri* | Connor2015 | 99.28 |
| ERR042811 | *S. flexneri* | Connor2015 | 97.45 |
| ERR042814 | *S. flexneri* | Connor2015 | 99.20 |
| ERR042816 | *S. flexneri* | Connor2015 | 99.20 |
| ERR042819 | *S. flexneri* | Connor2015 | 99.24 |
| ERR042821 | *S. flexneri* | Connor2015 | 94.67 |
| ERR042824 | *S. flexneri* | Connor2015 | 99.48 |
| ERR042825 | *S. flexneri* | Connor2015 | 99.24 |
| ERR042827 | *S. flexneri* | Connor2015 | 99.24 |
| ERR042831 | *S. flexneri* | Connor2015 | 98.89 |
| ERR042832 | *S. flexneri* | Connor2015 | 99.40 |
| ERR042833 | *S. flexneri* | Connor2015 | 98.21 |
| ERR042835 | *S. flexneri* | Connor2015 | 99.20 |
| ERR042837 | *S. flexneri* | Connor2015 | 98.25 |
| ERR042838 | *S. flexneri* | Connor2015 | 99.12 |
| ERR042839 | *S. flexneri* | Connor2015 | 98.97 |

| | | | |
|---|---|---|---|
| ERR042840 | *S. flexneri* | Connor2015 | 99.20 |
| ERR042841 | *S. flexneri* | Connor2015 | 99.20 |
| ERR042842 | *S. flexneri* | Connor2015 | 97.89 |
| ERR042843 | *S. flexneri* | Connor2015 | 97.69 |
| ERR042845 | *S. flexneri* | Connor2015 | 97.41 |
| ERR042849 | *S. flexneri* | Connor2015 | 98.49 |
| ERR042851 | *S. flexneri* | Connor2015 | 97.61 |
| ERR042852 | *S. flexneri* | Connor2015 | 99.04 |
| ERR042853 | *S. flexneri* | Connor2015 | 99.20 |
| ERR042855 | *S. flexneri* | Connor2015 | 99.52 |
| ERR042858 | *S. flexneri* | Connor2015 | 99.40 |
| ERR042860 | *S. flexneri* | Connor2015 | 99.20 |
| ERR042861 | *S. flexneri* | Connor2015 | 99.32 |
| ERR042863 | *S. flexneri* | Connor2015 | 99.52 |
| ERR047236 | *S. flexneri* | Connor2015 | 99.36 |
| ERR047239 | *S. flexneri* | Connor2015 | 99.12 |
| ERR047294 | *S. flexneri* | Connor2015 | 98.49 |
| ERR047297 | *S. flexneri* | Connor2015 | 99.60 |
| ERR047306 | *S. flexneri* | Connor2015 | 98.29 |
| ERR047307 | *S. flexneri* | Connor2015 | 99.20 |
| ERR047372 | *S. flexneri* | Connor2015 | 99.24 |
| ERR047396 | *S. flexneri* | Connor2015 | 99.32 |
| ERR047406 | *S. flexneri* | Connor2015 | 98.65 |
| ERR048234 | *S. flexneri* | Connor2015 | 98.29 |
| ERR048246 | *S. flexneri* | Connor2015 | 98.73 |
| ERR048259 | *S. flexneri* | Connor2015 | 98.01 |
| ERR048261 | *S. flexneri* | Connor2015 | 99.12 |
| ERR048265 | *S. flexneri* | Connor2015 | 99.36 |
| ERR048285 | *S. flexneri* | Connor2015 | 99.08 |
| ERR048286 | *S. flexneri* | Connor2015 | 99.20 |
| ERR048287 | *S. flexneri* | Connor2015 | 98.81 |
| ERR048288 | *S. flexneri* | Connor2015 | 99.12 |
| ERR048290 | *S. flexneri* | Connor2015 | 98.81 |
| ERR048295 | *S. flexneri* | Connor2015 | 99.12 |
| ERR048296 | *S. flexneri* | Connor2015 | 97.17 |
| ERR048300 | *S. flexneri* | Connor2015 | 97.89 |
| ERR048302 | *S. flexneri* | Connor2015 | 98.81 |
| ERR048304 | *S. flexneri* | Connor2015 | 99.12 |
| ERR048306 | *S. flexneri* | Connor2015 | 99.12 |
| ERR048311 | *S. flexneri* | Connor2015 | 98.29 |
| ERR048312 | *S. flexneri* | Connor2015 | 98.25 |
| ERR048313 | *S. flexneri* | Connor2015 | 99.04 |
| ERR048315 | *S. flexneri* | Connor2015 | 98.85 |
| ERR048316 | *S. flexneri* | Connor2015 | 98.41 |
| ERR048317 | *S. flexneri* | Connor2015 | 97.97 |
| ERR048319 | *S. flexneri* | Connor2015 | 98.65 |
| ERR048320 | *S. flexneri* | Connor2015 | 98.77 |
| ERR048322 | *S. flexneri* | Connor2015 | 99.16 |
| ERR048329 | *S. flexneri* | Connor2015 | 98.77 |
| ERR048331 | *S. flexneri* | Connor2015 | 41.62 |

| | | | |
|---|---|---|---|
| ERR048332 | *S. flexneri* | Connor2015 | 16.75 |
| ERR048333 | *S. flexneri* | Connor2015 | 99.16 |
| ERR048334 | *S. flexneri* | Connor2015 | 98.73 |
| ERR048339 | *S. flexneri* | Connor2015 | 98.45 |
| ERR049152 | *S. flexneri* | Connor2015 | 99.60 |
| ERR126958 | *S. flexneri* | Connor2015 | 98.69 |
| ERR127015 | *S. flexneri* | Connor2015 | 98.49 |
| ERR127017 | *S. flexneri* | Connor2015 | 97.53 |
| ERR127019 | *S. flexneri* | Connor2015 | 98.21 |
| ERR127032 | *S. flexneri* | Connor2015 | 99.24 |
| ERR127034 | *S. flexneri* | Connor2015 | 98.69 |
| ERR127035 | *S. flexneri* | Connor2015 | 98.45 |
| ERR127036 | *S. flexneri* | Connor2015 | 98.49 |
| ERR127037 | *S. flexneri* | Connor2015 | 98.01 |
| ERR127038 | *S. flexneri* | Connor2015 | 98.89 |
| ERR127039 | *S. flexneri* | Connor2015 | 98.65 |
| ERR127040 | *S. flexneri* | Connor2015 | 97.85 |
| ERR127041 | *S. flexneri* | Connor2015 | 98.45 |
| ERR127042 | *S. flexneri* | Connor2015 | 98.61 |
| ERR127043 | *S. flexneri* | Connor2015 | 97.49 |
| ERR127044 | *S. flexneri* | Connor2015 | 97.93 |
| ERR127046 | *S. flexneri* | Connor2015 | 98.97 |
| ERR127047 | *S. flexneri* | Connor2015 | 99.28 |
| ERR127048 | *S. flexneri* | Connor2015 | 88.14 |
| ERR200344 | *S. flexneri* | Connor2015 | 98.77 |
| ERR200360 | *S. flexneri* | Connor2015 | 96.94 |
| ERR200365 | *S. flexneri* | Connor2015 | 99.08 |
| ERR200370 | *S. flexneri* | Connor2015 | 97.49 |
| ERR200378 | *S. flexneri* | Connor2015 | 99.36 |
| ERR200379 | *S. flexneri* | Connor2015 | 93.35 |
| ERR200390 | *S. flexneri* | Connor2015 | 94.19 |
| ERR200392 | *S. flexneri* | Connor2015 | 97.53 |
| ERR200393 | *S. flexneri* | Connor2015 | 99.12 |
| ERR200402 | *S. flexneri* | Connor2015 | 87.94 |
| ERR200403 | *S. flexneri* | Connor2015 | 82.01 |
| ERR200405 | *S. flexneri* | Connor2015 | 87.98 |
| ERR200413 | *S. flexneri* | Connor2015 | 99.12 |
| ERR200414 | *S. flexneri* | Connor2015 | 97.73 |
| ERR200415 | *S. flexneri* | Connor2015 | 97.17 |
| ERR217012 | *S. flexneri* | Connor2015 | 97.45 |
| ERR217013 | *S. flexneri* | Connor2015 | 82.89 |
| ERR217015 | *S. flexneri* | Connor2015 | 85.87 |
| ERR217016 | *S. flexneri* | Connor2015 | 87.23 |
| ERR217022 | *S. flexneri* | Connor2015 | 78.07 |
| ERR217023 | *S. flexneri* | Connor2015 | 74.73 |
| ERR217024 | *S. flexneri* | Connor2015 | 98.41 |
| ERR217026 | *S. flexneri* | Connor2015 | 99.16 |
| ERR217028 | *S. flexneri* | Connor2015 | 98.53 |
| ERR217030 | *S. flexneri* | Connor2015 | 99.04 |
| ERR217031 | *S. flexneri* | Connor2015 | 98.53 |

| | | | |
|---|---|---|---|
| ERR217032 | *S. flexneri* | Connor2015 | 98.29 |
| ERR217033 | *S. flexneri* | Connor2015 | 99.32 |
| ERR217047 | *S. flexneri* | Connor2015 | 98.53 |
| ERR217080 | *S. flexneri* | Connor2015 | 99.08 |
| ERR217081 | *S. flexneri* | Connor2015 | 99.28 |
| ERR217084 | *S. flexneri* | Connor2015 | 99.40 |
| ERR559526 | *S. flexneri* | Connor2015 | 99.36 |
| ERR832453 | *S. flexneri* | Connor2015 | 99.32 |
| ERR832456 | *S. flexneri* | Connor2015 | 99.40 |
| ERR832457 | *S. flexneri* | Connor2015 | 99.32 |
| ERR832459 | *S. flexneri* | Connor2015 | 98.53 |
| ERR832460 | *S. flexneri* | Connor2015 | 98.73 |
| ERR832461 | *S. flexneri* | Connor2015 | 99.40 |
| ERR832462 | *S. flexneri* | Connor2015 | 99.60 |
| ERR832464 | *S. flexneri* | Connor2015 | 99.36 |
| ERR832465 | *S. flexneri* | Connor2015 | 98.89 |
| ERR832467 | *S. flexneri* | Connor2015 | 99.56 |
| ERR832468 | *S. flexneri* | Connor2015 | 98.17 |
| ERR832470 | *S. flexneri* | Connor2015 | 98.73 |
| ERR832471 | *S. flexneri* | Connor2015 | 99.64 |
| ERR832473 | *S. flexneri* | Connor2015 | 99.64 |
| ERR832474 | *S. flexneri* | Connor2015 | 98.45 |
| ERR832477 | *S. flexneri* | Connor2015 | 99.32 |
| ERR832480 | *S. flexneri* | Connor2015 | 99.04 |
| ERR832481 | *S. flexneri* | Connor2015 | 98.93 |
| ERR832483 | *S. flexneri* | Connor2015 | 98.49 |
| ERR832485 | *S. flexneri* | Connor2015 | 99.36 |
| ERR832486 | *S. flexneri* | Connor2015 | 98.49 |
| ERR832487 | *S. flexneri* | Connor2015 | 98.45 |
| ERR832489 | *S. flexneri* | Connor2015 | 99.12 |
| ERR832490 | *S. flexneri* | Connor2015 | 99.20 |
| ERR832491 | *S. flexneri* | Connor2015 | 99.04 |
| ERR832492 | *S. flexneri* | Connor2015 | 99.64 |
| ERR832494 | *S. flexneri* | Connor2015 | 98.45 |
| ERR024604 | *S. sonnei* | Holt2012 | 98.33 |
| ERR024605 | *S. sonnei* | Holt2012 | 99.12 |
| ERR024606 | *S. sonnei* | Holt2012 | 99.44 |
| ERR024607 | *S. sonnei* | Holt2012 | 98.25 |
| ERR024608 | *S. sonnei* | Holt2012 | 99.36 |
| ERR024609 | *S. sonnei* | Holt2012 | 99.52 |
| ERR024610 | *S. sonnei* | Holt2012 | 98.65 |
| ERR024611 | *S. sonnei* | Holt2012 | 99.44 |
| ERR024612 | *S. sonnei* | Holt2012 | 99.48 |
| ERR024616 | *S. sonnei* | Holt2012 | 98.45 |
| ERR024617 | *S. sonnei* | Holt2012 | 99.24 |
| ERR024618 | *S. sonnei* | Holt2012 | 99.16 |
| ERR024619 | *S. sonnei* | Holt2012 | 74.49 |
| ERR024620 | *S. sonnei* | Holt2012 | 42.66 |
| ERR024621 | *S. sonnei* | Holt2012 | 5.65 |
| ERR024622 | *S. sonnei* | Holt2012 | 99.52 |

| | | | |
|---|---|---|---|
| ERR024625 | *S. sonnei* | Holt2012 | 99.24 |
| ERR024626 | *S. sonnei* | Holt2012 | 99.64 |
| ERR024627 | *S. sonnei* | Holt2012 | 99.44 |
| ERR025682 | *S. sonnei* | Holt2012 | 99.32 |
| ERR025683 | *S. sonnei* | Holt2012 | 99.40 |
| ERR025685 | *S. sonnei* | Holt2012 | 99.04 |
| ERR025686 | *S. sonnei* | Holt2012 | 99.48 |
| ERR025687 | *S. sonnei* | Holt2012 | 99.20 |
| ERR025689 | *S. sonnei* | Holt2012 | 98.81 |
| ERR025690 | *S. sonnei* | Holt2012 | 99.08 |
| ERR025691 | *S. sonnei* | Holt2012 | 98.41 |
| ERR025692 | *S. sonnei* | Holt2012 | 98.93 |
| ERR025693 | *S. sonnei* | Holt2012 | 99.64 |
| ERR025695 | *S. sonnei* | Holt2012 | 99.44 |
| ERR025697 | *S. sonnei* | Holt2012 | 99.36 |
| ERR025698 | *S. sonnei* | Holt2012 | 98.85 |
| ERR025699 | *S. sonnei* | Holt2012 | 99.68 |
| ERR025700 | *S. sonnei* | Holt2012 | 98.25 |
| ERR025701 | *S. sonnei* | Holt2012 | 99.20 |
| ERR025702 | *S. sonnei* | Holt2012 | 99.52 |
| ERR025703 | *S. sonnei* | Holt2012 | 99.56 |
| ERR025704 | *S. sonnei* | Holt2012 | 99.20 |
| ERR025705 | *S. sonnei* | Holt2012 | 99.48 |
| ERR025706 | *S. sonnei* | Holt2012 | 99.40 |
| ERR025708 | *S. sonnei* | Holt2012 | 99.12 |
| ERR025709 | *S. sonnei* | Holt2012 | 99.24 |
| ERR025710 | *S. sonnei* | Holt2012 | 99.12 |
| ERR025711 | *S. sonnei* | Holt2012 | 99.52 |
| ERR025712 | *S. sonnei* | Holt2012 | 99.52 |
| ERR025713 | *S. sonnei* | Holt2012 | 99.36 |
| ERR025714 | *S. sonnei* | Holt2012 | 99.60 |
| ERR025715 | *S. sonnei* | Holt2012 | 99.48 |
| ERR025716 | *S. sonnei* | Holt2012 | 98.57 |
| ERR025717 | *S. sonnei* | Holt2012 | 98.69 |
| ERR025718 | *S. sonnei* | Holt2012 | 98.21 |
| ERR025719 | *S. sonnei* | Holt2012 | 99.40 |
| ERR025721 | *S. sonnei* | Holt2012 | 99.44 |
| ERR025724 | *S. sonnei* | Holt2012 | 99.48 |
| ERR025725 | *S. sonnei* | Holt2012 | 99.32 |
| ERR025726 | *S. sonnei* | Holt2012 | 99.40 |
| ERR025727 | *S. sonnei* | Holt2012 | 98.05 |
| ERR025729 | *S. sonnei* | Holt2012 | 99.56 |
| ERR025730 | *S. sonnei* | Holt2012 | 99.36 |
| ERR025731 | *S. sonnei* | Holt2012 | 99.52 |
| ERR025732 | *S. sonnei* | Holt2012 | 99.48 |
| ERR025734 | *S. sonnei* | Holt2012 | 99.08 |
| ERR025735 | *S. sonnei* | Holt2012 | 99.36 |
| ERR025737 | *S. sonnei* | Holt2012 | 99.52 |
| ERR025738 | *S. sonnei* | Holt2012 | 98.33 |
| ERR025741 | *S. sonnei* | Holt2012 | 97.53 |

| | | | |
|---|---|---|---|
| ERR025742 | *S. sonnei* | Holt2012 | 96.74 |
| ERR025743 | *S. sonnei* | Holt2012 | 99.40 |
| ERR025744 | *S. sonnei* | Holt2012 | 98.37 |
| ERR025746 | *S. sonnei* | Holt2012 | 99.52 |
| ERR025747 | *S. sonnei* | Holt2012 | 99.60 |
| ERR025749 | *S. sonnei* | Holt2012 | 97.65 |
| ERR025750 | *S. sonnei* | Holt2012 | 99.28 |
| ERR025751 | *S. sonnei* | Holt2012 | 99.52 |
| ERR025752 | *S. sonnei* | Holt2012 | 98.73 |
| ERR025753 | *S. sonnei* | Holt2012 | 99.04 |
| ERR025754 | *S. sonnei* | Holt2012 | 99.32 |
| ERR025755 | *S. sonnei* | Holt2012 | 99.56 |
| ERR025756 | *S. sonnei* | Holt2012 | 99.56 |
| ERR025758 | *S. sonnei* | Holt2012 | 99.48 |
| ERR025759 | *S. sonnei* | Holt2012 | 99.48 |
| ERR025761 | *S. sonnei* | Holt2012 | 99.24 |
| ERR025762 | *S. sonnei* | Holt2012 | 99.36 |
| ERR025763 | *S. sonnei* | Holt2012 | 99.40 |
| ERR025765 | *S. sonnei* | Holt2012 | 98.09 |
| ERR025767 | *S. sonnei* | Holt2012 | 95.94 |
| ERR025768 | *S. sonnei* | Holt2012 | 97.85 |
| ERR028671 | *S. sonnei* | Holt2012 | 99.44 |
| ERR028672 | *S. sonnei* | Holt2012 | 99.36 |
| ERR028673 | *S. sonnei* | Holt2012 | 99.44 |
| ERR028674 | *S. sonnei* | Holt2012 | 99.44 |
| ERR028675 | *S. sonnei* | Holt2012 | 99.44 |
| ERR028676 | *S. sonnei* | Holt2012 | 99.52 |
| ERR028677 | *S. sonnei* | Holt2012 | 99.36 |
| ERR028678 | *S. sonnei* | Holt2012 | 99.36 |
| ERR028679 | *S. sonnei* | Holt2012 | 96.82 |
| ERR028680 | *S. sonnei* | Holt2012 | 98.97 |
| ERR028681 | *S. sonnei* | Holt2012 | 99.32 |
| ERR028684 | *S. sonnei* | Holt2012 | 99.40 |
| ERR028685 | *S. sonnei* | Holt2012 | 99.36 |
| ERR028686 | *S. sonnei* | Holt2012 | 98.85 |
| ERR028687 | *S. sonnei* | Holt2012 | 98.65 |
| ERR028688 | *S. sonnei* | Holt2012 | 98.45 |
| ERR028689 | *S. sonnei* | Holt2012 | 99.52 |
| ERR028690 | *S. sonnei* | Holt2012 | 98.69 |
| ERR028691 | *S. sonnei* | Holt2012 | 97.77 |
| ERR028692 | *S. sonnei* | Holt2012 | 98.17 |
| ERR028693 | *S. sonnei* | Holt2012 | 99.56 |
| ERR028694 | *S. sonnei* | Holt2012 | 99.12 |
| ERR028695 | *S. sonnei* | Holt2012 | 98.77 |
| ERR028697 | *S. sonnei* | Holt2012 | 99.60 |
| ERR028699 | *S. sonnei* | Holt2012 | 97.77 |
| ERR028700 | *S. sonnei* | Holt2012 | 99.56 |
| ERR028702 | *S. sonnei* | Holt2012 | 96.18 |
| ERR028703 | *S. sonnei* | Holt2012 | 98.25 |
| ERR028704 | *S. sonnei* | Holt2012 | 99.01 |

| | | | |
|---|---|---|---|
| ERR028705 | *S. sonnei* | Holt2012 | 99.48 |
| ERR028706 | *S. sonnei* | Holt2012 | 97.37 |
| ERR316291 | *S. sonnei* | Holt2012 | 99.52 |
| ERR316299 | *S. sonnei* | Holt2012 | 98.73 |
| ERR316306 | *S. sonnei* | Holt2012 | 99.52 |
| S13BD00302 | *S. flexneri* | NRC | 99.04 |
| S13BD00535 | *S. flexneri* | NRC | 98.61 |
| S13BD00702 | *S. flexneri* | NRC | 97.21 |
| S13BD00854 | *S. flexneri* | NRC | 99.28 |
| S13BD01310 | *S. flexneri* | NRC | 99.40 |
| S13BD01692 | *S. flexneri* | NRC | 99.28 |
| S13BD02195 | *S. flexneri* | NRC | 99.36 |
| S13BD02470 | *S. flexneri* | NRC | 98.97 |
| S13BD03084 | *S. flexneri* | NRC | 97.97 |
| S13BD04017 | *S. flexneri* | NRC | 97.65 |
| S13BD04231 | *S. flexneri* | NRC | 97.69 |
| S14BD00799 | *S. flexneri* | NRC | 98.73 |
| S14BD01131 | *S. flexneri* | NRC | 99.12 |
| S14BD01142 | *S. flexneri* | NRC | 99.24 |
| S14BD01714 | *S. flexneri* | NRC | 98.97 |
| S14BD02502 | *S. flexneri* | NRC | 97.73 |
| S14BD02795 | *S. flexneri* | NRC | 97.37 |
| S14BD05234 | *S. flexneri* | NRC | 97.57 |
| S14BD05285 | *S. flexneri* | NRC | 97.77 |
| S15BD00055 | *S. flexneri* | NRC | 97.61 |
| S15BD00457 | *S. flexneri* | NRC | 97.65 |
| S15BD00494 | *S. flexneri* | NRC | 97.65 |
| S15BD02368 | *S. flexneri* | NRC | 97.65 |
| S15BD02726 | *S. flexneri* | NRC | 96.38 |
| S15BD02867 | *S. flexneri* | NRC | 96.74 |
| S15BD04659 | *S. flexneri* | NRC | 98.93 |
| S15BD05228 | *S. flexneri* | NRC | 97.61 |
| S15BD06353 | *S. flexneri* | NRC | 97.49 |
| S15BD06515 | *S. flexneri* | NRC | 98.89 |
| S15BD08204 | *S. flexneri* | NRC | 97.37 |
| S15BD08762 | *S. flexneri* | NRC | 98.21 |
| S15BD09335 | *S. flexneri* | NRC | 99.12 |
| S15BD09453 | *S. flexneri* | NRC | 98.53 |
| S15BD09529 | *S. flexneri* | NRC | 99.01 |
| S16BD00148 | *S. flexneri* | NRC | 99.04 |
| S16BD00463 | *S. flexneri* | NRC | 96.98 |
| S16BD01789 | *S. flexneri* | NRC | 97.65 |
| S16BD01991 | *S. flexneri* | NRC | 97.25 |
| S16BD02637 | *S. flexneri* | NRC | 97.13 |
| S16BD02856 | *S. flexneri* | NRC | 73.02 |
| S16BD03590 | *S. flexneri* | NRC | 2.87 |
| S16BD03881 | *S. flexneri* | NRC | 6.13 |
| S16BD04652 | *S. flexneri* | NRC | 2.07 |
| S16BD07706 | *S. flexneri* | NRC | 0.88 |
| S17BD00498 | *S. flexneri* | NRC | 0.12 |

| | | | |
|---|---|---|---|
| S17BD00886 | *S. flexneri* | NRC | 0.60 |
| S17BD02190 | *S. flexneri* | NRC | 0.52 |
| S17BD02300 | *S. flexneri* | NRC | 0.00 |
| S17BD03514 | *S. flexneri* | NRC | 0.20 |
| S17BD04033 | *S. flexneri* | NRC | 0.08 |
| S17BD04223 | *S. flexneri* | NRC | 0.00 |
| S17BD04752 | *S. flexneri* | NRC | 0.00 |
| S17BD06364 | *S. flexneri* | NRC | 0.00 |
| S17BD07654 | *S. flexneri* | NRC | 0.00 |
| S17BD08054 | *S. flexneri* | NRC | 0.00 |
| S17BD08194 | *S. flexneri* | NRC | 0.00 |
| S17BD08237 | *S. flexneri* | NRC | 0.00 |
| S18BD00005 | *S. flexneri* | NRC | 0.00 |
| S18BD00006 | *S. flexneri* | NRC | 0.00 |
| S18BD01109 | *S. flexneri* | NRC | 0.00 |
| S18BD01126 | *S. flexneri* | NRC | 0.00 |
| S18BD01471 | *S. flexneri* | NRC | 0.00 |
| S13BD00834 | *S. sonnei* | NRC | 97.73 |
| S13BD00906 | *S. sonnei* | NRC | 99.40 |
| S13BD02106 | *S. sonnei* | NRC | 99.40 |
| S13BD02107 | *S. sonnei* | NRC | 99.20 |
| S13BD02670 | *S. sonnei* | NRC | 99.56 |
| S13BD02671 | *S. sonnei* | NRC | 98.37 |
| S13BD03196 | *S. sonnei* | NRC | 98.57 |
| S13BD03288 | *S. sonnei* | NRC | 99.01 |
| S13BD03539 | *S. sonnei* | NRC | 99.44 |
| S13BD04360 | *S. sonnei* | NRC | 98.37 |
| S14BD00913 | *S. sonnei* | NRC | 98.33 |
| S14BD03054 | *S. sonnei* | NRC | 98.21 |
| S14BD03397 | *S. sonnei* | NRC | 98.33 |
| S14BD03573 | *S. sonnei* | NRC | 97.25 |
| S14BD04882 | *S. sonnei* | NRC | 95.30 |
| S14BD04971 | *S. sonnei* | NRC | 99.32 |
| S14BD05043 | *S. sonnei* | NRC | 99.52 |
| S15BD00569 | *S. sonnei* | NRC | 99.36 |
| S15BD00797 | *S. sonnei* | NRC | 99.32 |
| S15BD00889 | *S. sonnei* | NRC | 99.28 |
| S15BD00932 | *S. sonnei* | NRC | 99.36 |
| S15BD01283 | *S. sonnei* | NRC | 99.44 |
| S15BD02324 | *S. sonnei* | NRC | 99.48 |
| S15BD02325 | *S. sonnei* | NRC | 99.04 |
| S15BD02326 | *S. sonnei* | NRC | 99.60 |
| S15BD02407 | *S. sonnei* | NRC | 99.56 |
| S15BD02515 | *S. sonnei* | NRC | 99.40 |
| S15BD02737 | *S. sonnei* | NRC | 99.48 |
| S15BD02979 | *S. sonnei* | NRC | 99.32 |
| S15BD02995 | *S. sonnei* | NRC | 98.09 |
| S15BD03192 | *S. sonnei* | NRC | 99.52 |
| S15BD03801 | *S. sonnei* | NRC | 99.44 |
| S15BD03806 | *S. sonnei* | NRC | 99.64 |

| | | | |
|---|---|---|---|
| S15BD03807 | *S. sonnei* | NRC | 99.56 |
| S15BD03808 | *S. sonnei* | NRC | 99.52 |
| S15BD03850 | *S. sonnei* | NRC | 99.40 |
| S15BD04030 | *S. sonnei* | NRC | 99.40 |
| S15BD04031 | *S. sonnei* | NRC | 99.36 |
| S15BD04165 | *S. sonnei* | NRC | 99.52 |
| S15BD04166 | *S. sonnei* | NRC | 99.40 |
| S15BD04402 | *S. sonnei* | NRC | 99.44 |
| S15BD04792 | *S. sonnei* | NRC | 99.56 |
| S15BD04848 | *S. sonnei* | NRC | 99.40 |
| S15BD05940 | *S. sonnei* | NRC | 99.20 |
| S15BD06471 | *S. sonnei* | NRC | 99.48 |
| S15BD07017 | *S. sonnei* | NRC | 99.40 |
| S15BD07161 | *S. sonnei* | NRC | 99.40 |
| S15BD07522 | *S. sonnei* | NRC | 99.28 |
| S15BD09124 | *S. sonnei* | NRC | 99.44 |
| S15BD09162 | *S. sonnei* | NRC | 99.44 |
| S15BD09356 | *S. sonnei* | NRC | 99.44 |
| S15BD09628 | *S. sonnei* | NRC | 99.44 |
| S15BD09656 | *S. sonnei* | NRC | 99.40 |
| S15BD10104 | *S. sonnei* | NRC | 99.44 |
| S15BD10108 | *S. sonnei* | NRC | 99.40 |
| S15BD10196 | *S. sonnei* | NRC | 99.40 |
| S15BD10296 | *S. sonnei* | NRC | 99.44 |
| S16BD00285 | *S. sonnei* | NRC | 99.40 |
| S16BD00582 | *S. sonnei* | NRC | 99.48 |
| S16BD00590 | *S. sonnei* | NRC | 99.20 |
| S16BD00864 | *S. sonnei* | NRC | 98.05 |
| S16BD01339 | *S. sonnei* | NRC | 99.36 |
| S16BD01740 | *S. sonnei* | NRC | 99.40 |
| S16BD01790 | *S. sonnei* | NRC | 99.40 |
| S16BD02550 | *S. sonnei* | NRC | 99.40 |
| S16BD02574 | *S. sonnei* | NRC | 99.44 |
| S16BD03340 | *S. sonnei* | NRC | 99.40 |
| S16BD04069 | *S. sonnei* | NRC | 99.40 |
| S17BD01977 | *S. sonnei* | NRC | 99.40 |
| S17BD02088 | *S. sonnei* | NRC | 99.48 |
| S17BD02164 | *S. sonnei* | NRC | 99.44 |
| S17BD02526 | *S. sonnei* | NRC | 99.32 |
| S17BD02897 | *S. sonnei* | NRC | 99.32 |
| S17BD03513 | *S. sonnei* | NRC | 99.40 |
| S17BD04438 | *S. sonnei* | NRC | 99.56 |
| S17BD06426 | *S. sonnei* | NRC | 99.44 |
| S17BD06464 | *S. sonnei* | NRC | 99.56 |
| S17BD06759 | *S. sonnei* | NRC | 99.44 |
| S17BD06760 | *S. sonnei* | NRC | 99.44 |
| S17BD06799 | *S. sonnei* | NRC | 99.44 |
| S17BD06801 | *S. sonnei* | NRC | 99.48 |
| S17BD07774 | *S. sonnei* | NRC | 98.85 |
| S17BD07961 | *S. sonnei* | NRC | 99.44 |

| | | | |
|---|---|---|---|
| S18BD00011 | *S. sonnei* | NRC | 95.74 |
| S18BD00105 | *S. sonnei* | NRC | 2.83 |
| S18BD00654 | *S. sonnei* | NRC | 0.48 |
| S18BD01122 | *S. sonnei* | NRC | 0.44 |
| S18BD01231 | *S. sonnei* | NRC | 0.28 |

**Supplementary Table S5:** ***In silico*** **determined serotypes for all** ***S. flexneri*** **samples.**

| Sample | Serotype |
|---|---|
| ERR042796 | Xv |
| ERR042797 | X |
| ERR042799 | 2a |
| ERR042803 | 2a |
| ERR042806 | 2b |
| ERR042810 | 1b |
| ERR042811 | 3a |
| ERR042814 | 2a |
| ERR042816 | Y |
| ERR042819 | 3b |
| ERR042821 | 2a |
| ERR042824 | 2a |
| ERR042825 | 2a |
| ERR042827 | 4a |
| ERR042831 | Y |
| ERR042832 | Y |
| ERR042833 | Y |
| ERR042835 | 1a |
| ERR042837 | 3a |
| ERR042838 | Yv |
| ERR042839 | X |
| ERR042840 | Yv |
| ERR042841 | 1a |
| ERR042842 | 2a |
| ERR042843 | 3a |
| ERR042845 | Xv |
| ERR042849 | 1c |
| ERR042851 | 1b |
| ERR042852 | 2b |
| ERR042853 | 1c |
| ERR042855 | 3a |
| ERR042858 | 4av |
| ERR042860 | 2a |
| ERR042861 | Xv |
| ERR042863 | Yv |
| ERR047236 | 1b |
| ERR047239 | 3a |
| ERR047294 | 1b |
| ERR047297 | 4av |
| ERR047306 | 2a |
| ERR047307 | - |
| ERR047372 | 4av |
| ERR047396 | 3a |
| ERR047406 | 2a |
| ERR048234 | 1c |
| ERR048246 | 2a |
| ERR048259 | 4bv |
| ERR048261 | 1b |

| | |
|---|---|
| ERR048265 | 5b |
| ERR048285 | 3a |
| ERR048286 | 1b |
| ERR048287 | 5a |
| ERR048288 | Y |
| ERR048290 | 1b |
| ERR048295 | 2b |
| ERR048296 | 3a |
| ERR048300 | 3a |
| ERR048302 | 2a |
| ERR048304 | 2a |
| ERR048306 | 2a |
| ERR048311 | Yv |
| ERR048312 | 4av |
| ERR048313 | 2b |
| ERR048315 | 2a |
| ERR048316 | 1b |
| ERR048317 | Y |
| ERR048319 | 1a |
| ERR048320 | X |
| ERR048322 | 2a |
| ERR048329 | Y |
| ERR048331 | Y |
| ERR048332 | X |
| ERR048333 | 4a |
| ERR048334 | Yv |
| ERR048339 | 2b |
| ERR049152 | 3a |
| ERR126958 | 2a |
| ERR127015 | 2a |
| ERR127017 | - |
| ERR127019 | 3a |
| ERR127032 | 1a |
| ERR127034 | 1c |
| ERR127035 | 2a |
| ERR127036 | 2b |
| ERR127037 | 3a |
| ERR127038 | - |
| ERR127039 | - |
| ERR127040 | 4a |
| ERR127041 | 4b |
| ERR127042 | 5b |
| ERR127043 | - |
| ERR127044 | - |
| ERR127046 | X |
| ERR127047 | Y |
| ERR127048 | 4av |
| ERR200344 | 1b |
| ERR200360 | 2a |
| ERR200365 | 2a |

| | |
|---|---|
| ERR200370 | 2a |
| ERR200378 | 2a |
| ERR200379 | 2a |
| ERR200390 | 2b |
| ERR200392 | 2b |
| ERR200393 | 3a |
| ERR200402 | 3a |
| ERR200403 | 3a |
| ERR200405 | 3a |
| ERR200413 | 5a |
| ERR200414 | Yv |
| ERR200415 | Y |
| ERR217012 | Y |
| ERR217013 | Yv |
| ERR217015 | Yv |
| ERR217016 | Y |
| ERR217022 | Y |
| ERR217023 | Yv |
| ERR217024 | Y |
| ERR217026 | Y |
| ERR217028 | Yv |
| ERR217030 | Yv |
| ERR217031 | Y |
| ERR217032 | Yv |
| ERR217033 | 1cv |
| ERR217047 | 1c |
| ERR217080 | 1c |
| ERR217081 | 4av |
| ERR217084 | 4av |
| ERR559526 | 2a |
| ERR832465 | 3b |
| S13BD00302 | 1b |
| S13BD00535 | 2a |
| S13BD00702 | 2a |
| S13BD00854 | 2a |
| S13BD01310 | 3a |
| S13BD01692 | 2a |
| S13BD02195 | 2a |
| S13BD02470 | 3a |
| S13BD03084 | Xv |
| S13BD04017 | 3a |
| S13BD04231 | Y |
| S14BD00799 | 2a |
| S14BD01131 | X |
| S14BD01142 | X |
| S14BD01714 | 3a |
| S14BD02502 | 3a |
| S14BD05234 | 1b |
| S14BD05285 | 2a |
| S15BD00055 | 2a |

| | |
|---|---|
| S15BD00457 | 2a |
| S15BD00494 | 2a |
| S15BD02368 | 2a |
| S15BD02726 | 1c |
| S15BD02867 | 2a |
| S15BD04659 | 2a |
| S15BD05228 | 2a |
| S15BD06353 | 3a |
| S15BD08204 | 3a |
| S15BD08762 | 2a |
| S15BD09335 | 3a |
| S15BD09453 | 1b |
| S16BD00148 | 2a |
| S16BD00463 | 1b |
| S16BD01789 | Xv |
| S16BD01991 | 1c |
| S16BD02637 | 3a |
| S16BD02856 | Xv |
| S16BD03590 | 3b |
| S16BD03881 | Y |
| S16BD04652 | 1c |
| S16BD07706 | 3b |
| S17BD00498 | 2a |
| S17BD00886 | 2a |
| S17BD02190 | 2a |
| S17BD02300 | 2a |
| S17BD03514 | 2a |
| S17BD04033 | Yv |
| S17BD04223 | 2a |
| S17BD04752 | 2a |
| S17BD06364 | 3a |
| S17BD07654 | X |
| S17BD08054 | 2a |
| S17BD08194 | 2a |
| S17BD08237 | 2a |
| S18BD00005 | 2a |
| S18BD00006 | 2a |
| S18BD01109 | Yv |
| S18BD01126 | 2a |
| S18BD01471 | 2a |

The first column lists the sample name, the second column lists the in silico determined serotype.

**Supplementary Table S6: Detected AMR genes for all samples.**

| Species | Sample | Detected AMR genes |
|---|---|---|
| *S. flexneri* | ERR042796 | aadA1_5, dfrA1_10, dfrA1_8, dfrA1_9, mdf(A)_1, sul1_39, sul2_2, tet(A)_4 |
| *S. flexneri* | ERR042797 | mdf(A)_1 |
| *S. flexneri* | ERR042799 | aph(3'')-Ib_5, aph(6)-Id_1, blaOXA-1_1, catA1_1, dfrA1_8, sul2_2, tet(B)_2 |
| *S. flexneri* | ERR042803 | aph(3'')-Ib_5, aph(6)-Id_1, blaOXA-1_1, catA1_1, dfrA1_8, mdf(A)_1, sul2_2, tet(B)_2 |
| *S. flexneri* | ERR042806 | - |
| *S. flexneri* | ERR042810 | aadA1_3, aadA1_5, aph(3'')-Ib_5, aph(6)-Id_1, blaOXA-1_1, catA1_1, dfrA14_4, mdf(A)_1, sul2_2, tet(B)_2 |
| *S. flexneri* | ERR042811 | aadA1_3, aadA1_5, blaOXA-1_1, catA1_1, mdf(A)_1, tet(B)_2 |
| *S. flexneri* | ERR042814 | blaOXA-1_1, catA1_1, dfrA1_8, mdf(A)_1, tet(B)_2 |
| *S. flexneri* | ERR042816 | aph(3'')-Ib_5, aph(6)-Id_1, blaOXA-1_1, catA1_1, dfrA1_8, mdf(A)_1, sul2_2, tet(B)_2 |
| *S. flexneri* | ERR042819 | aadA1_3, aadA1_5, blaOXA-1_1, catA1_1, mdf(A)_1, tet(B)_2 |
| *S. flexneri* | ERR042821 | mdf(A)_1, tet(B)_2 |
| *S. flexneri* | ERR042824 | aph(3'')-Ib_5, aph(6)-Id_1, blaOXA-1_1, catA1_1, dfrA1_8, mdf(A)_1, sul2_2, tet(B)_2 |
| *S. flexneri* | ERR042825 | mdf(A)_1 |
| *S. flexneri* | ERR042827 | aadA1_3, aadA1_5, aph(3'')-Ib_5, aph(6)-Id_1, blaOXA-1_1, catA1_1, dfrA14_4, mdf(A)_1, sul2_2, tet(B)_2 |
| *S. flexneri* | ERR042831 | mdf(A)_1 |
| *S. flexneri* | ERR042832 | catA1_1, mdf(A)_1, tet(B)_2 |
| *S. flexneri* | ERR042833 | aph(3'')-Ib_5, aph(6)-Id_1, blaOXA-1_1, catA1_1, dfrA1_8, mdf(A)_1, sul2_2, tet(B)_2 |
| *S. flexneri* | ERR042835 | aph(3'')-Ib_5, aph(3'')-Ib_5, aph(6)-Id_1, aph(6)-Id_1, dfrA14_4, dfrA14_4, mdf(A)_1, sul2_2, sul2_2 |
| *S. flexneri* | ERR042837 | mdf(A)_1 |
| *S. flexneri* | ERR042838 | mdf(A)_1 |
| *S. flexneri* | ERR042839 | aadA1_4, aph(3'')-Ib_5, aph(6)-Id_1, dfrA1_8, mdf(A)_1, sul2_2 |
| *S. flexneri* | ERR042840 | mdf(A)_1, tet(A)_4 |
| *S. flexneri* | ERR042841 | aadA1_4, aph(3'')-Ib_5, aph(6)-Id_1, blaOXA-1_1, dfrA1_8, mdf(A)_1, sul2_2, tet(B)_2 |
| *S. flexneri* | ERR042842 | aph(3'')-Ib_5, aph(6)-Id_1, dfrA14_4, mdf(A)_1, sul2_2, tet(B)_2 |
| *S. flexneri* | ERR042843 | aph(3'')-Ib_5, aph(6)-Id_4, blaTEM-1B_1, dfrA8_1, mdf(A)_1, sul2_2 |
| *S. flexneri* | ERR042845 | mdf(A)_1, sul2_2, tet(A)_4 |
| *S. flexneri* | ERR042849 | aph(3'')-Ib_5, aph(6)-Id_1, blaOXA-1_1, catA1_1, dfrA1_8, mdf(A)_1, sul2_2, tet(B)_2 |
| *S. flexneri* | ERR042851 | aph(3'')-Ib_5, aph(6)-Id_1, dfrA14_4, mdf(A)_1, sul2_2, tet(B)_2 |
| *S. flexneri* | ERR042852 | mdf(A)_1 |
| *S. flexneri* | ERR042853 | aadA1_3, aadA1_5, blaOXA-1_1, catA1_1, mdf(A)_1, tet(B)_2 |
| *S. flexneri* | ERR042855 | aadA1_3, aadA1_5, aph(3'')-Ib_5, aph(6)-Id_1, blaOXA-1_1, catA1_1, dfrA14_4, mdf(A)_1, sul2_2, tet(B)_2 |
| *S. flexneri* | ERR042858 | aadA1_4, dfrA1_8, mdf(A)_1, sul2_2, tet(B)_2 |
| *S. flexneri* | ERR042860 | aadA1_4, blaOXA-1_1, dfrA1_8, mdf(A)_1 |
| *S. flexneri* | ERR042861 | mdf(A)_1, sul2_2, tet(A)_4 |
| *S. flexneri* | ERR042863 | aph(3'')-Ib_5, aph(6)-Id_1, dfrA14_4, mdf(A)_1, sul2_2, tet(A)_4 |

| | | |
|---|---|---|
| *S. flexneri* | ERR047236 | aadA1_3, aadA1_5, aph(3'')-Ib_5, aph(6)-Id_1, blaOXA-1_1, blaTEM-1B_1, catA1_1, dfrA14_1, mdf(A)_1, qnrS1_1, sul2_2 |
| *S. flexneri* | ERR047239 | aadA1_3, aadA1_5, aph(3'')-Ib_5, aph(6)-Id_1, blaTEM-1B_1, catA1_1, dfrA1_10, dfrA1_8, dfrA1_9, mdf(A)_1, sul2_2, tet(B)_2 |
| *S. flexneri* | ERR047294 | aadA1_3, aadA1_5, aph(3'')-Ib_5, aph(6)-Id_1, blaOXA-1_1, blaTEM-1B_1, catA1_1, mdf(A)_1, sul2_2, tet(B)_2 |
| *S. flexneri* | ERR047297 | aadA1_3, aadA1_5, aph(3'')-Ib_5, aph(6)-Id_1, blaTEM-1B_1, catA1_1, mdf(A)_1, sul1_2, sul2_2, tet(A)_4 |
| *S. flexneri* | ERR047306 | aadA1_3, aadA1_5, aph(3'')-Ib_5, aph(6)-Id_1, blaOXA-1_1, blaTEM-1B_1, catA1_1, mdf(A)_1, sul2_2, tet(B)_2 |
| *S. flexneri* | ERR047307 | aadA2_2, aph(3'')-Ib_5, aph(6)-Id_4, blaTEM-1B_1, dfrA12_8, mdf(A)_1, mph(A)_1, sul1_2, sul2_2, tet(A)_4 |
| *S. flexneri* | ERR047372 | aadA1_3, aadA1_5, aph(3'')-Ib_5, aph(6)-Id_1, blaTEM-1B_1, catA1_1, mdf(A)_1, sul2_2, tet(A)_4 |
| *S. flexneri* | ERR047396 | aadA1_3, aadA1_5, catA1_1, dfrA1_10, dfrA1_8, dfrA1_9, mdf(A)_1, sul2_2, tet(B)_2 |
| *S. flexneri* | ERR047406 | aadA1_3, aadA1_5, aph(3'')-Ib_5, aph(6)-Id_1, blaOXA-1_1, blaTEM-1B_1, catA1_1, mdf(A)_1, sul2_2, tet(B)_2 |
| *S. flexneri* | ERR048234 | aadA1_3, aadA1_5, aph(3'')-Ib_5, aph(6)-Id_1, blaOXA-1_1, blaTEM-1B_1, catA1_1, mdf(A)_1, sul2_2, tet(B)_2 |
| *S. flexneri* | ERR048246 | aph(3'')-Ib_5, aph(6)-Id_1, blaTEM-1B_1, dfrA14_1, mdf(A)_1, sul2_2 |
| *S. flexneri* | ERR048259 | mdf(A)_1 |
| *S. flexneri* | ERR048261 | mdf(A)_1 |
| *S. flexneri* | ERR048265 | mdf(A)_1 |
| *S. flexneri* | ERR048285 | mdf(A)_1 |
| *S. flexneri* | ERR048286 | aadA1_3, aadA1_5, aph(3'')-Ib_5, aph(6)-Id_1, blaOXA-1_1, blaTEM-1B_1, catA1_1, dfrA14_1, mdf(A)_1, sul2_2, tet(B)_2 |
| *S. flexneri* | ERR048287 | aadA1_3, aadA1_5, blaOXA-1_1, catA1_1, dfrA1_8, mdf(A)_1, tet(B)_2 |
| *S. flexneri* | ERR048288 | blaOXA-1_1, catA1_1, dfrA1_8, mdf(A)_1, sul2_2, tet(B)_2 |
| *S. flexneri* | ERR048290 | aph(3'')-Ib_5, aph(6)-Id_1, blaOXA-1_1, catA1_1, dfrA1_8, mdf(A)_1, sul2_2, tet(B)_2 |
| *S. flexneri* | ERR048295 | aph(3'')-Ib_5, aph(6)-Id_1, blaOXA-1_1, catA1_1, dfrA1_8, mdf(A)_1, sul2_2, tet(B)_2 |
| *S. flexneri* | ERR048296 | aph(3'')-Ib_5, aph(6)-Id_1, blaOXA-1_1, catA1_1, dfrA1_8, mdf(A)_1, sul2_2, tet(B)_2 |
| *S. flexneri* | ERR048300 | aadA1_3, aadA1_5, blaTEM-1B_1, mdf(A)_1, sul1_2, sul1_2 |
| *S. flexneri* | ERR048302 | aadA1_4, dfrA1_8, mdf(A)_1 |
| *S. flexneri* | ERR048304 | blaTEM-1B_1, mdf(A)_1 |
| *S. flexneri* | ERR048306 | blaTEM-1B_1, mdf(A)_1 |
| *S. flexneri* | ERR048311 | aadA1_3, aadA1_5, catA1_1, mdf(A)_1 |
| *S. flexneri* | ERR048312 | aadA5_1, aph(3'')-Ib_3, aph(3'')-Ib_4, aph(3'')-Ib_5, aph(6)-Id_1, blaTEM-1B_1, dfrA17_1, mdf(A)_1, sul1_2, tet(B)_2 |
| *S. flexneri* | ERR048313 | blaOXA-1_1, catA1_1, dfrA1_8, mdf(A)_1, tet(B)_2 |
| *S. flexneri* | ERR048315 | aac(3)-IIa_1, aadA5_1, blaOXA-1_1, blaTEM-1B_1, catA1_1, dfrA17_1, dfrA1_8, mdf(A)_1, mph(A)_1, sul1_2, tet(B)_2 |
| *S. flexneri* | ERR048316 | mdf(A)_1, sul2_2, tet(A)_4 |
| *S. flexneri* | ERR048317 | aadA1_3, aadA1_5, aph(3'')-Ib_5, aph(6)-Id_4, blaTEM-1B_1, catA1_1, mdf(A)_1, sul2_2, tet(A)_4 |
| *S. flexneri* | ERR048319 | aph(3'')-Ib_5, aph(6)-Id_1, blaOXA-1_1, catA1_1, dfrA1_8, mdf(A)_1, sul2_2, tet(B)_2 |

| | | |
|---|---|---|
| *S. flexneri* | ERR048320 | aph(3'')-Ib_5, aph(6)-Id_1, blaOXA-1_1, catA1_1, dfrA1_8, mdf(A)_1, sul2_2, tet(B)_2 |
| *S. flexneri* | ERR048322 | aph(3'')-Ib_5, aph(6)-Id_1, blaOXA-1_1, blaTEM-1B_1, catA1_1, dfrA14_4, mdf(A)_1, sul2_2, tet(B)_2 |
| *S. flexneri* | ERR048329 | aph(3'')-Ib_5, aph(6)-Id_1, blaTEM-1B_1, dfrA14_1, mdf(A)_1, sul2_2, tet(A)_4 |
| *S. flexneri* | ERR048331 | aph(3'')-Ib_5, aph(6)-Id_1, blaOXA-1_1, catA1_1, dfrA1_8, mdf(A)_1, sul2_2, tet(B)_2 |
| *S. flexneri* | ERR048332 | mdf(A)_1 |
| *S. flexneri* | ERR048333 | aadA1_4, dfrA1_8, mdf(A)_1 |
| *S. flexneri* | ERR048334 | aadA1_3, aadA1_5, aph(6)-Id_4, blaTEM-1B_1, catA1_1, mdf(A)_1 |
| *S. flexneri* | ERR048339 | aph(3'')-Ib_5, aph(6)-Id_1, blaOXA-1_1, catA1_1, dfrA1_8, mdf(A)_1, sul2_2, tet(B)_2 |
| *S. flexneri* | ERR049152 | aadA1_3, aadA1_5, aph(3'')-Ib_5, aph(6)-Id_1, blaTEM-1B_1, catA1_1, dfrA1_10, dfrA1_8, dfrA1_9, mdf(A)_1, sul2_2, tet(B)_2 |
| *S. flexneri* | ERR126958 | aadA1_4, aph(3'')-Ib_5, aph(6)-Id_1, dfrA1_8, sul2_2, tet(B)_2 |
| *S. flexneri* | ERR127015 | mdf(A)_1 |
| *S. flexneri* | ERR127017 | aph(3'')-Ib_5, aph(6)-Id_1, blaOXA-1_1, catA1_1, dfrA1_8, mdf(A)_1, sul2_2, tet(B)_2 |
| *S. flexneri* | ERR127019 | aph(3'')-Ib_5, aph(6)-Id_1, dfrA14_4, mdf(A)_1, sul2_2 |
| *S. flexneri* | ERR127032 | mdf(A)_1 |
| *S. flexneri* | ERR127034 | blaTEM-1B_1, dfrA14_1 |
| *S. flexneri* | ERR127035 | mdf(A)_1 |
| *S. flexneri* | ERR127036 | aph(3'')-Ib_5, aph(6)-Id_4, mdf(A)_1, sul2_2 |
| *S. flexneri* | ERR127037 | mdf(A)_1 |
| *S. flexneri* | ERR127038 | - |
| *S. flexneri* | ERR127039 | mdf(A)_1 |
| *S. flexneri* | ERR127040 | aadA1_3, aadA1_5, sul1_2 |
| *S. flexneri* | ERR127041 | mdf(A)_1 |
| *S. flexneri* | ERR127042 | mdf(A)_1 |
| *S. flexneri* | ERR127043 | mdf(A)_1 |
| *S. flexneri* | ERR127044 | - |
| *S. flexneri* | ERR127046 | mdf(A)_1 |
| *S. flexneri* | ERR127047 | mdf(A)_1, sul2_2 |
| *S. flexneri* | ERR127048 | aadA1_3, aadA1_5, blaOXA-1_1, catA1_1, dfrA1_8, mdf(A)_1, tet(B)_2 |
| *S. flexneri* | ERR200344 | aadA1_4, dfrA1_8, mdf(A)_1, tet(B)_2 |
| *S. flexneri* | ERR200360 | blaOXA-1_1, catA1_1, dfrA1_8, mdf(A)_1, tet(B)_2 |
| *S. flexneri* | ERR200365 | aadA1_4, aph(3'')-Ib_5, aph(6)-Id_1, dfrA1_8, mdf(A)_1, sul2_2 |
| *S. flexneri* | ERR200370 | aph(3'')-Ib_5, aph(6)-Id_1, dfrA1_8, mdf(A)_1, sul2_2, tet(B)_2 |
| *S. flexneri* | ERR200378 | aadA1_4, aph(3'')-Ib_5, aph(6)-Id_1, blaOXA-1_1, catA1_1, dfrA1_8, mdf(A)_1, sul2_2, tet(B)_2 |
| *S. flexneri* | ERR200379 | aadA1_4, dfrA1_8, mdf(A)_1, tet(B)_2 |
| *S. flexneri* | ERR200390 | aph(3'')-Ib_5, aph(6)-Id_1, blaOXA-1_1, catA1_1, dfrA1_8, mdf(A)_1, sul2_2, tet(B)_2 |
| *S. flexneri* | ERR200392 | blaOXA-1_1, catA1_1, dfrA1_8, mdf(A)_1, tet(B)_2 |
| *S. flexneri* | ERR200393 | blaTEM-1B_1, mdf(A)_1, qnrS1_1, tet(A)_4 |
| *S. flexneri* | ERR200402 | mdf(A)_1 |

| | | |
|---|---|---|
| *S. flexneri* | ERR200403 | aadA1_3, aadA1_5, catA1_1, dfrA1_10, dfrA1_8, dfrA1_9, mdf(A)_1, sul2_2, tet(B)_2 |
| *S. flexneri* | ERR200405 | aph(3'')-Ib_5, aph(6)-Id_4, blaTEM-1B_1, erm(B)_1, mdf(A)_1, mph(A)_1, mph(E)_1, msr(E)_1, sul2_2, tet(A)_4 |
| *S. flexneri* | ERR200413 | aph(3'')-Ib_5, aph(6)-Id_1, blaTEM-1B_1, dfrA14_1, mdf(A)_1, qnrS1_1, sul2_2, tet(A)_4 |
| *S. flexneri* | ERR200414 | aph(3'')-Ib_5, aph(6)-Id_1, blaTEM-1B_1, dfrA14_1, mdf(A)_1, sul2_2, tet(A)_4 |
| *S. flexneri* | ERR200415 | aph(3'')-Ib_5, aph(6)-Id_1, blaTEM-1B_1, dfrA5_1, dfrA5_1, mdf(A)_1, sul1_2, sul1_2, sul2_2, tet(A)_4 |
| *S. flexneri* | ERR217012 | aph(3'')-Ib_5, aph(6)-Id_1, blaTEM-1B_1, dfrA14_1, mdf(A)_1, sul2_2, tet(A)_4 |
| *S. flexneri* | ERR217013 | blaOXA-1_1, catA1_1, dfrA1_8, mdf(A)_1, sul2_2, tet(B)_2 |
| *S. flexneri* | ERR217015 | aph(3'')-Ib_5, aph(6)-Id_1, blaTEM-1B_1, dfrA14_1, mdf(A)_1, sul2_2, tet(A)_4 |
| *S. flexneri* | ERR217016 | aph(3'')-Ib_5, aph(6)-Id_1, blaTEM-1B_1, dfrA14_1, mdf(A)_1, sul2_2, tet(A)_4 |
| *S. flexneri* | ERR217022 | mdf(A)_1 |
| *S. flexneri* | ERR217023 | aph(3'')-Ib_5, aph(6)-Id_1, blaTEM-1B_1, dfrA14_1, mdf(A)_1, qnrS1_1, sul2_2 |
| *S. flexneri* | ERR217024 | aph(3'')-Ib_5, aph(6)-Id_1, blaTEM-1B_1, dfrA14_1, mdf(A)_1, qnrS1_1, sul2_2 |
| *S. flexneri* | ERR217026 | aadA1_3, aadA1_5, aph(3'')-Ib_5, aph(6)-Id_4, blaOXA-1_1, blaTEM-1B_1, catA1_1, dfrA8_1, mdf(A)_1, sul2_2, tet(B)_2 |
| *S. flexneri* | ERR217028 | aadA1_3, aadA1_5, blaOXA-1_1, catA1_1, mdf(A)_1, sul2_2, tet(B)_2 |
| *S. flexneri* | ERR217030 | aph(3'')-Ib_5, aph(6)-Id_1, blaTEM-1B_1, dfrA14_1, mdf(A)_1, sul2_2, tet(A)_4 |
| *S. flexneri* | ERR217031 | aadA1_3, aadA1_5, blaOXA-1_1, catA1_1, mdf(A)_1, tet(B)_2 |
| *S. flexneri* | ERR217032 | aph(3'')-Ib_5, aph(6)-Id_1, blaTEM-1B_1, dfrA14_1, mdf(A)_1, sul2_2, tet(A)_4 |
| *S. flexneri* | ERR217033 | aadA1_3, aadA1_5, blaOXA-1_1, mdf(A)_1, tet(B)_2 |
| *S. flexneri* | ERR217047 | mdf(A)_1 |
| *S. flexneri* | ERR217080 | aph(3'')-Ib_5, aph(6)-Id_1, blaOXA-1_1, blaTEM-1B_1, catA1_1, dfrA1_8, mdf(A)_1, mph(A)_1, sul2_2 |
| *S. flexneri* | ERR217081 | aph(3'')-Ib_5, aph(6)-Id_1, blaOXA-1_1, catA1_1, dfrA1_8, mdf(A)_1, sul2_2, tet(B)_2 |
| *S. flexneri* | ERR217084 | aph(3'')-Ib_5, aph(6)-Id_1, blaOXA-1_1, catA1_1, dfrA1_8, mdf(A)_1, sul2_2, tet(B)_2 |
| *S. flexneri* | ERR559526 | - |
| *S. flexneri* | ERR832465 | mdf(A)_1 |
| *S. flexneri* | S13BD00302 | aadA1_4, aph(3'')-Ib_5, aph(6)-Id_1, blaTEM-1B_1, dfrA14_1, dfrA1_8, mdf(A)_1, qnrS1_1, sul2_2, tet(A)_4 |
| *S. flexneri* | S13BD00535 | aadA2_2, blaOXA-1_1, catA1_1, dfrA12_8, dfrA1_8, mdf(A)_1, mph(A)_1, sul1_2, tet(B)_2 |
| *S. flexneri* | S13BD00702 | aph(3'')-Ib_5, aph(6)-Id_1, blaOXA-1_1, catA1_1, dfrA1_8, mdf(A)_1, sul2_2, tet(B)_2 |
| *S. flexneri* | S13BD00854 | aadA2_2, blaOXA-1_1, catA1_1, dfrA12_8, dfrA1_8, mdf(A)_1, mph(A)_1, sul1_2, tet(A)_4, tet(B)_2 |
| *S. flexneri* | S13BD01310 | aadA1_3, aadA1_5, catA1_1, dfrA1_10, dfrA1_8, dfrA1_9, mdf(A)_1, sul2_2, sul2_20, tet(B)_2 |

| | | |
|---|---|---|
| *S. flexneri* | S13BD01692 | blaOXA-1_1, catA1_1, dfrA1_8, mdf(A)_1, tet(B)_2 |
| *S. flexneri* | S13BD02195 | aph(3'')-Ib_5, aph(6)-Id_1, blaOXA-1_1, catA1_1, dfrA1_8, mdf(A)_1, sul2_2, tet(B)_2 |
| *S. flexneri* | S13BD02470 | aadA1_3, aadA1_5, aph(3'')-Ib_5, aph(6)-Id_1, blaTEM-1B_1, catA1_1, dfrA1_10, dfrA1_8, dfrA1_9, mdf(A)_1, sul2_2, tet(B)_2 |
| *S. flexneri* | S13BD03084 | aph(3'')-Ib_5, aph(6)-Id_1, blaTEM-1B_1, catA1_1, dfrA7_1, mdf(A)_1, sul1_39, sul2_3, tet(A)_4 |
| *S. flexneri* | S13BD04017 | aadA1_3, aadA1_5, aadA5_1, blaOXA-1_1, blaTEM-1B_1, catA1_1, dfrA17_1, erm(B)_1, mdf(A)_1, mph(A)_1, mph(A)_1, sul1_2, tet(B)_2 |
| *S. flexneri* | S13BD04231 | aadA1_4, blaOXA-1_1, catA1_1, dfrA1_8, mdf(A)_1, tet(B)_2 |
| *S. flexneri* | S14BD00799 | blaOXA-1_1, catA1_1, dfrA1_8, mdf(A)_1, tet(B)_2 |
| *S. flexneri* | S14BD01131 | aadA1_3, aadA1_5, blaOXA-1_1, blaTEM-1B_1, catA1_1, erm(B)_1, mdf(A)_1, mph(A)_1, tet(B)_2 |
| *S. flexneri* | S14BD01142 | aadA1_3, aadA1_5, blaOXA-1_1, blaTEM-1B_1, catA1_1, erm(B)_1, mdf(A)_1, mph(A)_1, tet(B)_2 |
| *S. flexneri* | S14BD01714 | aadA1_3, aadA1_5, blaOXA-1_1, catA1_1, mdf(A)_1, tet(B)_2 |
| *S. flexneri* | S14BD02502 | aadA1_3, aadA1_5, aadA5_1, blaCTX-M-137_1, blaCTX-M-27_1, blaOXA-1_1, catA1_1, dfrA17_1, mdf(A)_1, mph(A)_1, sul1_2, tet(B)_2 |
| *S. flexneri* | S14BD05234 | aadA1_4, dfrA1_8, mdf(A)_1 |
| *S. flexneri* | S14BD05285 | aadA1_4, blaOXA-1_1, catA1_1, dfrA1_8, mdf(A)_1, tet(B)_2 |
| *S. flexneri* | S15BD00055 | blaOXA-1_1, catA1_1, dfrA1_8, mdf(A)_1, tet(B)_2 |
| *S. flexneri* | S15BD00457 | aadA1_4, dfrA1_8, mdf(A)_1, tet(B)_2 |
| *S. flexneri* | S15BD00494 | blaOXA-1_1, catA1_1, dfrA1_8, mdf(A)_1, tet(B)_2 |
| *S. flexneri* | S15BD02368 | aph(3'')-Ib_5, aph(6)-Id_1, blaOXA-1_1, catA1_1, dfrA1_8, mdf(A)_1, sul2_2, tet(B)_2 |
| *S. flexneri* | S15BD02726 | aadA5_1, aph(3'')-Ib_5, aph(6)-Id_1, blaTEM-1B_1, dfrA17_1, mdf(A)_1, mph(A)_1, qnrS1_1, sul1_2, sul2_2, tet(B)_2 |
| *S. flexneri* | S15BD02867 | aadA1_4, blaOXA-1_1, catA1_1, dfrA1_8, mdf(A)_1, tet(B)_2 |
| *S. flexneri* | S15BD04659 | blaOXA-1_1, catA1_1, dfrA1_8, mdf(A)_1 |
| *S. flexneri* | S15BD05228 | blaOXA-1_1, catA1_1, dfrA1_8, mdf(A)_1, tet(B)_2 |
| *S. flexneri* | S15BD06353 | aadA1_3, aadA1_5, blaOXA-1_1, blaTEM-1B_1, catA1_1, erm(B)_1, mdf(A)_1, mph(A)_1, tet(B)_2 |
| *S. flexneri* | S15BD08204 | aadA1_3, aadA1_5, blaOXA-1_1, blaTEM-1B_1, catA1_1, erm(B)_1, mdf(A)_1, mph(A)_1, tet(B)_2 |
| *S. flexneri* | S15BD08762 | aadA1_4, blaOXA-1_1, catA1_1, dfrA1_8, mdf(A)_1, tet(B)_2 |
| *S. flexneri* | S15BD09335 | mdf(A)_1 |
| *S. flexneri* | S15BD09453 | aadA1_3, aadA1_5, blaOXA-1_1, catA1_1, mdf(A)_1, tet(B)_2 |
| *S. flexneri* | S16BD00148 | aadA2_2, blaOXA-1_1, catA1_1, dfrA12_8, dfrA1_8, mdf(A)_1, mph(A)_1, sul1_2, tet(A)_4, tet(B)_2 |
| *S. flexneri* | S16BD00463 | aadA1_4, aph(6)-Id_1, aph(6)-Id_2, aph(6)-Id_5, dfrA1_8, mdf(A)_1, qnrS1_1, sul2_19, sul2_2, tet(A)_4 |
| *S. flexneri* | S16BD01789 | mdf(A)_1, sul2_18, tet(A)_4 |
| *S. flexneri* | S16BD01991 | aadA5_1, aph(3'')-Ib_5, aph(6)-Id_1, blaTEM-1B_1, dfrA17_1, mdf(A)_1, sul1_2, sul2_3, tet(B)_2 |
| *S. flexneri* | S16BD02637 | aadA1_3, aadA1_5, blaOXA-1_1, blaTEM-1B_1, catA1_1, erm(B)_1, mdf(A)_1, mph(A)_1, tet(B)_2 |
| *S. flexneri* | S16BD02856 | aph(3'')-Ib_5, aph(6)-Id_4, blaTEM-1B_1, dfrA14_1, mdf(A)_1, mph(A)_1, sul2_2, tet(A)_4 |
| *S. flexneri* | S16BD03590 | mdf(A)_1 |

| | | |
|---|---|---|
| *S. flexneri* | S16BD03881 | aadA1_4, aph(3'')-Ib_5, aph(6)-Id_1, catA1_1, dfrA1_8, mdf(A)_1, sul2_1, sul2_12, sul2_15, sul2_2, sul2_20, sul2_6, tet(B)_2 |
| *S. flexneri* | S16BD04652 | mdf(A)_1, tet(B)_2 |
| *S. flexneri* | S16BD07706 | aadA1_3, aadA1_5, blaOXA-1_1, catA1_1, erm(B)_1, mdf(A)_1, mph(A)_1, tet(B)_2 |
| *S. flexneri* | S17BD00498 | blaOXA-1_1, catA1_1, dfrA1_8, mdf(A)_1, tet(B)_2 |
| *S. flexneri* | S17BD00886 | blaOXA-1_1, catA1_1, dfrA1_8, mdf(A)_1, tet(B)_2 |
| *S. flexneri* | S17BD02190 | aph(3'')-Ib_5, aph(6)-Id_1, blaOXA-1_1, catA1_1, dfrA1_8, mdf(A)_1, sul2_2, tet(B)_2 |
| *S. flexneri* | S17BD02300 | aph(3'')-Ib_5, aph(6)-Id_1, blaOXA-1_1, catA1_1, dfrA1_8, mdf(A)_1, sul2_2, tet(B)_2 |
| *S. flexneri* | S17BD03514 | aadA1_4, aph(3'')-Ib_5, aph(6)-Id_1, blaOXA-1_1, catA1_1, dfrA1_8, mdf(A)_1, sul2_2, tet(B)_2 |
| *S. flexneri* | S17BD04033 | aadA1_3, aadA1_5, aph(3'')-Ib_5, aph(6)-Id_1, blaOXA-1_1, catA1_1, dfrA14_4, mdf(A)_1, sul2_2, tet(B)_2 |
| *S. flexneri* | S17BD04223 | aph(3'')-Ib_5, aph(6)-Id_1, blaOXA-1_1, catA1_1, dfrA1_8, mdf(A)_1, sul2_2, tet(B)_2 |
| *S. flexneri* | S17BD04752 | aph(3'')-Ib_5, aph(6)-Id_1, blaOXA-1_1, catA1_1, dfrA1_8, mdf(A)_1, sul2_2, tet(B)_2 |
| *S. flexneri* | S17BD06364 | mdf(A)_1 |
| *S. flexneri* | S17BD07654 | mdf(A)_1 |
| *S. flexneri* | S17BD08054 | aadA1_3, aadA1_5, aadA5_1, blaOXA-1_1, blaTEM-1B_1, catA1_1, dfrA17_1, erm(B)_1, mdf(A)_1, mph(A)_1, sul1_2, tet(B)_2 |
| *S. flexneri* | S17BD08194 | aph(3'')-Ib_5, aph(6)-Id_1, blaOXA-1_1, catA1_1, dfrA1_8, mdf(A)_1, sul2_2, tet(B)_2 |
| *S. flexneri* | S17BD08237 | aadA1_3, aadA1_5, dfrA1_8, mdf(A)_1 |
| *S. flexneri* | S18BD00005 | aadA1_3, aadA1_5, aadA5_1, blaCTX-M-15_1, dfrA17_1, dfrA1_8, mdf(A)_1, mph(A)_1, qnrS1_1, sul1_2 |
| *S. flexneri* | S18BD00006 | blaOXA-1_1, catA1_1, dfrA1_8, mdf(A)_1, tet(B)_2 |
| *S. flexneri* | S18BD01109 | aph(3'')-Ib_5, aph(6)-Id_4, blaLAP-2_1, blaTEM-1B_1, catA2_1, dfrA14_1, mdf(A)_1, qnrS1_1, sul2_2, tet(A)_4 |
| *S. flexneri* | S18BD01126 | aadA1_3, aadA1_5, aadA5_1, blaOXA-1_1, blaTEM-1B_1, catA1_1, dfrA17_1, erm(B)_1, mdf(A)_1, mph(A)_1, sul1_2 |
| *S. flexneri* | S18BD01471 | aadA1_4, aph(3'')-Ib_5, aph(6)-Id_1, dfrA1_8, mdf(A)_1, sul2_2 |
| *S. sonnei* | ERR024604 | mdf(A)_1 |
| *S. sonnei* | ERR024605 | mdf(A)_1 |
| *S. sonnei* | ERR024606 | aph(3'')-Ib_5, blaTEM-1C_1, dfrA14_4, mdf(A)_1, sul2_2 |
| *S. sonnei* | ERR024607 | mdf(A)_1 |
| *S. sonnei* | ERR024608 | aadA1_4, aph(3'')-Ib_5, aph(6)-Id_1, dfrA1_8, mdf(A)_1, sul2_2, tet(A)_6 |
| *S. sonnei* | ERR024609 | aadA1_4, aph(3'')-Ib_5, aph(6)-Id_1, dfrA1_8, mdf(A)_1, sul2_2, tet(A)_6 |
| *S. sonnei* | ERR024610 | dfrA1_8, mdf(A)_1 |
| *S. sonnei* | ERR024611 | dfrA1_8, mdf(A)_1 |
| *S. sonnei* | ERR024612 | aadA1_4, dfrA1_8, mdf(A)_1 |
| *S. sonnei* | ERR024616 | aac(3)-IId_1, aadA2_2, aph(3'')-Ib_5, aph(3')-Ia_1, aph(6)-Id_1, dfrA12_8, dfrA1_8, mdf(A)_1, mph(A)_1, sul1_2, sul2_2, tet(A)_6 |
| *S. sonnei* | ERR024617 | aadA1_4, aph(3'')-Ib_5, aph(6)-Id_1, dfrA1_8, mdf(A)_1, sul2_2, tet(A)_6 |
| *S. sonnei* | ERR024618 | aac(3)-IV_1, aph(3'')-Ib_5, aph(3')-Ia_1, aph(4)-Ia_1, aph(6)-Id_1, dfrA12_8, dfrA1_8, mdf(A)_1, sul1_2, sul2_2, tet(A)_6 |
| *S. sonnei* | ERR024619 | aadA1_3, aadA1_5, catA1_1, mdf(A)_1, sul1_2, sul1_2, tet(B)_1 |

| | | |
|---|---|---|
| *S. sonnei* | ERR024620 | aadA1_4, aph(3'')-Ib_5, aph(6)-Id_1, dfrA1_8, dfrA5_1, mdf(A)_1, sul1_2, sul2_2, tet(A)_6 |
| *S. sonnei* | ERR024621 | aac(3)-IId_1, aac(6')-Ib3_1, aadA1_4, blaOXA-10_1, catB8_1, dfrA1_8, mdf(A)_1, sul1_2, sul2_2 |
| *S. sonnei* | ERR024622 | aph(3'')-Ib_5, aph(6)-Id_1, dfrA1_8, mdf(A)_1, sul2_2, tet(A)_6 |
| *S. sonnei* | ERR024625 | aadA1_4, aph(3')-Ia_1, blaTEM-1B_1, dfrA12_8, dfrA1_8, mdf(A)_1, mph(A)_1, sul1_2 |
| *S. sonnei* | ERR024626 | aadA1_4, aph(3'')-Ib_5, aph(6)-Id_1, dfrA1_8, mdf(A)_1, sul2_2, tet(A)_6 |
| *S. sonnei* | ERR024627 | aadA1_4, aph(3'')-Ib_5, aph(6)-Id_1, dfrA1_8, mdf(A)_1, sul2_2, tet(A)_6 |
| *S. sonnei* | ERR025682 | aadA1_3, aadA1_5, catA1_1, mdf(A)_1, sul1_2, sul1_2, tet(B)_1 |
| *S. sonnei* | ERR025683 | aadA1_4, dfrA1_8, mdf(A)_1 |
| *S. sonnei* | ERR025685 | aadA1_4, dfrA1_8, mdf(A)_1 |
| *S. sonnei* | ERR025686 | aadA1_3, aadA1_5, catA1_1, mdf(A)_1, sul1_2, sul1_2, tet(B)_1 |
| *S. sonnei* | ERR025687 | aadA1_3, aadA1_5, catA1_1, mdf(A)_1, sul1_2, sul1_2, tet(B)_1 |
| *S. sonnei* | ERR025689 | aadA1_3, aadA1_5, mdf(A)_1, sul1_2, sul1_2 |
| *S. sonnei* | ERR025690 | aadA1_3, aadA1_5, catA1_1, mdf(A)_1, sul1_2, tet(B)_1 |
| *S. sonnei* | ERR025691 | mdf(A)_1 |
| *S. sonnei* | ERR025692 | mdf(A)_1 |
| *S. sonnei* | ERR025693 | aadA1_3, aadA1_5, catA1_1, mdf(A)_1, sul1_2, sul1_2, tet(B)_1 |
| *S. sonnei* | ERR025695 | aadA1_4, aph(3'')-Ib_5, aph(6)-Id_1, dfrA1_8, mdf(A)_1, sul2_2, tet(A)_6 |
| *S. sonnei* | ERR025697 | aadA1_4, aph(3'')-Ib_5, aph(6)-Id_1, dfrA1_8, mdf(A)_1, sul2_2, tet(A)_6 |
| *S. sonnei* | ERR025698 | aadA1_4, aph(3'')-Ib_5, aph(3'')-Ib_5, aph(6)-Id_1, aph(6)-Id_1, dfrA1_8, mdf(A)_1, sul2_2, sul2_2, tet(A)_6, tet(A)_6 |
| *S. sonnei* | ERR025699 | aadA1_4, aph(3'')-Ib_5, aph(6)-Id_1, dfrA1_8, mdf(A)_1, sul2_2, tet(A)_6 |
| *S. sonnei* | ERR025700 | aadA1_4, aph(3'')-Ib_5, aph(6)-Id_4, blaTEM-1B_1, dfrA14_1, dfrA1_8, mdf(A)_1, mph(A)_1, sul2_2, tet(A)_4 |
| *S. sonnei* | ERR025701 | aadA1_4, dfrA1_8, mdf(A)_1 |
| *S. sonnei* | ERR025702 | aadA1_4, dfrA1_8, mdf(A)_1 |
| *S. sonnei* | ERR025703 | aadA1_4, aph(3'')-Ib_5, aph(3'')-Ib_5, aph(6)-Id_1, aph(6)-Id_1, dfrA1_8, mdf(A)_1, sul2_2, sul2_2, tet(A)_6, tet(A)_6 |
| *S. sonnei* | ERR025704 | aadA1_4, dfrA1_8, mdf(A)_1 |
| *S. sonnei* | ERR025705 | aadA1_4, dfrA1_8, mdf(A)_1 |
| *S. sonnei* | ERR025706 | aadA1_4, dfrA1_8, mdf(A)_1 |
| *S. sonnei* | ERR025708 | aadA1_4, aph(3'')-Ib_5, aph(6)-Id_1, dfrA1_8, sul2_2, tet(A)_6 |
| *S. sonnei* | ERR025709 | aph(3'')-Ib_5, aph(6)-Id_1, dfrA1_8, mdf(A)_1, sul2_2, tet(A)_6 |
| *S. sonnei* | ERR025710 | aph(3'')-Ib_5, aph(6)-Id_1, dfrA1_8, mdf(A)_1, sul2_2, tet(A)_6 |
| *S. sonnei* | ERR025711 | aph(3'')-Ib_5, aph(6)-Id_1, dfrA1_8, mdf(A)_1, sul2_2, tet(A)_6 |
| *S. sonnei* | ERR025712 | aadA1_4, dfrA1_8, mdf(A)_1 |
| *S. sonnei* | ERR025713 | aadA1_4, dfrA1_8, mdf(A)_1 |
| *S. sonnei* | ERR025714 | aadA1_4, dfrA1_8, mdf(A)_1 |
| *S. sonnei* | ERR025715 | mdf(A)_1, tet(A)_4 |
| *S. sonnei* | ERR025716 | aph(3')-IIa_2, aph(6)-Ic_1, mdf(A)_1 |
| *S. sonnei* | ERR025717 | aph(3'')-Ib_5, aph(3'')-Ib_5, aph(6)-Id_1, aph(6)-Id_1, dfrA1_8, mdf(A)_1, sul2_2, sul2_2, tet(A)_6, tet(A)_6 |
| *S. sonnei* | ERR025718 | aadA1_4, aph(3'')-Ib_5, aph(3'')-Ib_5, aph(6)-Id_1, aph(6)-Id_1, dfrA1_8, mdf(A)_1, sul2_2, sul2_2, tet(A)_6, tet(A)_6 |
| *S. sonnei* | ERR025719 | aadA1_4, aph(3'')-Ib_5, aph(6)-Id_1, dfrA1_8, mdf(A)_1, sul2_2, tet(A)_6 |
| *S. sonnei* | ERR025721 | aph(3'')-Ib_5, aph(6)-Id_1, dfrA1_8, mdf(A)_1, sul2_18, tet(A)_6 |

| | | |
|---|---|---|
| *S. sonnei* | ERR025724 | mdf(A)_1 |
| *S. sonnei* | ERR025725 | aph(3')-IIa_2, aph(6)-Ic_1, mdf(A)_1 |
| *S. sonnei* | ERR025726 | mdf(A)_1 |
| *S. sonnei* | ERR025727 | mdf(A)_1 |
| *S. sonnei* | ERR025729 | mdf(A)_1 |
| *S. sonnei* | ERR025730 | mdf(A)_1 |
| *S. sonnei* | ERR025731 | mdf(A)_1 |
| *S. sonnei* | ERR025732 | mdf(A)_1 |
| *S. sonnei* | ERR025734 | mdf(A)_1, sul2_2 |
| *S. sonnei* | ERR025735 | mdf(A)_1 |
| *S. sonnei* | ERR025737 | mdf(A)_1 |
| *S. sonnei* | ERR025738 | mdf(A)_1 |
| *S. sonnei* | ERR025741 | mdf(A)_1 |
| *S. sonnei* | ERR025742 | - |
| *S. sonnei* | ERR025743 | mdf(A)_1 |
| *S. sonnei* | ERR025744 | mdf(A)_1 |
| *S. sonnei* | ERR025746 | mdf(A)_1 |
| *S. sonnei* | ERR025747 | - |
| *S. sonnei* | ERR025749 | - |
| *S. sonnei* | ERR025750 | mdf(A)_1 |
| *S. sonnei* | ERR025751 | mdf(A)_1 |
| *S. sonnei* | ERR025752 | mdf(A)_1 |
| *S. sonnei* | ERR025753 | aph(3'')-Ib_5, aph(6)-Id_4, mdf(A)_1, sul2_2 |
| *S. sonnei* | ERR025754 | mdf(A)_1, sul2_2, tet(B)_2 |
| *S. sonnei* | ERR025755 | mdf(A)_1 |
| *S. sonnei* | ERR025756 | mdf(A)_1 |
| *S. sonnei* | ERR025758 | mdf(A)_1 |
| *S. sonnei* | ERR025759 | aadA1_4, aph(3'')-Ib_5, aph(6)-Id_1, dfrA1_8, mdf(A)_1, sul2_2, tet(A)_6 |
| *S. sonnei* | ERR025761 | aadA1_3, aadA1_5, blaOXA-1_1, catA1_1, mdf(A)_1, tet(B)_2 |
| *S. sonnei* | ERR025762 | mdf(A)_1 |
| *S. sonnei* | ERR025763 | blaTEM-1A_1, mdf(A)_1 |
| *S. sonnei* | ERR025765 | dfrA5_1, mdf(A)_1, sul1_2 |
| *S. sonnei* | ERR025767 | aadA1_4, aph(3'')-Ib_5, aph(6)-Id_4, dfrA1_8, mdf(A)_1, sul2_2 |
| *S. sonnei* | ERR025768 | aadA1_4, aph(3'')-Ib_5, aph(6)-Id_1, dfrA1_8, mdf(A)_1, sul2_2, tet(A)_6 |
| *S. sonnei* | ERR028671 | aadA1_4, dfrA1_8, mdf(A)_1 |
| *S. sonnei* | ERR028672 | aph(3'')-Ib_5, aph(6)-Id_4, blaTEM-1B_1, dfrA8_1, mdf(A)_1, sul2_2, tet(B)_2 |
| *S. sonnei* | ERR028673 | aph(3'')-Ib_5, aph(6)-Id_4, blaTEM-1C_1, mdf(A)_1, sul2_2 |
| *S. sonnei* | ERR028674 | blaTEM-1C_1, mdf(A)_1 |
| *S. sonnei* | ERR028675 | aph(6)-Id_4, blaTEM-1C_1, mdf(A)_1 |
| *S. sonnei* | ERR028676 | aadA1_4, aph(3'')-Ib_5, aph(6)-Id_1, dfrA1_8, mdf(A)_1, sul2_2, tet(A)_6 |
| *S. sonnei* | ERR028677 | dfrA1_8, mdf(A)_1 |
| *S. sonnei* | ERR028678 | aac(3)-IId_1, aadA24_1, aph(3'')-Ib_5, aph(6)-Id_1, blaCTX-M-15_1, blaTEM-1B_1, dfrA1_8, mdf(A)_1, sul2_2, tet(A)_6 |
| *S. sonnei* | ERR028679 | blaTEM-1B_1, dfrA1_8, mdf(A)_1 |
| *S. sonnei* | ERR028680 | aph(3'')-Ib_5, aph(6)-Id_4, blaTEM-1B_1, dfrA8_1, mdf(A)_1, sul2_2 |

| | | |
|---|---|---|
| *S. sonnei* | ERR028681 | aadA1_4, aph(3'')-Ib_5, aph(6)-Id_1, dfrA1_8, mdf(A)_1, sul2_2, tet(A)_6 |
| *S. sonnei* | ERR028684 | aadA1_4, aph(3'')-Ib_5, aph(6)-Id_1, dfrA1_8, mdf(A)_1, sul2_2, tet(A)_6 |
| *S. sonnei* | ERR028685 | aadA1_4, dfrA1_8, mdf(A)_1, sul2_2, tet(B)_2 |
| *S. sonnei* | ERR028686 | aadA1_4, dfrA1_8, mdf(A)_1, sul2_2, tet(B)_2 |
| *S. sonnei* | ERR028687 | aadA1_4, dfrA1_8, mdf(A)_1, sul2_2, tet(B)_2 |
| *S. sonnei* | ERR028688 | mdf(A)_1 |
| *S. sonnei* | ERR028689 | aadA1_4, aph(3'')-Ib_5, aph(6)-Id_1, dfrA1_8, mdf(A)_1, sul2_2, tet(A)_6 |
| *S. sonnei* | ERR028690 | aadA1_4, aph(3'')-Ib_5, aph(6)-Id_1, dfrA1_8, mdf(A)_1, sul2_2, tet(A)_4 |
| *S. sonnei* | ERR028691 | blaTEM-1A_1, mdf(A)_1 |
| *S. sonnei* | ERR028692 | mdf(A)_1 |
| *S. sonnei* | ERR028693 | aadA1_3, aadA1_5, blaOXA-1_1, catA1_1, mdf(A)_1, tet(B)_2 |
| *S. sonnei* | ERR028694 | aadA1_4, aph(3'')-Ib_5, blaTEM-1C_1, dfrA1_8, mdf(A)_1, sul2_2, tet(A)_6 |
| *S. sonnei* | ERR028695 | aadA1_4, aph(3'')-Ib_5, aph(6)-Id_1, dfrA1_8, mdf(A)_1, sul2_2, tet(A)_6 |
| *S. sonnei* | ERR028697 | aadA1_4, aph(3'')-Ib_5, aph(6)-Id_1, dfrA1_8, mdf(A)_1, sul2_2, tet(A)_6 |
| *S. sonnei* | ERR028699 | aadA1_4, dfrA1_8, mdf(A)_1 |
| *S. sonnei* | ERR028700 | aadA1_4, aph(3'')-Ib_5, aph(6)-Id_1, blaTEM-1B_1, dfrA1_8, mdf(A)_1, sul2_2, tet(A)_4, tet(B)_2 |
| *S. sonnei* | ERR028702 | aadA1_4, dfrA1_8, mdf(A)_1, sul2_2, tet(B)_2 |
| *S. sonnei* | ERR028703 | aadA1_4, dfrA1_8, mdf(A)_1, sul2_2, tet(B)_2 |
| *S. sonnei* | ERR028704 | aadA1_4, dfrA1_8, mdf(A)_1, sul2_2, tet(B)_2 |
| *S. sonnei* | ERR028705 | aph(3'')-Ib_5, aph(6)-Id_1, dfrA1_8, mdf(A)_1, sul2_2, tet(A)_6 |
| *S. sonnei* | ERR028706 | mdf(A)_1 |
| *S. sonnei* | ERR316291 | mdf(A)_1 |
| *S. sonnei* | ERR316299 | mdf(A)_1 |
| *S. sonnei* | ERR316306 | mdf(A)_1 |
| *S. sonnei* | ERR586833 | aph(3'')-Ib_5, aph(6)-Id_1, dfrA1_8, mdf(A)_1, sul2_2, tet(A)_6 |
| *S. sonnei* | ERR586837 | aph(3'')-Ib_5, aph(6)-Id_1, dfrA1_8, mdf(A)_1, sul2_2, tet(A)_6 |
| *S. sonnei* | ERR586838 | aph(3'')-Ib_5, aph(6)-Id_1, dfrA1_8, mdf(A)_1, sul2_2, tet(A)_6 |
| *S. sonnei* | ERR591154 | aph(3'')-Ib_5, aph(6)-Id_1, dfrA1_8, mdf(A)_1, sul2_2, tet(A)_6 |
| *S. sonnei* | ERR591156 | aph(3'')-Ib_5, aph(6)-Id_1, dfrA1_8, mdf(A)_1, sul2_2, tet(A)_6 |
| *S. sonnei* | ERR591166 | aph(3'')-Ib_5, aph(6)-Id_1, dfrA1_8, mdf(A)_1, sul2_2, tet(A)_6 |
| *S. sonnei* | ERR591176 | aph(3'')-Ib_5, aph(6)-Id_1, dfrA1_8, mdf(A)_1, sul2_2, tet(A)_6 |
| *S. sonnei* | ERR591179 | aph(3'')-Ib_5, aph(6)-Id_1, dfrA1_8, mdf(A)_1, sul2_2, tet(A)_6 |
| *S. sonnei* | ERR591185 | aph(3'')-Ib_5, aph(6)-Id_1, dfrA1_8, mdf(A)_1, sul2_2, tet(A)_6 |
| *S. sonnei* | ERR591188 | dfrA1_8, mdf(A)_1 |
| *S. sonnei* | ERR591211 | aph(3'')-Ib_5, aph(6)-Id_1, dfrA1_8, mdf(A)_1, sul2_2, tet(A)_6 |
| *S. sonnei* | ERR591218 | aph(3'')-Ib_5, aph(6)-Id_1, dfrA1_8, mdf(A)_1, sul2_2, tet(A)_6 |
| *S. sonnei* | ERR591405 | aph(3'')-Ib_5, aph(6)-Id_1, dfrA1_8, mdf(A)_1, sul2_2, tet(A)_6 |
| *S. sonnei* | ERR592006 | aph(3'')-Ib_5, aph(6)-Id_1, blaTEM-1B_1, dfrA1_8, mdf(A)_1, sul2_2, tet(A)_6 |
| *S. sonnei* | ERR592007 | aph(3'')-Ib_5, aph(6)-Id_1, dfrA1_8, mdf(A)_1, sul2_2, tet(A)_6 |
| *S. sonnei* | ERR592013 | aph(3'')-Ib_5, aph(6)-Id_1, dfrA1_8, mdf(A)_1, sul2_2, tet(A)_6 |
| *S. sonnei* | ERR592030 | aph(3'')-Ib_5, aph(6)-Id_1, dfrA1_8, mdf(A)_1, sul2_2, tet(A)_6 |
| *S. sonnei* | ERR592189 | aph(3'')-Ib_5, aph(6)-Id_1, dfrA1_8, mdf(A)_1, sul2_2, tet(A)_6 |
| *S. sonnei* | ERR592269 | aph(3'')-Ib_5, aph(6)-Id_1, dfrA1_8, mdf(A)_1, sul2_2, tet(A)_6 |
| *S. sonnei* | ERR592270 | aph(3'')-Ib_5, aph(6)-Id_1, dfrA1_8, mdf(A)_1, sul2_2, tet(A)_6 |

| | | |
|---|---|---|
| *S. sonnei* | ERR592449 | aph(3'')-Ib_5, aph(6)-Id_1, dfrA1_8, mdf(A)_1, sul2_2, tet(A)_6 |
| *S. sonnei* | ERR708271 | aph(3'')-Ib_5, aph(6)-Id_1, dfrA1_8, mdf(A)_1, sul2_2, tet(A)_6 |
| *S. sonnei* | ERR708272 | aph(3'')-Ib_5, aph(6)-Id_1, dfrA1_8, mdf(A)_1, sul2_2, tet(A)_6 |
| *S. sonnei* | ERR708273 | aph(3'')-Ib_5, aph(6)-Id_1, dfrA1_8, mdf(A)_1, sul2_2, tet(A)_6 |
| *S. sonnei* | ERR708274 | aph(3'')-Ib_5, aph(6)-Id_1, dfrA1_8, mdf(A)_1, sul2_2, tet(A)_6 |
| *S. sonnei* | ERR708275 | dfrA1_8, mdf(A)_1 |
| *S. sonnei* | ERR708276 | aph(3'')-Ib_5, aph(6)-Id_1, dfrA1_8, mdf(A)_1, sul2_2, tet(A)_6 |
| *S. sonnei* | ERR708277 | dfrA1_8, mdf(A)_1 |
| *S. sonnei* | ERR708278 | aph(3'')-Ib_5, aph(6)-Id_1, dfrA1_8, mdf(A)_1, sul2_2, tet(A)_6 |
| *S. sonnei* | ERR708279 | aph(3'')-Ib_5, aph(6)-Id_1, dfrA1_8, mdf(A)_1, sul2_2, tet(A)_6 |
| *S. sonnei* | ERR708280 | aph(3'')-Ib_5, aph(6)-Id_1, dfrA1_8, mdf(A)_1, sul2_2, tet(A)_6 |
| *S. sonnei* | ERR708281 | aph(3'')-Ib_5, aph(6)-Id_4, dfrA1_8, mdf(A)_1, sul2_2, tet(A)_6 |
| *S. sonnei* | ERR708282 | dfrA1_8, mdf(A)_1 |
| *S. sonnei* | ERR708283 | aph(3'')-Ib_5, aph(6)-Id_1, dfrA1_8, mdf(A)_1, sul2_2, tet(A)_6 |
| *S. sonnei* | ERR708284 | aph(3'')-Ib_5, aph(6)-Id_1, dfrA1_8, mdf(A)_1, sul2_2, tet(A)_6 |
| *S. sonnei* | ERR708285 | aph(3'')-Ib_5, aph(6)-Id_1, dfrA1_8, mdf(A)_1, sul2_2, tet(A)_6 |
| *S. sonnei* | ERR708286 | aph(3'')-Ib_5, aph(6)-Id_1, blaCTX-M-15_1, blaTEM-1B_1, dfrA1_8, mdf(A)_1, sul2_2, tet(A)_6 |
| *S. sonnei* | ERR708287 | aph(3'')-Ib_5, aph(6)-Id_1, dfrA1_8, mdf(A)_1, sul2_2, tet(A)_6 |
| *S. sonnei* | ERR708288 | aph(3'')-Ib_5, aph(6)-Id_1, dfrA1_8, mdf(A)_1, sul2_2, tet(A)_6 |
| *S. sonnei* | ERR708289 | aph(3'')-Ib_5, aph(6)-Id_1, dfrA1_8, mdf(A)_1, sul2_2, tet(A)_6 |
| *S. sonnei* | ERR708290 | aph(3'')-Ib_5, aph(6)-Id_1, dfrA1_8, mdf(A)_1, sul2_2, tet(A)_6 |
| *S. sonnei* | S13BD00834 | aph(3'')-Ib_5, aph(6)-Id_1, dfrA1_8, mdf(A)_1, sul2_2, tet(A)_6 |
| *S. sonnei* | S13BD00906 | aph(3'')-Ib_5, aph(6)-Id_1, dfrA1_8, mdf(A)_1, sul2_2, tet(A)_6 |
| *S. sonnei* | S13BD02106 | aph(3'')-Ib_5, aph(6)-Id_1, dfrA1_8, mdf(A)_1, sul2_2, tet(A)_6 |
| *S. sonnei* | S13BD02107 | aph(3'')-Ib_5, aph(6)-Id_1, dfrA1_8, mdf(A)_1, sul2_2, tet(A)_6 |
| *S. sonnei* | S13BD02670 | aph(3'')-Ib_5, aph(6)-Id_1, dfrA1_8, mdf(A)_1, sul2_2, tet(A)_6 |
| *S. sonnei* | S13BD02671 | aph(3'')-Ib_5, aph(6)-Id_1, dfrA1_8, mdf(A)_1, sul2_2, tet(A)_6 |
| *S. sonnei* | S13BD03196 | aph(3'')-Ib_5, aph(6)-Id_1, dfrA1_8, mdf(A)_1, sul2_2, tet(A)_6 |
| *S. sonnei* | S13BD03288 | aadA1_4, dfrA1_8, mdf(A)_1 |
| *S. sonnei* | S13BD03539 | aph(3'')-Ib_5, aph(6)-Id_1, dfrA1_8, mdf(A)_1, sul2_2, tet(A)_6 |
| *S. sonnei* | S13BD04360 | aph(3'')-Ib_5, aph(6)-Id_1, dfrA1_8, mdf(A)_1, sul2_2, tet(A)_6 |
| *S. sonnei* | S14BD00913 | aph(3'')-Ib_5, aph(6)-Id_1, dfrA1_8, mdf(A)_1, sul2_2, tet(A)_6 |
| *S. sonnei* | S14BD03054 | aph(3'')-Ib_5, aph(6)-Id_1, dfrA1_8, mdf(A)_1, sul2_2, tet(A)_6 |
| *S. sonnei* | S14BD03397 | aph(3'')-Ib_5, aph(6)-Id_1, dfrA1_8, mdf(A)_1, sul2_2, tet(A)_6 |
| *S. sonnei* | S14BD03573 | aph(3'')-Ib_5, aph(6)-Id_1, dfrA1_8, mdf(A)_1, sul2_2, tet(A)_6 |
| *S. sonnei* | S14BD04882 | aadA1_4, dfrA1_8, mdf(A)_1 |
| *S. sonnei* | S14BD04971 | aph(3'')-Ib_5, aph(6)-Id_1, dfrA1_8, mdf(A)_1, sul2_2, tet(A)_6 |
| *S. sonnei* | S14BD05043 | aph(3'')-Ib_5, aph(6)-Id_1, dfrA1_8, mdf(A)_1, sul2_2, tet(A)_6 |
| *S. sonnei* | S15BD00569 | dfrA1_8, mdf(A)_1 |
| *S. sonnei* | S15BD00797 | aph(3'')-Ib_5, aph(6)-Id_1, dfrA1_8, mdf(A)_1, sul2_2, tet(A)_6 |
| *S. sonnei* | S15BD00889 | aph(3'')-Ib_5, aph(6)-Id_1, dfrA1_8, mdf(A)_1, sul2_2, tet(A)_6 |
| *S. sonnei* | S15BD00932 | dfrA1_8, mdf(A)_1 |
| *S. sonnei* | S15BD01283 | dfrA1_8, mdf(A)_1 |
| *S. sonnei* | S15BD02324 | dfrA1_8, mdf(A)_1 |
| *S. sonnei* | S15BD02325 | dfrA1_8, mdf(A)_1 |

| | | |
|---|---|---|
| *S. sonnei* | S15BD02326 | dfrA1_8, mdf(A)_1 |
| *S. sonnei* | S15BD02407 | aph(3'')-Ib_5, aph(6)-Id_1, blaCTX-M-15_1, dfrA1_8, mdf(A)_1, qnrS1_1, sul2_2, tet(A)_6 |
| *S. sonnei* | S15BD02515 | aadA1_4, blaTEM-1B_1, dfrA1_8, erm(B)_1, mdf(A)_1, mph(A)_1 |
| *S. sonnei* | S15BD02737 | aph(3'')-Ib_5, aph(6)-Id_1, dfrA1_8, mdf(A)_1, sul2_2, tet(A)_6 |
| *S. sonnei* | S15BD02979 | aph(3'')-Ib_5, aph(6)-Id_1, dfrA1_8, mdf(A)_1, sul2_2, tet(A)_6 |
| *S. sonnei* | S15BD02995 | dfrA1_8, mdf(A)_1 |
| *S. sonnei* | S15BD03192 | aadA1_4, aph(3'')-Ib_5, aph(6)-Id_1, dfrA1_8, mdf(A)_1, sul2_2, tet(A)_6 |
| *S. sonnei* | S15BD03801 | aph(3'')-Ib_5, aph(6)-Id_1, dfrA1_8, mdf(A)_1, sul2_2, tet(A)_6 |
| *S. sonnei* | S15BD03806 | aph(3'')-Ib_5, aph(6)-Id_1, dfrA1_8, mdf(A)_1, sul2_2, tet(A)_6 |
| *S. sonnei* | S15BD03807 | aph(3'')-Ib_5, aph(6)-Id_1, dfrA1_8, mdf(A)_1, sul2_2, tet(A)_6 |
| *S. sonnei* | S15BD03808 | aph(3'')-Ib_5, aph(6)-Id_1, dfrA1_8, mdf(A)_1, sul2_2, tet(A)_6 |
| *S. sonnei* | S15BD03850 | aph(3'')-Ib_5, aph(6)-Id_1, dfrA1_8, mdf(A)_1, sul2_2, tet(A)_6 |
| *S. sonnei* | S15BD04030 | aph(3'')-Ib_5, aph(6)-Id_1, dfrA1_8, mdf(A)_1, sul2_2, tet(A)_6 |
| *S. sonnei* | S15BD04031 | aac(3)-IIa_1, aph(3'')-Ib_5, aph(6)-Id_1, blaDHA-1_1, dfrA17_1, dfrA1_8, mdf(A)_1, mph(A)_1, qnrB4_1, sul1_2, sul2_2, tet(A)_6 |
| *S. sonnei* | S15BD04165 | aph(3'')-Ib_5, aph(6)-Id_1, dfrA1_8, mdf(A)_1, sul2_2, tet(A)_6 |
| *S. sonnei* | S15BD04166 | aph(3'')-Ib_5, aph(6)-Id_1, dfrA1_8, mdf(A)_1, sul2_2, tet(A)_6 |
| *S. sonnei* | S15BD04402 | aadA1_4, dfrA1_8, mdf(A)_1 |
| *S. sonnei* | S15BD04792 | aadA1_4, aph(3'')-Ib_5, aph(6)-Id_1, blaDHA-1_1, dfrA17_1, dfrA1_8, mdf(A)_1, mph(A)_1, qnrB4_1, sul1_2, sul2_2, tet(A)_6 |
| *S. sonnei* | S15BD04848 | aadA5_1, aph(3'')-Ib_5, aph(6)-Id_1, blaTEM-1B_1, dfrA17_1, dfrA1_8, erm(B)_1, mdf(A)_1, mph(A)_1, sul1_2, sul2_2, tet(A)_6 |
| *S. sonnei* | S15BD05940 | aph(3'')-Ib_5, aph(6)-Id_1, dfrA1_8, mdf(A)_1, sul2_2, tet(A)_6 |
| *S. sonnei* | S15BD06471 | aph(3'')-Ib_5, aph(6)-Id_1, dfrA1_8, mdf(A)_1, qnrB19_1, sul2_2, tet(A)_6 |
| *S. sonnei* | S15BD07017 | aph(3'')-Ib_5, aph(6)-Id_1, dfrA1_8, mdf(A)_1, sul2_2, tet(A)_6 |
| *S. sonnei* | S15BD07161 | aadA5_1, blaTEM-1B_1, dfrA17_1, dfrA1_8, erm(B)_1, mdf(A)_1, mph(A)_1, sul1_2 |
| *S. sonnei* | S15BD07522 | aph(3'')-Ib_5, aph(6)-Id_1, dfrA1_8, mdf(A)_1, sul2_2, tet(A)_6 |
| *S. sonnei* | S15BD09124 | dfrA1_8, mdf(A)_1 |
| *S. sonnei* | S15BD09162 | aph(3'')-Ib_5, aph(6)-Id_1, blaCTX-M-55_1, dfrA1_8, mdf(A)_1, sul2_2, tet(A)_6 |
| *S. sonnei* | S15BD09356 | aph(3'')-Ib_5, aph(6)-Id_1, dfrA1_8, mdf(A)_1, sul2_2, tet(A)_6 |
| *S. sonnei* | S15BD09628 | aadA1_4, aadA5_1, blaTEM-1B_1, dfrA17_1, dfrA1_8, mdf(A)_1, mph(A)_1, sul1_2, tet(B)_2 |
| *S. sonnei* | S15BD09656 | aph(3'')-Ib_5, aph(6)-Id_1, dfrA1_8, mdf(A)_1, sul2_2, tet(A)_6 |
| *S. sonnei* | S15BD10104 | aph(3'')-Ib_5, aph(6)-Id_1, dfrA1_8, mdf(A)_1, sul2_2, tet(A)_6 |
| *S. sonnei* | S15BD10108 | aph(3'')-Ib_5, aph(6)-Id_1, dfrA1_8, mdf(A)_1, sul2_2, tet(A)_6 |
| *S. sonnei* | S15BD10196 | aph(3'')-Ib_5, aph(6)-Id_1, dfrA1_8, mdf(A)_1, sul2_2, tet(A)_6 |
| *S. sonnei* | S15BD10296 | aadA1_4, aph(3'')-Ib_5, aph(6)-Id_1, dfrA1_8, mdf(A)_1, sul2_2, tet(A)_6 |
| *S. sonnei* | S16BD00285 | aph(3'')-Ib_5, aph(6)-Id_1, blaCTX-M-15_1, dfrA1_8, mdf(A)_1, qnrS1_1, sul2_2, tet(A)_6 |
| *S. sonnei* | S16BD00582 | aph(3'')-Ib_5, aph(6)-Id_1, dfrA1_8, mdf(A)_1, sul2_2, tet(A)_6 |
| *S. sonnei* | S16BD00590 | aph(3'')-Ib_5, aph(6)-Id_1, dfrA1_8, mdf(A)_1, sul2_2, tet(A)_6 |
| *S. sonnei* | S16BD01339 | aadA1_4, aph(3'')-Ib_5, aph(6)-Id_1, dfrA1_8, mdf(A)_1, sul2_2, tet(A)_6 |
| *S. sonnei* | S16BD01740 | aadA5_1, aph(3'')-Ib_5, aph(6)-Id_4, blaTEM-1B_1, dfrA17_1, dfrA1_8, mdf(A)_1, mph(A)_1, sul1_2, sul2_2, tet(A)_6 |
| *S. sonnei* | S16BD01790 | aph(3'')-Ib_5, aph(6)-Id_1, dfrA1_8, mdf(A)_1, sul2_2, tet(A)_6 |

| | | |
|---|---|---|
| *S. sonnei* | S16BD02574 | aph(3'')-Ib_5, aph(6)-Id_1, dfrA1_8, mdf(A)_1, sul2_2, tet(A)_6 |
| *S. sonnei* | S16BD03340 | aph(3'')-Ib_5, aph(6)-Id_1, dfrA1_8, mdf(A)_1, sul2_2, tet(A)_6 |
| *S. sonnei* | S16BD04069 | aph(3'')-Ib_5, aph(6)-Id_1, dfrA1_8, mdf(A)_1, sul2_2, tet(A)_6 |
| *S. sonnei* | S17BD01977 | aadA1_4, dfrA1_8 |
| *S. sonnei* | S17BD02088 | aadA1_4, dfrA1_8, mdf(A)_1 |
| *S. sonnei* | S17BD02164 | aadA1_4, dfrA1_8, mdf(A)_1 |
| *S. sonnei* | S17BD02526 | aadA1_4, dfrA1_8, mdf(A)_1 |
| *S. sonnei* | S17BD02897 | dfrA1_8, mdf(A)_1 |
| *S. sonnei* | S17BD03513 | dfrA1_8, mdf(A)_1 |
| *S. sonnei* | S17BD04438 | aadA5_1, aph(3'')-Ib_5, aph(6)-Id_1, blaTEM-1B_1, dfrA17_1, dfrA1_8, erm(B)_1, mdf(A)_1, mph(A)_1, sul1_2, sul2_2, tet(A)_6 |
| *S. sonnei* | S17BD06426 | aadA1_4, aph(3'')-Ib_5, aph(6)-Id_1, dfrA1_8, mdf(A)_1, sul2_2, tet(A)_6 |
| *S. sonnei* | S17BD06464 | aph(3'')-Ib_5, aph(6)-Id_1, dfrA1_8, mdf(A)_1, sul2_2, tet(A)_6 |
| *S. sonnei* | S17BD06759 | aph(3'')-Ib_5, aph(6)-Id_1, dfrA1_8, mdf(A)_1, sul2_2, tet(A)_4 |
| *S. sonnei* | S17BD06760 | aadA5_1, aph(3'')-Ib_5, aph(6)-Id_1, blaTEM-1B_1, dfrA17_1, dfrA1_8, erm(B)_1, mdf(A)_1, mph(A)_1, sul1_2, sul2_2, tet(A)_6 |
| *S. sonnei* | S17BD06799 | aph(3'')-Ib_5, aph(6)-Id_1, dfrA1_8, mdf(A)_1, sul2_2, tet(A)_6 |
| *S. sonnei* | S17BD06801 | aadA5_1, blaTEM-1B_1, dfrA17_1, dfrA1_8, erm(B)_1, mdf(A)_1, mph(A)_1, sul1_2 |
| *S. sonnei* | S17BD07774 | aadA5_1, aph(3'')-Ib_5, aph(6)-Id_1, blaTEM-1B_1, dfrA17_1, dfrA1_8, erm(B)_1, mdf(A)_1, mph(A)_1, sul1_2, sul2_2, tet(A)_6 |
| *S. sonnei* | S17BD07961 | aph(3'')-Ib_5, aph(6)-Id_1, dfrA1_8, mdf(A)_1, sul2_2, tet(A)_6 |
| *S. sonnei* | S18BD00011 | aadA5_1, aph(3'')-Ib_5, aph(6)-Id_1, blaCTX-M-15_1, dfrA17_1, dfrA1_8, mdf(A)_1, mph(A)_1, qnrS1_1, sul1_2, sul2_2, tet(A)_6 |
| *S. sonnei* | S18BD00105 | aadA5_1, aph(3'')-Ib_5, aph(6)-Id_1, blaTEM-1B_1, dfrA17_1, dfrA1_8, erm(B)_1, mdf(A)_1, mph(A)_1, sul1_2, sul2_2, tet(A)_6 |
| *S. sonnei* | S18BD00654 | aadA1_4, aph(3'')-Ib_5, aph(6)-Id_1, dfrA1_8, mdf(A)_1, sul2_2, tet(A)_6 |
| *S. sonnei* | S18BD01122 | dfrA1_8, mdf(A)_1 |
| *S. sonnei* | S18BD01231 | aadA1_4, aadA5_1, blaTEM-1B_1, dfrA17_1, dfrA1_3, dfrA1_8, erm(B)_1, mdf(A)_1, mph(A)_1, sul1_2, sul2_2, tet(B)_2 |
| *S. sonnei* | S18BD01685 | aadA1_4, aadA5_1, blaTEM-1B_1, dfrA17_1, dfrA1_3, dfrA1_8, erm(B)_1, mdf(A)_1, mph(A)_1, sul1_2, sul2_2, tet(B)_2 |
| *S. sonnei* | S18BD01986 | aadA5_1, blaTEM-1B_1, dfrA17_1, dfrA1_8, erm(B)_1, mdf(A)_1, mph(A)_1, sul1_2 |
| *S. sonnei* | S18BD02200 | aadA1_4, aph(3'')-Ib_5, aph(6)-Id_1, dfrA1_8, mdf(A)_1, sul2_2, tet(A)_3, tet(A)_4 |
| *S. sonnei* | SRR1605255 | aph(3'')-Ib_5, aph(6)-Id_1, blaCTX-M-15_1, blaTEM-1B_1, dfrA1_8, mdf(A)_1, sul2_2, tet(A)_6 |
| *S. sonnei* | SRR2081185 | dfrA1_8, mdf(A)_1 |
| *S. sonnei* | SRR2081189 | aph(3'')-Ib_5, aph(6)-Id_1, dfrA1_8, mdf(A)_1, sul2_2, tet(A)_6 |
| *S. sonnei* | SRR2087717 | aph(3'')-Ib_5, aph(6)-Id_1, dfrA1_8, mdf(A)_1, sul2_2, tet(A)_6 |
| *S. sonnei* | SRR2097498 | aph(3'')-Ib_5, aph(6)-Id_1, blaCTX-M-15_1, blaTEM-1B_1, dfrA1_8, mdf(A)_1, sul2_2, tet(A)_6 |
| *S. sonnei* | SRR3473843 | aph(3'')-Ib_5, aph(6)-Id_1, dfrA1_8, mdf(A)_1, sul2_2, tet(A)_6 |
| *S. sonnei* | SRR3473844 | dfrA1_8, mdf(A)_1 |
| *S. sonnei* | SRR3473847 | dfrA1_8, mdf(A)_1 |
| *S. sonnei* | SRR3473848 | aph(3'')-Ib_5, aph(6)-Id_1, dfrA1_8, mdf(A)_1, sul2_2, tet(A)_6 |
| *S. sonnei* | SRR3473849 | aph(3'')-Ib_5, aph(6)-Id_1, dfrA1_8, mdf(A)_1, sul2_2, tet(A)_6 |
| *S. sonnei* | SRR3473850 | aph(3'')-Ib_5, aph(6)-Id_1, dfrA1_8, mdf(A)_1, sul2_2, tet(A)_6 |

| | | |
|---|---|---|
| *S. sonnei* | SRR3473853 | dfrA1_8, mdf(A)_1 |
| *S. sonnei* | SRR3473854 | aph(3'')-Ib_5, aph(6)-Id_1, dfrA1_8, mdf(A)_1, mph(A)_1, sul2_2, tet(A)_6 |
| *S. sonnei* | SRR3473855 | aph(3'')-Ib_5, aph(6)-Id_1, dfrA1_8, mdf(A)_1, sul2_2, tet(A)_4 |
| *S. sonnei* | SRR3473856 | aph(3'')-Ib_5, aph(6)-Id_1, dfrA1_8, mdf(A)_1, sul2_2, tet(A)_6 |
| *S. sonnei* | SRR3473857 | aph(3'')-Ib_5, aph(6)-Id_1, dfrA1_8, sul2_2, tet(A)_6 |
| *S. sonnei* | SRR3473858 | aph(3'')-Ib_5, aph(6)-Id_1, dfrA1_8, mdf(A)_1, sul2_2, tet(A)_6 |
| *S. sonnei* | SRR3473859 | aph(3'')-Ib_5, aph(6)-Id_1, dfrA1_8, mdf(A)_1, sul2_2, tet(A)_6 |
| *S. sonnei* | SRR3473860 | dfrA1_8 |
| *S. sonnei* | SRR3473861 | dfrA1_8, mdf(A)_1 |
| *S. sonnei* | SRR3473862 | dfrA1_8, mdf(A)_1 |
| *S. sonnei* | SRR3474162 | aph(3'')-Ib_5, aph(6)-Id_1, dfrA1_8, mdf(A)_1, sul2_2, tet(A)_6 |
| *S. sonnei* | SRR3474164 | aph(3'')-Ib_5, aph(6)-Id_1, blaTEM-1B_1, dfrA1_8, mdf(A)_1, sul2_2, tet(A)_6 |
| *S. sonnei* | SRR3474165 | aph(3'')-Ib_5, aph(6)-Id_1, blaCTX-M-15_1, blaTEM-1B_1, dfrA1_8, mdf(A)_1, sul2_2, tet(A)_6 |
| *S. sonnei* | SRR3474166 | aph(3'')-Ib_5, aph(6)-Id_1, blaCTX-M-137_1, blaCTX-M-14_1, dfrA1_8, mdf(A)_1, sul2_2, tet(A)_6 |
| *S. sonnei* | SRR3474167 | aph(3'')-Ib_5, aph(6)-Id_1, dfrA1_8, mdf(A)_1, sul2_2, tet(A)_6 |
| *S. sonnei* | SRR3474168 | aph(3'')-Ib_5, aph(6)-Id_1, dfrA1_8, mdf(A)_1, sul2_2, tet(A)_6 |
| *S. sonnei* | SRR3474169 | aph(3'')-Ib_5, aph(6)-Id_1, dfrA1_8, mdf(A)_1, sul2_2 |

The first and second columns list the species and sample names, respectively. The third column lists all AMR genes from the ResFinder database that were detected in the corresponding sample.

**Supplementary Table S7: Detected AMR point mutations for all samples.**

| Species | Sample | Detected AMR point mutations |
|---|---|---|
| *S. flexneri* | ERR042796 | - |
| *S. flexneri* | ERR042797 | - |
| *S. flexneri* | ERR042799 | - |
| *S. flexneri* | ERR042803 | - |
| *S. flexneri* | ERR042806 | - |
| *S. flexneri* | ERR042810 | - |
| *S. flexneri* | ERR042811 | - |
| *S. flexneri* | ERR042814 | - |
| *S. flexneri* | ERR042816 | - |
| *S. flexneri* | ERR042819 | - |
| *S. flexneri* | ERR042821 | - |
| *S. flexneri* | ERR042824 | - |
| *S. flexneri* | ERR042825 | - |
| *S. flexneri* | ERR042827 | - |
| *S. flexneri* | ERR042831 | - |
| *S. flexneri* | ERR042832 | - |
| *S. flexneri* | ERR042833 | - |
| *S. flexneri* | ERR042835 | - |
| *S. flexneri* | ERR042837 | - |
| *S. flexneri* | ERR042838 | - |
| *S. flexneri* | ERR042839 | - |
| *S. flexneri* | ERR042840 | - |
| *S. flexneri* | ERR042841 | - |
| *S. flexneri* | ERR042842 | - |
| *S. flexneri* | ERR042843 | - |
| *S. flexneri* | ERR042845 | - |
| *S. flexneri* | ERR042849 | - |
| *S. flexneri* | ERR042851 | - |
| *S. flexneri* | ERR042852 | - |
| *S. flexneri* | ERR042853 | - |
| *S. flexneri* | ERR042855 | - |
| *S. flexneri* | ERR042858 | - |
| *S. flexneri* | ERR042860 | - |
| *S. flexneri* | ERR042861 | - |
| *S. flexneri* | ERR042863 | - |
| *S. flexneri* | ERR047236 | - |
| *S. flexneri* | ERR047239 | gyrA p.S83L |
| *S. flexneri* | ERR047294 | - |
| *S. flexneri* | ERR047297 | - |
| *S. flexneri* | ERR047306 | - |
| *S. flexneri* | ERR047307 | - |
| *S. flexneri* | ERR047372 | - |
| *S. flexneri* | ERR047396 | - |
| *S. flexneri* | ERR047406 | gyrA p.S83L |
| *S. flexneri* | ERR048234 | - |
| *S. flexneri* | ERR048246 | - |
| *S. flexneri* | ERR048259 | - |
| *S. flexneri* | ERR048261 | - |

| | | |
|---|---|---|
| *S. flexneri* | ERR048265 | - |
| *S. flexneri* | ERR048285 | - |
| *S. flexneri* | ERR048286 | - |
| *S. flexneri* | ERR048287 | - |
| *S. flexneri* | ERR048288 | - |
| *S. flexneri* | ERR048290 | - |
| *S. flexneri* | ERR048295 | - |
| *S. flexneri* | ERR048296 | - |
| *S. flexneri* | ERR048300 | - |
| *S. flexneri* | ERR048302 | - |
| *S. flexneri* | ERR048304 | gyrA p.S83L |
| *S. flexneri* | ERR048306 | gyrA p.S83L |
| *S. flexneri* | ERR048311 | - |
| *S. flexneri* | ERR048312 | - |
| *S. flexneri* | ERR048313 | - |
| *S. flexneri* | ERR048315 | - |
| *S. flexneri* | ERR048316 | - |
| *S. flexneri* | ERR048317 | - |
| *S. flexneri* | ERR048319 | gyrA p.S83L, parC p.S80I |
| *S. flexneri* | ERR048320 | gyrA p.D87G, gyrA p.S83L, parC p.S80I |
| *S. flexneri* | ERR048322 | - |
| *S. flexneri* | ERR048329 | - |
| *S. flexneri* | ERR048331 | - |
| *S. flexneri* | ERR048332 | - |
| *S. flexneri* | ERR048333 | - |
| *S. flexneri* | ERR048334 | - |
| *S. flexneri* | ERR048339 | gyrA p.S83L |
| *S. flexneri* | ERR049152 | gyrA p.S83L |
| *S. flexneri* | ERR126958 | - |
| *S. flexneri* | ERR127015 | - |
| *S. flexneri* | ERR127017 | - |
| *S. flexneri* | ERR127019 | - |
| *S. flexneri* | ERR127032 | - |
| *S. flexneri* | ERR127034 | gyrA p.S83L |
| *S. flexneri* | ERR127035 | - |
| *S. flexneri* | ERR127036 | - |
| *S. flexneri* | ERR127037 | - |
| *S. flexneri* | ERR127038 | - |
| *S. flexneri* | ERR127039 | - |
| *S. flexneri* | ERR127040 | - |
| *S. flexneri* | ERR127041 | - |
| *S. flexneri* | ERR127042 | - |
| *S. flexneri* | ERR127043 | - |
| *S. flexneri* | ERR127044 | - |
| *S. flexneri* | ERR127046 | - |
| *S. flexneri* | ERR127047 | - |
| *S. flexneri* | ERR127048 | - |
| *S. flexneri* | ERR200344 | gyrA p.D87N |
| *S. flexneri* | ERR200360 | gyrA p.D87N, gyrA p.S83L, parC p.S80I |
| *S. flexneri* | ERR200365 | gyrA p.D87N, gyrA p.S83L, parC p.S80I |

| | | |
|---|---|---|
| *S. flexneri* | ERR200370 | gyrA p.D87N, gyrA p.S83L, parC p.S80I |
| *S. flexneri* | ERR200378 | gyrA p.D87N, gyrA p.S83L, parC p.S80I |
| *S. flexneri* | ERR200379 | gyrA p.D87N, gyrA p.S83L, parC p.S80I |
| *S. flexneri* | ERR200390 | gyrA p.D87Y |
| *S. flexneri* | ERR200392 | gyrA p.S83L |
| *S. flexneri* | ERR200393 | gyrA p.D87G |
| *S. flexneri* | ERR200402 | gyrA p.D87G |
| *S. flexneri* | ERR200403 | gyrA p.S83L |
| *S. flexneri* | ERR200405 | gyrA p.D87G |
| *S. flexneri* | ERR200413 | - |
| *S. flexneri* | ERR200414 | gyrA p.S83L |
| *S. flexneri* | ERR200415 | - |
| *S. flexneri* | ERR217012 | - |
| *S. flexneri* | ERR217013 | - |
| *S. flexneri* | ERR217015 | - |
| *S. flexneri* | ERR217016 | - |
| *S. flexneri* | ERR217022 | - |
| *S. flexneri* | ERR217023 | - |
| *S. flexneri* | ERR217024 | - |
| *S. flexneri* | ERR217026 | gyrA p.S83L |
| *S. flexneri* | ERR217028 | - |
| *S. flexneri* | ERR217030 | - |
| *S. flexneri* | ERR217031 | - |
| *S. flexneri* | ERR217032 | gyrA p.S83L |
| *S. flexneri* | ERR217033 | - |
| *S. flexneri* | ERR217047 | - |
| *S. flexneri* | ERR217080 | - |
| *S. flexneri* | ERR217081 | - |
| *S. flexneri* | ERR217084 | - |
| *S. flexneri* | ERR559526 | - |
| *S. flexneri* | S13BD02470 | gyrA p.S83L |
| *S. flexneri* | S15BD06353 | - |
| *S. flexneri* | S15BD08204 | - |
| *S. flexneri* | S15BD08762 | gyrA p.D87N, gyrA p.S83L, parC p.S80I |
| *S. flexneri* | S15BD09335 | - |
| *S. flexneri* | S15BD09453 | - |
| *S. flexneri* | S16BD00148 | gyrA p.D87N, gyrA p.S83L, parC p.S80I |
| *S. flexneri* | S16BD00463 | gyrA p.D87N |
| *S. flexneri* | S16BD01789 | - |
| *S. flexneri* | S16BD01991 | - |
| *S. flexneri* | S16BD02637 | - |
| *S. flexneri* | S16BD02856 | - |
| *S. flexneri* | S16BD03590 | gyrA p.S83L |
| *S. flexneri* | S16BD03881 | gyrA p.D87N, gyrA p.S83L, parC p.S80I |
| *S. flexneri* | S16BD04652 | - |
| *S. flexneri* | S16BD07706 | - |
| *S. flexneri* | S17BD00886 | gyrA p.D87N, gyrA p.S83L, parC p.S80I |
| *S. flexneri* | S17BD04223 | gyrA p.D87N, gyrA p.S83L, parC p.S80I |
| *S. flexneri* | S17BD04752 | gyrA p.D87N, gyrA p.S83L, parC p.S80I, parE p.S458A |
| *S. flexneri* | S17BD06364 | - |

| | | |
|---|---|---|
| *S. flexneri* | S17BD07654 | - |
| *S. flexneri* | S17BD08054 | - |
| *S. flexneri* | S17BD08237 | gyrA p.D87N, gyrA p.S83L, parC p.S80I |
| *S. flexneri* | S18BD00005 | gyrA p.D87N, gyrA p.S83L, parC p.S80I |
| *S. flexneri* | S18BD00006 | gyrA p.D87N, gyrA p.S83L, parC p.S80I, parE p.S458A |
| *S. flexneri* | S18BD01126 | - |
| *S. flexneri* | S18BD01471 | gyrA p.D87N, gyrA p.S83L, parC p.S80I |
| *S. sonnei* | ERR024604 | - |
| *S. sonnei* | ERR024605 | - |
| *S. sonnei* | ERR024606 | - |
| *S. sonnei* | ERR024607 | - |
| *S. sonnei* | ERR024608 | - |
| *S. sonnei* | ERR024609 | - |
| *S. sonnei* | ERR024610 | gyrA p.S83L |
| *S. sonnei* | ERR024611 | - |
| *S. sonnei* | ERR024612 | - |
| *S. sonnei* | ERR024616 | gyrA p.S83L |
| *S. sonnei* | ERR024617 | gyrA p.S83L |
| *S. sonnei* | ERR024618 | gyrA p.S83L |
| *S. sonnei* | ERR024619 | - |
| *S. sonnei* | ERR024620 | - |
| *S. sonnei* | ERR024621 | gyrA p.S83L |
| *S. sonnei* | ERR024622 | gyrA p.S83L |
| *S. sonnei* | ERR024625 | - |
| *S. sonnei* | ERR024626 | - |
| *S. sonnei* | ERR024627 | gyrA p.S83L |
| *S. sonnei* | ERR025682 | gyrA p.D87G |
| *S. sonnei* | ERR025683 | - |
| *S. sonnei* | ERR025685 | - |
| *S. sonnei* | ERR025686 | - |
| *S. sonnei* | ERR025687 | rpoB p.S531F |
| *S. sonnei* | ERR025689 | - |
| *S. sonnei* | ERR025690 | gyrA p.S83L |
| *S. sonnei* | ERR025691 | gyrA p.S83L |
| *S. sonnei* | ERR025692 | gyrA p.D87G |
| *S. sonnei* | ERR025693 | - |
| *S. sonnei* | ERR025695 | - |
| *S. sonnei* | ERR025697 | gyrA p.D87Y |
| *S. sonnei* | ERR025698 | - |
| *S. sonnei* | ERR025699 | gyrA p.S83L |
| *S. sonnei* | ERR025701 | - |
| *S. sonnei* | ERR025702 | gyrA p.D87G |
| *S. sonnei* | ERR025703 | gyrA p.S83L |
| *S. sonnei* | ERR025704 | - |
| *S. sonnei* | ERR025705 | - |
| *S. sonnei* | ERR025706 | - |
| *S. sonnei* | ERR025711 | gyrA p.D87Y |
| *S. sonnei* | ERR025712 | - |
| *S. sonnei* | ERR025713 | gyrA p.D87G |
| *S. sonnei* | ERR025714 | gyrA p.D87G |

| | | |
|---|---|---|
| *S. sonnei* | ERR025715 | - |
| *S. sonnei* | ERR025716 | - |
| *S. sonnei* | ERR025717 | - |
| *S. sonnei* | ERR025718 | - |
| *S. sonnei* | ERR025719 | - |
| *S. sonnei* | ERR025721 | - |
| *S. sonnei* | ERR025724 | - |
| *S. sonnei* | ERR025725 | - |
| *S. sonnei* | ERR025726 | - |
| *S. sonnei* | ERR025727 | - |
| *S. sonnei* | ERR025729 | - |
| *S. sonnei* | ERR025730 | - |
| *S. sonnei* | ERR025731 | - |
| *S. sonnei* | ERR025732 | - |
| *S. sonnei* | ERR025734 | - |
| *S. sonnei* | ERR025735 | - |
| *S. sonnei* | ERR025737 | - |
| *S. sonnei* | ERR025738 | - |
| *S. sonnei* | ERR025741 | - |
| *S. sonnei* | ERR025742 | - |
| *S. sonnei* | ERR025743 | - |
| *S. sonnei* | ERR025744 | - |
| *S. sonnei* | ERR025746 | - |
| *S. sonnei* | ERR025747 | - |
| *S. sonnei* | ERR025749 | - |
| *S. sonnei* | ERR025750 | - |
| *S. sonnei* | ERR025751 | - |
| *S. sonnei* | ERR025752 | - |
| *S. sonnei* | ERR025753 | - |
| *S. sonnei* | ERR025754 | - |
| *S. sonnei* | ERR025755 | - |
| *S. sonnei* | ERR025756 | - |
| *S. sonnei* | ERR025758 | - |
| *S. sonnei* | ERR025759 | - |
| *S. sonnei* | ERR025761 | - |
| *S. sonnei* | ERR025762 | - |
| *S. sonnei* | ERR025763 | - |
| *S. sonnei* | ERR025765 | - |
| *S. sonnei* | ERR025767 | - |
| *S. sonnei* | ERR025768 | - |
| *S. sonnei* | ERR028671 | - |
| *S. sonnei* | ERR028672 | - |
| *S. sonnei* | ERR028673 | - |
| *S. sonnei* | ERR028674 | - |
| *S. sonnei* | ERR028675 | - |
| *S. sonnei* | ERR028676 | - |
| *S. sonnei* | ERR028677 | gyrA p.S83L |
| *S. sonnei* | ERR028678 | - |
| *S. sonnei* | ERR028679 | gyrA p.S83L |
| *S. sonnei* | ERR028680 | - |

| | | |
|---|---|---|
| *S. sonnei* | ERR028681 | - |
| *S. sonnei* | ERR028684 | - |
| *S. sonnei* | ERR028685 | - |
| *S. sonnei* | ERR028686 | - |
| *S. sonnei* | ERR028687 | - |
| *S. sonnei* | ERR028688 | - |
| *S. sonnei* | ERR028689 | - |
| *S. sonnei* | ERR028690 | - |
| *S. sonnei* | ERR028691 | - |
| *S. sonnei* | ERR028692 | - |
| *S. sonnei* | ERR028693 | - |
| *S. sonnei* | ERR028694 | - |
| *S. sonnei* | ERR028695 | - |
| *S. sonnei* | ERR028697 | - |
| *S. sonnei* | ERR028699 | - |
| *S. sonnei* | ERR028700 | - |
| *S. sonnei* | ERR028702 | - |
| *S. sonnei* | ERR028703 | - |
| *S. sonnei* | ERR028704 | - |
| *S. sonnei* | ERR028705 | gyrA p.S83L |
| *S. sonnei* | ERR028706 | - |
| *S. sonnei* | ERR316291 | - |
| *S. sonnei* | ERR316306 | - |
| *S. sonnei* | ERR586833 | gyrA p.D87G, gyrA p.S83L, parC p.S80I |
| *S. sonnei* | ERR586837 | gyrA p.D87G, gyrA p.S83L, parC p.S80I |
| *S. sonnei* | ERR586838 | gyrA p.D87G, gyrA p.S83L, parC p.S80I |
| *S. sonnei* | ERR591156 | gyrA p.D87G, gyrA p.S83L, parC p.S80I |
| *S. sonnei* | ERR591166 | gyrA p.D87G, gyrA p.S83L, parC p.S80I |
| *S. sonnei* | ERR591176 | gyrA p.D87G, gyrA p.S83L, parC p.S80I |
| *S. sonnei* | ERR591179 | gyrA p.D87G, gyrA p.S83L, parC p.S80I |
| *S. sonnei* | ERR591185 | gyrA p.D87G, gyrA p.S83L, parC p.S80I |
| *S. sonnei* | ERR591188 | gyrA p.D87G, gyrA p.S83L, parC p.S80I |
| *S. sonnei* | ERR591211 | gyrA p.D87G, gyrA p.S83L, parC p.S80I |
| *S. sonnei* | ERR591218 | gyrA p.D87G, gyrA p.S83L, parC p.S80I |
| *S. sonnei* | ERR591405 | - |
| *S. sonnei* | ERR592007 | - |
| *S. sonnei* | ERR592030 | - |
| *S. sonnei* | ERR592189 | gyrA p.D87Y |
| *S. sonnei* | ERR592269 | gyrA p.S83L |
| *S. sonnei* | ERR592270 | gyrA p.D87G, gyrA p.S83L, parC p.S80I |
| *S. sonnei* | ERR708271 | - |
| *S. sonnei* | ERR708272 | gyrA p.D87Y |
| *S. sonnei* | ERR708273 | gyrA p.D87G, gyrA p.S83L, parC p.S80I |
| *S. sonnei* | ERR708274 | gyrA p.S83L |
| *S. sonnei* | ERR708275 | gyrA p.D87G, gyrA p.S83L, parC p.S80I |
| *S. sonnei* | ERR708277 | gyrA p.D87G, gyrA p.S83L, parC p.S80I |
| *S. sonnei* | ERR708278 | gyrA p.D87G, gyrA p.S83L, parC p.S80I |
| *S. sonnei* | ERR708279 | gyrA p.S83L, parC p.S80I |
| *S. sonnei* | ERR708280 | gyrA p.D87G, gyrA p.S83L, parC p.S80I |
| *S. sonnei* | ERR708281 | gyrA p.D87G, gyrA p.S83L, parC p.S80I |

| | | |
|---|---|---|
| *S. sonnei* | ERR708282 | gyrA p.D87G, gyrA p.S83L, parC p.S80I |
| *S. sonnei* | ERR708283 | gyrA p.D87G, gyrA p.S83L, parC p.S80I |
| *S. sonnei* | ERR708284 | gyrA p.D87G, gyrA p.S83L, parC p.S80I |
| *S. sonnei* | ERR708285 | gyrA p.D87G, gyrA p.S83L, parC p.S80I |
| *S. sonnei* | ERR708286 | gyrA p.D87G, gyrA p.S83L, parC p.S80I |
| *S. sonnei* | ERR708287 | gyrA p.D87G, gyrA p.S83L, parC p.S80I |
| *S. sonnei* | ERR708288 | gyrA p.D87G, gyrA p.S83L, parC p.S80I |
| *S. sonnei* | ERR708289 | gyrA p.D87G, gyrA p.S83L, parC p.S80I |
| *S. sonnei* | ERR708290 | gyrA p.D87G, gyrA p.S83L, parC p.S80I |
| *S. sonnei* | S13BD00834 | gyrA p.D87G, gyrA p.S83L, parC p.S80I |
| *S. sonnei* | S13BD00906 | gyrA p.D87G, gyrA p.S83L, parC p.S80I |
| *S. sonnei* | S13BD02106 | gyrA p.S83L |
| *S. sonnei* | S13BD02107 | gyrA p.D87G, gyrA p.S83L, parC p.S80I |
| *S. sonnei* | S13BD02670 | gyrA p.S83L |
| *S. sonnei* | S13BD02671 | gyrA p.S83L |
| *S. sonnei* | S13BD03196 | gyrA p.D87G, gyrA p.S83L, parC p.S80I |
| *S. sonnei* | S13BD03288 | gyrA p.S83L |
| *S. sonnei* | S13BD03539 | gyrA p.D87G, gyrA p.S83L, parC p.S80I |
| *S. sonnei* | S13BD04360 | gyrA p.S83L |
| *S. sonnei* | S14BD00913 | gyrA p.D87G, gyrA p.S83L, parC p.S80I |
| *S. sonnei* | S14BD03054 | gyrA p.D87G, gyrA p.S83L, parC p.S80I |
| *S. sonnei* | S14BD03397 | gyrA p.S83L |
| *S. sonnei* | S14BD03573 | gyrA p.D87Y |
| *S. sonnei* | S14BD04882 | gyrA p.S83L |
| *S. sonnei* | S14BD04971 | gyrA p.S83L |
| *S. sonnei* | S14BD05043 | gyrA p.D87G, gyrA p.S83L, parC p.S80I |
| *S. sonnei* | S15BD00569 | gyrA p.D87G, gyrA p.S83L, parC p.S80I |
| *S. sonnei* | S15BD00797 | gyrA p.D87G, gyrA p.S83L, parC p.S80I |
| *S. sonnei* | S15BD00889 | gyrA p.S83L |
| *S. sonnei* | S15BD00932 | gyrA p.S83L |
| *S. sonnei* | S15BD01283 | gyrA p.S83L |
| *S. sonnei* | S15BD02324 | gyrA p.S83L |
| *S. sonnei* | S15BD02325 | gyrA p.S83L |
| *S. sonnei* | S15BD02326 | gyrA p.S83L |
| *S. sonnei* | S15BD02407 | gyrA p.S83L |
| *S. sonnei* | S15BD02515 | gyrA p.S83L |
| *S. sonnei* | S15BD02737 | gyrA p.D87G, gyrA p.S83L, parC p.S80I |
| *S. sonnei* | S15BD02979 | gyrA p.S83L |
| *S. sonnei* | S15BD02995 | gyrA p.S83L |
| *S. sonnei* | S15BD03192 | gyrA p.S83L |
| *S. sonnei* | S15BD03801 | gyrA p.D87G, gyrA p.S83L, parC p.S80I |
| *S. sonnei* | S15BD03806 | gyrA p.D87G, gyrA p.S83L, parC p.S80I |
| *S. sonnei* | S15BD03807 | gyrA p.D87G, gyrA p.S83L, parC p.S80I |
| *S. sonnei* | S15BD03808 | gyrA p.D87G, gyrA p.S83L, parC p.S80I |
| *S. sonnei* | S15BD03850 | gyrA p.S83L |
| *S. sonnei* | S15BD04030 | gyrA p.S83L |
| *S. sonnei* | S15BD04031 | gyrA p.D87G, gyrA p.S83L, parC p.S80I |
| *S. sonnei* | S15BD04165 | gyrA p.D87G, gyrA p.S83L, parC p.S80I |
| *S. sonnei* | S15BD04166 | gyrA p.D87G, gyrA p.S83L, parC p.S80I |
| *S. sonnei* | S15BD04402 | - |

| | | |
|---|---|---|
| *S. sonnei* | S15BD04792 | gyrA p.S83L |
| *S. sonnei* | S15BD04848 | gyrA p.D87G, gyrA p.S83L, parC p.S80I |
| *S. sonnei* | S15BD05940 | gyrA p.S83L |
| *S. sonnei* | S15BD06471 | gyrA p.D87G, gyrA p.S83L, parC p.S80I |
| *S. sonnei* | S15BD07017 | gyrA p.D87G, gyrA p.S83L, parC p.S80I |
| *S. sonnei* | S15BD07161 | gyrA p.D87G, gyrA p.S83L, parC p.S80I |
| *S. sonnei* | S15BD07522 | gyrA p.D87G, gyrA p.S83L, parC p.S80I |
| *S. sonnei* | S15BD09124 | gyrA p.S83L |
| *S. sonnei* | S15BD09162 | gyrA p.D87G, gyrA p.S83L, parC p.S80I |
| *S. sonnei* | S15BD09356 | gyrA p.S83L |
| *S. sonnei* | S15BD09628 | gyrA p.S83L |
| *S. sonnei* | S15BD09656 | gyrA p.S83L |
| *S. sonnei* | S15BD10104 | gyrA p.D87G, gyrA p.S83L, parC p.S80I |
| *S. sonnei* | S15BD10108 | gyrA p.S83L |
| *S. sonnei* | S15BD10196 | gyrA p.D87G, gyrA p.S83L, parC p.S80I |
| *S. sonnei* | S15BD10296 | gyrA p.S83L |
| *S. sonnei* | S16BD00285 | gyrA p.S83L |
| *S. sonnei* | S16BD00582 | gyrA p.D87G, gyrA p.S83L, parC p.S80I |
| *S. sonnei* | S16BD00590 | gyrA p.D87G, gyrA p.S83L, parC p.S80I |
| *S. sonnei* | S16BD01339 | gyrA p.S83L |
| *S. sonnei* | S16BD01740 | gyrA p.D87G, gyrA p.S83L, parC p.S80I |
| *S. sonnei* | S16BD01790 | gyrA p.D87G, gyrA p.S83L, parC p.S80I |
| *S. sonnei* | S16BD02574 | gyrA p.D87Y |
| *S. sonnei* | S16BD03340 | gyrA p.D87G, gyrA p.S83L, parC p.S80I |
| *S. sonnei* | S16BD04069 | gyrA p.D87Y |
| *S. sonnei* | S17BD01977 | - |
| *S. sonnei* | S17BD02088 | - |
| *S. sonnei* | S17BD02164 | - |
| *S. sonnei* | S17BD02526 | - |
| *S. sonnei* | S17BD02897 | gyrA p.D87G, gyrA p.S83L, parC p.S80I |
| *S. sonnei* | S17BD03513 | gyrA p.D87G, gyrA p.S83L, parC p.S80I |
| *S. sonnei* | S17BD04438 | gyrA p.D87G, gyrA p.S83L, parC p.S80I |
| *S. sonnei* | S17BD06426 | - |
| *S. sonnei* | S17BD06464 | gyrA p.D87G, gyrA p.S83L, parC p.S80I |
| *S. sonnei* | S17BD06759 | gyrA p.D87G, gyrA p.S83L, parC p.S80I |
| *S. sonnei* | S17BD06760 | gyrA p.D87G, gyrA p.S83L, parC p.S80I |
| *S. sonnei* | S17BD06799 | gyrA p.D87G, gyrA p.S83L, parC p.S80I |
| *S. sonnei* | S17BD06801 | gyrA p.D87G, gyrA p.S83L, parC p.S80I |
| *S. sonnei* | S17BD07774 | gyrA p.D87G, gyrA p.S83L, parC p.S80I |
| *S. sonnei* | S17BD07961 | - |
| *S. sonnei* | S18BD00011 | gyrA p.D87Y |
| *S. sonnei* | S18BD00105 | gyrA p.D87G, gyrA p.S83L, parC p.S80I |
| *S. sonnei* | S18BD00654 | - |
| *S. sonnei* | S18BD01122 | gyrA p.D87G, gyrA p.S83L, parC p.S80I |
| *S. sonnei* | S18BD01231 | gyrA p.D87G, gyrA p.S83L, parC p.S80I |
| *S. sonnei* | S18BD01685 | gyrA p.D87G, gyrA p.S83L, parC p.S80I |
| *S. sonnei* | S18BD01986 | gyrA p.D87G, gyrA p.S83L, parC p.S80I |
| *S. sonnei* | S18BD02200 | - |
| *S. sonnei* | SRR1605255 | gyrA p.S83L |
| *S. sonnei* | SRR2081185 | gyrA p.D87G, gyrA p.S83L, parC p.S80I |

| | | |
|---|---|---|
| *S. sonnei* | SRR2081189 | gyrA p.D87G, gyrA p.S83L, parC p.S80I |
| *S. sonnei* | SRR2087717 | gyrA p.S83L |
| *S. sonnei* | SRR2097498 | gyrA p.D87G, gyrA p.S83L, parC p.S80I |
| *S. sonnei* | SRR3473843 | gyrA p.D87G, gyrA p.S83L, parC p.S80I |
| *S. sonnei* | SRR3473844 | gyrA p.D87G, gyrA p.S83L, parC p.S80I |
| *S. sonnei* | SRR3473847 | gyrA p.D87N, gyrA p.S83L, parC p.S80I |
| *S. sonnei* | SRR3473848 | gyrA p.D87Y |
| *S. sonnei* | SRR3473849 | gyrA p.D87G, gyrA p.S83L, parC p.S80I |
| *S. sonnei* | SRR3473850 | gyrA p.D87G, gyrA p.S83L, parC p.S80I |
| *S. sonnei* | SRR3473853 | gyrA p.D87G, gyrA p.S83L, parC p.S80I |
| *S. sonnei* | SRR3473854 | gyrA p.D87G, gyrA p.S83L, parC p.S80I |
| *S. sonnei* | SRR3473855 | gyrA p.D87G, gyrA p.S83L, parC p.S80I |
| *S. sonnei* | SRR3473856 | gyrA p.D87G, gyrA p.S83L, parC p.S80I |
| *S. sonnei* | SRR3473857 | gyrA p.D87G, gyrA p.S83L, parC p.S80I |
| *S. sonnei* | SRR3473858 | gyrA p.D87G, gyrA p.S83L, parC p.S80I |
| *S. sonnei* | SRR3473859 | gyrA p.D87G, gyrA p.S83L, parC p.S80I |
| *S. sonnei* | SRR3473860 | gyrA p.D87G, gyrA p.S83L, parC p.S80I |
| *S. sonnei* | SRR3473861 | gyrA p.D87G, gyrA p.S83L, parC p.S80I |
| *S. sonnei* | SRR3473862 | gyrA p.D87G, gyrA p.S83L, parC p.S80I |
| *S. sonnei* | SRR3474162 | gyrA p.D87G, gyrA p.S83L, parC p.S80I |
| *S. sonnei* | SRR3474164 | gyrA p.D87G, gyrA p.S83L, parC p.S80I |
| *S. sonnei* | SRR3474165 | gyrA p.D87G, gyrA p.S83L, parC p.S80I |
| *S. sonnei* | SRR3474166 | gyrA p.D87G, gyrA p.S83L, parC p.S80I |
| *S. sonnei* | SRR3474167 | gyrA p.D87G, gyrA p.S83L, parC p.S80I |
| *S. sonnei* | SRR3474168 | gyrA p.D87G, gyrA p.S83L, parC p.S80I |
| *S. sonnei* | SRR3474169 | gyrA p.D87G, gyrA p.S83L, parC p.S80I |

The first and second columns list the species and sample names, respectively. The third column lists all AMR point mutations detected with PointFinder in the corresponding sample.

**Supplementary Table S8: Detected *qnr* genes.**

| Sample name | Species | Collection | Gene | Ciprofloxacin resistance |
|---|---|---|---|---|
| ERR047236 | *S. flexneri* | Connor2015 | *qnrS1* | - |
| ERR200393 | *S. flexneri* | Connor2015 | *qnrS1* | - |
| ERR200413 | *S. flexneri* | Connor2015 | *qnrS1* | - |
| ERR217023 | *S. flexneri* | Connor2015 | *qnrS1* | - |
| ERR217024 | *S. flexneri* | Connor2015 | *qnrS1* | - |
| S13BD00302 | *S. flexneri* | NRC BEL | *qnrS1* | High-level |
| S15BD02407 | *S. sonnei* | NRC BEL | *qnrS1* | Low-level |
| S15BD02726 | *S. flexneri* | NRC BEL | *qnrS1* | Low-level |
| S15BD04031 | *S. sonnei* | NRC BEL | *qnrB4* | High-level |
| S15BD04792 | *S. sonnei* | NRC BEL | *qnrB4* | Low-level |
| S15BD06471 | *S. sonnei* | NRC BEL | *qnrB19* | High-level |
| S16BD00285 | *S. sonnei* | NRC BEL | *qnrS1* | Low-level |
| S16BD00463 | *S. flexneri* | NRC BEL | *qnrS1* | Low-level |
| S18BD00005 | *S. flexneri* | NRC BEL | *qnrS1* | High-level |
| S18BD00011 | *S. sonnei* | NRC BEL | *qnrS1* | Low-level |
| S18BD01109 | *S. flexneri* | NRC BEL | *qnrS1* | Low-level |

The first, second, and third columns list the sample name, species, and collection of the samples, respectively. The fourth column lists the detected *qnr* gene. The last column contains the result of the phenotypic testing for resistance to ciprofloxacin. A dash ('-') indicates that ciprofloxacin phenotypic testing data was not available, which was the case for samples collected from the Conner et al. [1] background collection (labeled as 'Connor2015') for which only raw sequencing data was available, in contrast to the in-house sequenced samples (labeled as 'NRC BEL') that could also be phenotypically evaluated.

**Supplementary Table S9: Classification of contigs carrying *qnr* genes.**

| Species | Sample | Gene | Contig | # plasmid hits/ # total hits |
|---|---|---|---|---|
| *S. flexneri* | S13BD00302 | *qnrS1* | NODE_61_length_24175_cov_15.511311 | 4/4 |
| *S. flexneri* | S15BD02726 | *qnrS1* | NODE_100_length_14164_cov_31.965520 | 3/3 |
| *S. flexneri* | S16BD00463 | *qnrS1* | NODE_139_length_9774_cov_3.145848 | 3/3 |
| *S. flexneri* | S18BD00005 | *qnrS1* | NODE_125_length_10163_cov_41.683041 | 6/6 |
| *S. flexneri* | S18BD01109 | *qnrS1* | NODE_109_length_13575_cov_18.220776 | 1/1 |
| *S. sonnei* | S15BD02407 | *qnrS1* | NODE_1_length_96058_cov_21.622083 | 142/147 |
| *S. sonnei* | S15BD04031 | *qnrB4* | NODE_106_length_15215_cov_11.891503 | 53/61 |
| *S. sonnei* | S15BD04792 | *qnrB4* | NODE_138_length_10323_cov_6.506865 | 1/1 |
| *S. sonnei* | S15BD06471 | *qnrB19* | NODE_271_length_2826_cov_235.451649 | 1/1 |
| *S. sonnei* | S16BD00285 | *qnrS1* | NODE_94_length_15747_cov_2.353009 | 2/3 |
| *S. sonnei* | S18BD00011 | *qnrS1* | NODE_103_length_15509_cov_18.621896 | 6/6 |

The first and second columns list the species and sample names, respectively. The third and fourth column list the gene and corresponding contigs, respectively. The fifth column lists the number of best scoring hits (according to bit-score) containing the term ‘plasmid’ in the header and the total number of hits with the given bit-score.

**Supplementary Table S10: Lineage and PG classification, and ciprofloxacin resistance for the Belgian samples and background collection.**

| Species | PG / Lineage | # Samples | | | | | | | | |
|---|---|---|---|---|---|---|---|---|---|---|
| | | Belgium | | | | | Background collection | | | |
| | | High res. | Low res. | Susc. | Unk. | Total | Res. | Susc. | Unk. | Total |
| *S. flexneri* | 1 | 2 | 3 | 3 | 0 | 8 | 0 | 0 | 26 | 26 |
| | 2 | 1 | 1 | 11 | 0 | 13 | 0 | 0 | 14 | 14 |
| | 3 | 29 | 1 | 5 | 0 | 35 | 0 | 0 | 44 | 44 |
| | 4 | 0 | 0 | 1 | 0 | 1 | 0 | 0 | 9 | 9 |
| | 5 | 0 | 0 | 0 | 0 | 0 | 0 | 0 | 6 | 6 |
| | 6 | 0 | 0 | 0 | 0 | 0 | 0 | 0 | 16 | 16 |
| | 7 | 0 | 1 | 1 | 0 | 2 | 0 | 0 | 14 | 14 |
| *S. sonnei* | I | 0 | 0 | 0 | 0 | 0 | 0 | 0 | 11 | 11 |
| | II | 0 | 0 | 0 | 0 | 0 | 0 | 0 | 33 | 33 |
| | III | 45 | 35 | 9 | 0 | 89 | 52 | 12 | 78 | 142 |
| | IV | 0 | 0 | 0 | 0 | 0 | 0 | 0 | 1 | 1 |
| | V | 0 | 0 | 0 | 0 | 0 | 0 | 3 | 0 | 3 |

Abbreviations: Phylogenetic group (PG), resistant (Res.), susceptible (Susc.), unknown (Unk.).

**References**

1. **Connor TR, Barker CR, Baker KS, Weill FX, Talukder KA, *et al.*** Species-wide whole genome sequencing reveals historical global spread and recent local persistence in Shigella flexneri. *Elife* 2015;4:1–16.
